# Scalable phosphotyrosine enrichment with SH2 superbinder enables deep profiling of EGF responses

**DOI:** 10.1038/s44318-026-00843-8

**Published:** 2026-07-03

**Authors:** Alexis T Chang, Ricard A Rodriguez-Mias, Matthew D Berg, Sophie Moggridge, Judit Villén

**Affiliations:** Department of Genome Sciences, University of Washington, Seattle, 98195 USA

**Keywords:** Methods & Resources, Post-translational Modifications & Proteolysis, Signal Transduction

## Abstract

Phosphotyrosine signaling plays a critical role in many biological processes, from cell proliferation to immune response. Despite its importance, proteomic studies of tyrosine phosphorylation have been limited in scale and throughput due to the need for specialized enrichment with costly reagents and labor-intensive protocols. To address these challenges, we developed R2HaPpY, a phosphotyrosine enrichment method that combines highly simplified phosphotyrosine superbinder reagent preparation and automated high-throughput enrichment. Our new reagent binds phosphotyrosine peptides at higher efficiency than other enrichment reagents and reduces both cost and preparation time by 20-fold. We generalized the R2HaPpY method to samples of low and high levels of phosphotyrosine. We benchmarked biological application to study EGF signaling dynamics in HeLa cells. Using only ~1 mg of input peptides, we detect and quantify 1651 unique phosphotyrosine sites. These include 878 regulated pY sites, many of which are novel or not previously annotated as EGF-responsive. Our results reveal differential temporal regulation and represent the largest phosphotyrosine dataset of cellular response to EGF stimulation to date. This streamlined, cost-effective, and sensitive method enables quantitative mapping of tyrosine phosphorylation dynamics at a scale of hundreds of samples, facilitating integration of phosphotyrosine signaling into multiomic studies across diverse biological systems and disease states.

## Synopsis

Proteomic studies of tyrosine phosphorylation have been hampered by need for labor-intensive protocols and costly enrichment reagents. A new method, R2HaPpY, enables large-scale tyrosine phosphoproteomics studies by simplifying bead preparation, automating phosphopeptide enrichment, and lowering costs, and yields the deepest temporal phosphotyrosine dataset of EGF signaling reported to date.

- R2HaPpY simplifies the preparation of phosphotyrosine superbinder capture reagent, expediting this process from multiple days to one hour.
- The sensitivity of the R2HaPpY method is comparable to phosphotyrosine enrichment with antibodies and the reagent cost is ~20 times lower.
- Phosphotyrosine peptide enrichment with R2HaPpY is highly reproducible, exceeds 99% efficiency across a wide range of sample inputs, and runs in an automated format that scales to hundreds of samples.
- Applied to EGF-stimulated cells, R2HaPpY quantified 1651 phosphotyrosine sites—878 of them dynamically regulated—and resolves how individual proteins are regulated differently at distinct sites.
- The dataset includes 70 previously unreported phosphotyrosine sites, such as pY27 in the E3 ligase RNF126, pY21 in SUMO1, pY197 in HGS, and pY4 in transketolase (TKT).

## Abstract (web summary)

The new method R2HaPpY facilitates large-scale tyrosine phosphoproteomics studies by simplifying bead preparation and automating phosphopeptide enrichment.

## Graphical abstract

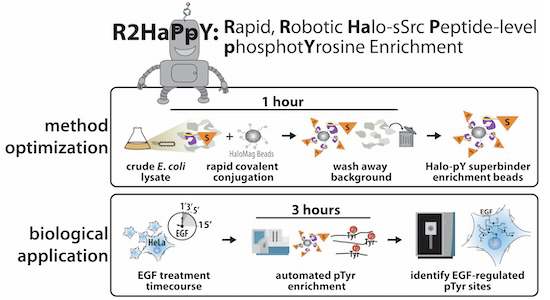

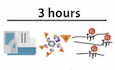

## Introduction

Tyrosine phosphorylation (pY) is a functionally critical component of the phosphoproteome. Despite being far less abundant than serine and threonine phosphorylation (Olsen et al, 2006; Sharma et al, 2014), pY governs intercellular communication and coordinates proliferation, differentiation, migration, and tissue organization (Hunter, 2009; Lim and Pawson, 2010; Lemmon and Schlessinger, 2010). Dysregulated pY signaling drives cancer and other diseases (Blume-Jensen and Hunter, 2001; Lemmon and Schlessinger, 2010; Hunter, 2000; Du and Lovly, 2018). Tyrosine kinase inhibitors are among the most successful targeted therapies in oncology (Cohen et al, 2022; Hochhaus et al, 2017), and mapping pY regulation across tumors (Rikova et al, 2007) and cellular states promises both mechanistic insight and new therapeutic opportunities (Huang et al, 2007; Yoshida et al, 2014; Abe et al, 2017). Realizing this potential, however, requires the ability to measure pY at sample scales that are now routine for serine and threonine phosphorylation.

Phosphoproteomics has matured rapidly: studies now routinely quantify thousands of phosphosites across temporal perturbations (Olsen et al, 2006; Humphrey et al, 2015; Leutert et al, 2023), drug panels (Klaeger et al, 2017; Zecha et al, 2023), and large patient cohorts (Gillette et al, 2020; Krug et al, 2020). This progress, however, has been overwhelmingly serine- and threonine-centric (see Results). Tyrosine phosphorylation is consistently under-sampled because the antibody-based reagents required for pY enrichment (Rush et al, 2005; Boersema et al, 2010) are costly or labor-intensive to prepare, placing large-scale pY profiling out of reach for most laboratories. A second distinct constraint is sample input: pY-specific enrichments typically require several milligrams of tryptic peptide per sample (Boersema et al, 2010; Bian et al, 2016; Dong et al, 2017; Rush et al, 2005; Rikova et al, 2007), restricting application in sample-limited contexts. Consequently, most large-scale phosphoproteomics efforts omit pY enrichment, including major surveys of cell lines (Frejno et al, 2020; Shi et al, 2025), cancer biospecimens (Mertins et al, 2016; Gillette et al, 2020; Krug et al, 2020; Petralia et al, 2020), and drug responses (Zecha et al, 2023; Klaeger et al, 2017; Lee et al, 2023). This omission has limited both the biological understanding of pY signaling and the data available to model it computationally. Predictive models of phosphosite function, kinase activity, and disease-mutation impact perform well on pS/pT sites but consistently underperform on pY (Ochoa et al, 2020; Jiang et al, 2025; Müller-Dott et al, 2025).

An alternative pY enrichment replaces antibodies with engineered Src Homology 2 (SH2) domains that bind pY with comparable affinity, such as the Src SH2 superbinder (sSrc) (Kaneko et al, 2012). sSrc can be recombinantly expressed in *Escherichia coli*, reducing reagent cost, and has been used to map pY across diverse contexts (Bian et al, 2016; Martyn et al, 2022; Yao et al, 2018, 2019; Kong et al, 2022; Tong et al, 2017; Dong et al, 2017; Chua et al, 2020; Chua and Salomon, 2021; Kong et al, 2020). However, preparing sSrc-conjugated beads still requires affinity purification, buffer exchange, and multi-step conjugation chemistry, a workflow that demands days of hands-on time and specialized expertise. Despite a decade of availability, sSrc-based enrichments have not been adopted beyond the laboratories that developed them, and pY studies still analyze roughly an order of magnitude fewer samples than their global phosphoproteomic counterparts (see Results). Closing this gap requires a practical advance: a tyrosine phosphoproteomics workflow simple and affordable enough for routine adoption at the sample scales now standard for global phosphoproteomics.

To address this bottleneck, we fused the Src SH2 pY superbinder (Kaneko et al, 2012) to HaloTag (Los et al, 2008) to enable single-step covalent conjugation directly from crude *E. coli* lysate, collapsing the multi-step bead-preparation workflow into a single operation. Combined with an automated 96-well enrichment protocol, the resulting method—R2HaPpY (rapid robotic Halo-sSrc peptide-level phosphotyrosine enrichment)—reduces reagent cost up to 20-fold relative to commercial pY antibodies and cuts hands-on preparation time from days to one hour relative to existing sSrc reagents, while matching or exceeding their sensitivity. Halo-sSrc beads are stable for at least four months and perform robustly across a wide range of peptide inputs, making the method practical for routine large-scale pY profiling.

We applied R2HaPpY to profile epidermal growth factor (EGF) signaling dynamics in HeLa cells across five time points with six replicates per condition, using ~1 mg of input peptide per replicate. R2HaPpY identified 1651 pY sites, of which 878 were significantly regulated across the time course. This represents the largest temporal pY dataset of EGF stimulation reported to date. Beyond recapitulating known EGF signaling, R2HaPpY reproducibly identified 70 EGF-responsive pY sites not previously annotated in PhosphoSitePlus, many at low abundance. The dataset’s depth resolved distinct temporal regulation of multiple pY sites within individual proteins, illustrating the site-level heterogeneity accessible at this measurement scale. By making large-scale pY profiling practical for standard proteomics laboratories, R2HaPpY enables tyrosine phosphoproteomics to be routinely integrated into multiomic studies from which it has largely been excluded.

## Results and discussion

### Current tyrosine phosphoproteomics methods are not being scaled up

**Figure 1:**
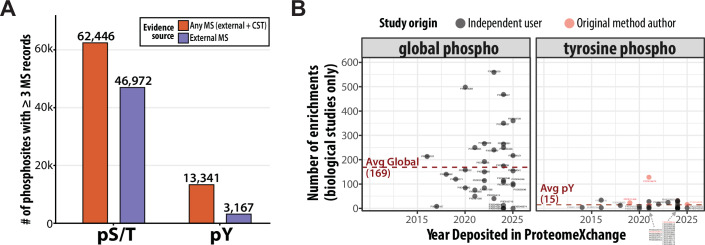
Tyrosine phosphoproteomics remains bottlenecked. (**A**) Evidence source distribution for human phosphorylation sites in PhosphoSitePlus with at least three high-throughput mass spectrometry observations, separated by phospho-acceptor residue. Orange indicates sites evidenced by either internal Cell Signaling Technology, Inc. (CST) studies or external literature. Purple indicates sites evidenced by at least three external studies. Numbers above bars indicate the number of phosphosites in each evidence type category. (**B**) Dot plot indicates enrichment counts for phosphoproteomics studies deposited in ProteomeXchange between 2015 and 2025, separated into global phosphopeptide enrichments (left) and tyrosine-specific enrichments (right). Studies were identified by the keywords “phosphoproteomics” and “tyrosine”, ranked by raw file count, and manually inspected to quantify the number of enrichments per study; accession level annotations are provided in Dataset EV1. Points are labeled by ProteomeXchange experiment accession. Horizontal lines indicate mean enrichments per study for each category. Tyrosine-specific studies run approximately tenfold fewer enrichments on average than global studies. Points colored pink in the pY plot refer to biological studies run by authors of an enrichment method. Source data are available online for this figure.

To assess whether the broader research community has adopted phosphotyrosine enrichment at scales comparable to global phosphopeptide enrichment, we examined the evidence underlying PhosphoSitePlus, a primary repository of human phosphorylation sites (Data ref: Hornbeck et al, 2012, 2015). The pS/pT and pY evidence bases differ markedly in composition. Of sites with three or more supporting mass spectrometry (MS) records, 46,972 pS or pT sites (75%) carry corroborating evidence from external (non-Cell Signaling Technology, Inc. (CST)) studies. In contrast, only 3167 pY sites (24%) meet the same criterion, indicating that most cataloged pY sites lack independent external corroboration (Fig. 1A). This asymmetry demonstrates that pY-specific enrichment has not been adopted by the external community at a scale comparable to global phosphopeptide enrichment. As a corollary, the publicly re-analyzable pY evidence base available for training predictive models of kinase activity, site function, and disease-mutation impact remains a small fraction of the evidence base available for pS or pT sites.

To extend this analysis beyond a single repository, we surveyed phosphoproteomic studies deposited in ProteomeXchange (Vizcaíno et al, 2014) between 2015 and 2025, identified by the keywords “phosphoproteomics” and “tyrosine” and manually annotated for enrichment type. Tyrosine-specific studies averaged 15 enrichments per study, compared with 169 enrichments per global phosphopeptide study—an order of magnitude difference (Fig. 1B). Even this gap understates the disparity among general users: the sole pY study exceeding 40 enrichments was conducted by a developer of the superbinder reagent and an associated enrichment workflow (Mirhadi et al, 2022; Tong et al, 2017). Together with the PhosphoSitePlus analysis, these data establish that pY-specific enrichment remains a niche workflow within the proteomics community, in marked contrast to the routine adoption of global phosphopeptide enrichment. The bottleneck is not biological interest but practical access; specifically, the cost and hands-on time required to run pY enrichments at the scale demanded by modern clinical and multiomic studies remain prohibitive.

### HaloLink^TM^ bead chemistry simplifies sSrc bead preparation and outperforms NHS chemistry

**Figure 2:**
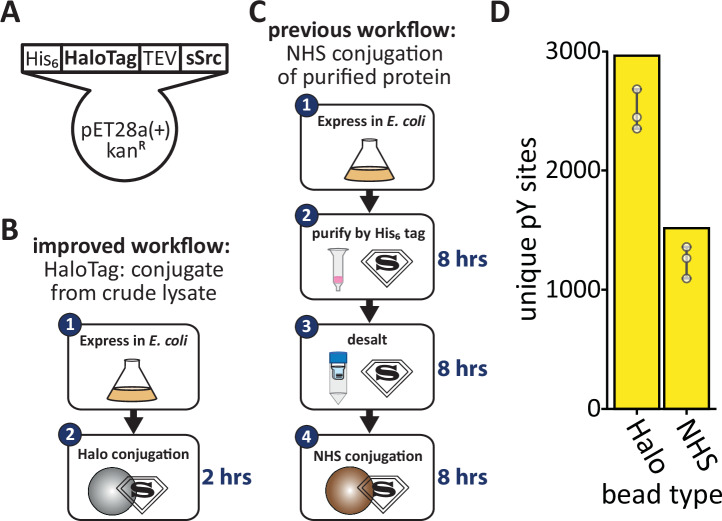
Halo-sSrc is easy to prepare and outperforms NHS conjugation chemistry. (**A**) Construct design of Halo-sSrc within the *E. coli* expression vector. From N- to C- terminus, the translated protein under the control of an IPTG inducible promoter consists of a His_6_ tag, serine-serine-glycine linker, a HaloTag (297 amino acids, 33 kDa), serine-glycine-serine-glycine linker, tobacco etch virus (TEV) protease cleavage site (ENLYFQ), serine-glycine-serine linker, and the sSrc protein (109 amino acids, 12.4 kDa). (**B**) Schematic of the improved bead preparation protocol, where purification and conjugation of Halo-sSrc are performed in a single step by mixing Magne HaloTag beads with crude *E. coli* lysate containing overexpressed Halo-sSrc and washing away unbound material with PBS. (**C**) Schematic of the previously published bead preparation workflow requiring prepurification of His_6_-sSrc by metal affinity chromatography, protein desalting, and spin concentration prior to conjugation to magnetic NHS-activated beads. (**D**) Performance of Halo-sSrc beads compared to NHS-sSrc beads for their ability to enrich pY peptides from 1 mg of pervanadate-treated HeLa cell digests in triplicate. Height of yellow bars indicate net numbers of unique pY sites detected across all replicates, points indicate the number of unique pY sites detected in each replicate and the black error bar represents range of replicate measurements.

**Figure EV1:**
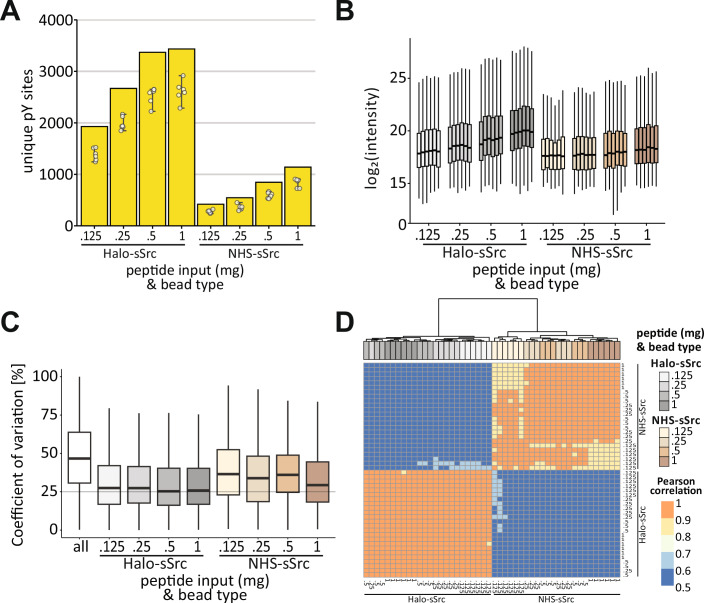
Halo-sSrc outperforms NHS-sSrc beads for pY peptide enrichment depth and reproducibility. (**A**) Bar height indicates the total count of unique phosphotyrosine (pY) sites detected across all technical replicates (*n* = 6). Points indicate pY-site counts from individual replicates; whiskers indicate the range (minimum-maximum) across replicates. The amount of pervanadate-treated HeLa peptides is indicated on the x-axis and ranges from 0.125 to 1 mg. Halo-sSrc beads are compared with NHS-sSrc beads as indicated by “Halo-sSrc” and “NHS-sSrc” on the x-axis. (**B**) Boxplot indicates log₂-transformed MS intensities of pY sites per peptide input amount and bead type (*n* = 6 technical replicates). Boxes show the median and 25th–75th percentiles; whiskers extend to 1.5× the interquartile range; outliers are not shown. (**C**) Boxplot indicates distributions of the percent coefficient of variation of pY site intensity across six technical replicates within each peptide input amount for each bead type (Halo-sSrc vs. NHS-sSrc). A combined distribution of variation considering all samples is shown at the left in white. Box, whisker, and outlier definitions as in panel (**B**). (**D**) Heatmap shows Pearson correlation (*r*) of log_2_-transformed pY site intensities between replicates of each peptide input amount and bead type, computed on pairwise complete observations.

To develop a simple, cost-effective, and scalable pY enrichment reagent, we fused the pY superbinder Src SH2 domain (sSrc) (Kaneko et al, 2012) C-terminal to the HaloTag, combining sSrc purification and conjugation into a single step from crude *E. coli* lysate (Fig. 2A,B; DNA and amino acid sequences in Appendix). Bead preparation takes 1–2 h, compared with 3 days of hands-on time for the previously published NHS-conjugated sSrc workflow (Chang et al, 2023) (Fig. 2C). Benchmarked side-by-side on a tryptic digest of pervanadate-treated HeLa lysate, Halo-sSrc beads yielded more pY peptide identifications and higher pY signal than NHS-sSrc beads (Fig. 2D; Appendix Fig. S1A–F) and maintained this advantage across a range of peptide inputs (Fig. EV1A–D). We attribute this improvement to stabilization of sSrc by the Halo fusion (N Peterson and Kwon, 2012), a quick and gentle conjugation procedure, and to site-specific, single-point conjugation through HaloTag—in contrast to NHS chemistry, which reacts non-specifically with exposed lysines, including one within the pY-peptide recognition interface (Appendix Fig. S2).

**Figure EV2:**
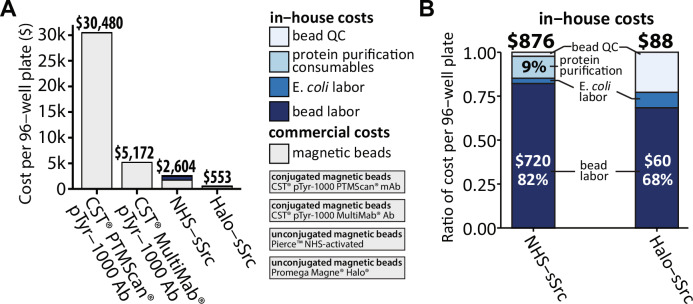
Halo-sSrc beads are the most economical pTyr enrichment option and do not require traditional protein purification. (**A**) Cost per 96 samples for four pTyr-capture reagents (left to right): Cell Signaling Technologies® (CST) PTMScan® pTyr-1000 mAb, CST MultiMab® pTyr-1000 magnetic bead conjugate, Pierce™ NHS-activated magnetic sSrc bead conjugate, and Promega Magne® HaloTag® sSrc bead conjugate. Commercial costs include the purchase of antibody-conjugated magnetic beads or unconjugated magnetic beads for in-house attachment of the superbinder Src SH2 domain (sSrc). Cell Signaling Technology® (CST®) sells PTMScan® antibody-conjugated magnetic beads for $2540 per 800 µL of bead slurry (Catalog #8803). Following the EasyAb-recommended use of 100 µL CST PTMScan® magnetic bead slurry per sample, the cost is $30,480 per 96 samples (preprint: Jayavelu et al, 2024). CST® sells 400 µL of magnetic beads conjugated pTyr-1000 MultiMab® for $431 and prescribes use at a ratio of 1:20, which equates to 50 µL of bead slurry per 1 mL sample. This translates to $53.80 per sample or $5172 for 96 samples and does not require any in-house labor costs. Preparation of NHS-sSrc beads according to our 2023 publication for 96 samples requires an estimated $2604, of which 34% ($876) is accrued from in-house costs (Chang et al, 2023). In contrast, Halo-sSrc beads cost $553 for 96 samples, of which 16% ($88) is related to in-house bead preparation under the same assumptions. Additional cost calculation details are provided in Appendix Text S3. (**B**) Breakdown of in-house costs for preparation of NHS-sSrc or Halo-sSrc magnetic bead conjugates for 96 samples (see Methods for cost calculation). Commercial antibody-conjugated beads lack in-house preparation costs and are therefore excluded. In-house costs for NHS-sSrc beads and Halo-sSrc beads are $876 and $88, respectively. We show the breakdown of in-house bead costs according to our estimates (Appendix Text S3), but note that each lab has unique cost structures.

**Figure EV3:**
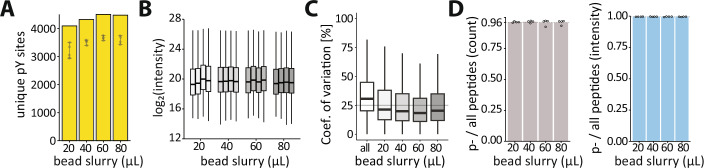
Amount of beads for optimal enrichment capacity and reproducibility. To assess the impact of bead amount on pY enrichment capacity, peptides (0.5 mg at 0.6 mg/mL) from pervanadate-treated HeLa cells were enriched with varying bead volumes. Four technical replicates were measured for each bead quantity. (**A**) Bar height indicates the total count of unique pY sites enriched across all technical replicates (*n* = 4). Points indicate counts of unique pY sites enriched per replicate for each bead slurry volume. Whiskers indicate the minimum-maximum range across replicates. (**B**) Boxplot indicates intensity distributions of pY peptides per replicate for each bead slurry volume (*n* = 4 technical replicates per condition). Boxes show the median and 25th–75th percentiles; whiskers extend to 1.5× the interquartile range; outlier points are not shown. (**C**) Boxplot indicates distributions of percent coefficient of variation for pY peptides across replicates for each bead slurry volume. The white boxplot on the left labeled “all” indicates the variation of individual pY peptides across all bead slurry volumes. Outliers are not shown; box and whisker definitions as in (**B**). (**D**) Measurement of sample purity. Bar height indicates fraction of phosphopeptides out of all peptides detected by either count (left) or intensity (right) for each bead slurry volume. Points indicate phosphopeptide purity for individual replicates. The horizontal gray dotted line in each plot indicates the minimum average purity observed for any bead slurry volume.

Beyond its simplicity and performance, R2HaPpY is substantially cheaper than existing approaches. At the time of writing, preparing beads for 96 enrichments costs approximately $553 with Halo-sSrc, compared with $2604 for NHS-sSrc and $5172 to $30,480 for Cell Signaling Technology® p-Tyr-1000 MultiMab® and PTM-Scan® HS p-Tyr-1000 antibody-conjugated magnetic beads, respectively (Fig. EV2A,B; Appendix Text S3). These savings arise from four factors: (i) reduction of bead preparation from 3 days to 1–2 h; (ii) two-fold lower bead slurry requirement per sample relative to NHS-sSrc (Fig. EV3A–D); (iii) lower per-unit cost of Promega Magne HaloTag beads relative to NHS-activated magnetic beads; and (iv) elimination of affinity resin and centrifugal filters required for His-Tag purification and buffer exchange. The Halo-sSrc reagent improves accessibility and affordability of pY peptide enrichments by omitting prepurification of superbinder. Detailed protocol and KingFisher method files are available at protocols.io (see Data availability).

We previously showed that NHS-conjugated sSrc beads match or exceed the pY site yield of the commercial CST® p-Tyr-1000 MultiMab® antibody (Chang et al, 2023). Together with our benchmarking of Halo-sSrc against NHS-sSrc here (Fig. 2D), this establishes a transitive performance hierarchy in which Halo-sSrc meets or exceeds CST antibody performance at a fraction of the cost.

### Enrichment with Halo-sSrc is robust to various peptide concentrations and phosphorylation amounts

**Figure 3:**
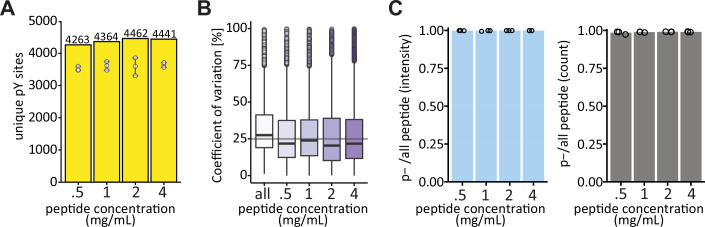
Enrichment with Halo-sSrc is robust to various peptide concentrations. (**A**) Bar plots show counts of unique pY sites aggregated from three technical replicates across four different peptide input concentrations, keeping total peptide amount constant at 0.5 mg per replicate. Peptides came from pervanadate-treated HeLa cells. Points indicate counts of unique phosphosites per replicate at each of the four peptide input concentrations. Whiskers indicate the range of replicate measurements. Height of bar indicates net unique pY sites from all three replicates. Numbers above bars indicate counts of unique pY sites. (**B**) Boxplot indicates distributions of the percent coefficient of variation of pY site intensities between triplicate measurements within a peptide input concentration. A combined percent coefficient of variation across all samples is included at left in white. Intensities of individual phosphosites were summed from all precursors with confident site localization. Intensities were median-normalized globally prior to analysis of intensity variation. The horizontal line indicates 25% CV for reference. The lower and the upper hinges of the boxes correspond to the 25th and 75th percentiles, and the bar in the box to the median. The upper and lower whiskers extend from the largest and lowest values, respectively, but no further than 1.5× the IQR from the hinge. Points indicate outliers. The number of observations (*n*) are equivalent to pY site counts as defined in panel (**A**). (**C**) Bar plots indicate sample purity defined by the relative enrichment of phosphopeptides and calculated as the intensity (left) or count (right) of phosphopeptides relative to all peptides detected. The height of bars indicates average enrichment efficiency across triplicates, and points indicate enrichment efficiencies of individual replicates.

We next asked whether Halo-sSrc enrichment is robust to variation in peptide input and concentration, which is important for high-throughput workflows constrained to the 1 mL volume of 96-well plates. Most pY enrichment protocols start from several milligrams of input peptide (preprint: Jayavelu et al, 2024; Chua et al, 2020; Bian et al, 2016; Oyama et al, 2009; Boersema et al, 2010) and accommodating this range within the plate format requires working across a wide concentration window. We therefore tested Halo-sSrc enrichment on tryptic digests of pervanadate-treated HeLa cells spanning concentrations from 0.5 to 4 mg/mL (Fig. 3A–C). Across all concentrations, enrichment efficiency exceeded 99% by intensity, more than 3300 pY sites were detected per replicate, and replicates were highly reproducible (median coefficient of variation (CV) <25%) (Fig. 3A–C).

Because pervanadate elevates tyrosine phosphorylation abundance beyond physiological levels, we repeated this test on untreated HeLa cells to confirm performance in a low-pY regime more representative of biological samples. Using 4 mg of peptide input at 1 or 4 mg/mL (in duplicate), we again observed ~99% enrichment efficiency, with modest gains in pY site recovery and reproducibility at higher concentration (Appendix Fig. S3A–D). Together, these results indicate that Halo-sSrc enrichment is robust across both dilute and concentrated peptide solutions and across high- and low-pY samples, providing flexibility in input handling within the 96-well format.

### Halo-sSrc beads are stable for ≥4 months

A robust enrichment method requires shelf-stable reagents that can be prepared in large batches. To benchmark bead reproducibility and storage, we repurposed a yeast strain expressing inducible viral Src (vSrc) kinase (Bradley et al, 2024) with high pY levels as a scalable, low-cost quality-control sample. Starting from only 0.25 mg of yeast input, this sample typically yields over 3000 unique pY peptides per enrichment (Appendix Fig. S4A,B), providing a sensitive and economical readout of bead performance and capacity.

**Figure EV4:**
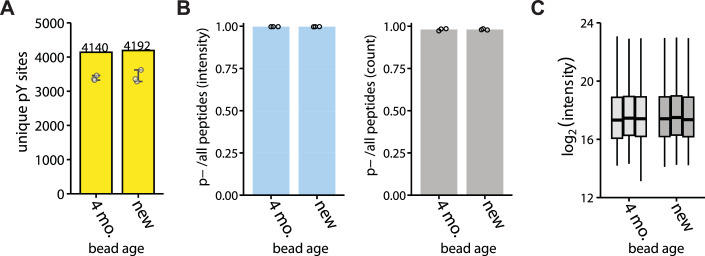
Halo-sSrc beads retain pY-enrichment functionality after 4 months of storage at 4 °C. Halo-sSrc beads stored at 4 °C for 4 months were compared with freshly prepared Halo-sSrc beads (*n* = 4 technical replicates per condition). (**A**) Counts of unique phosphotyrosine sites per condition are indicated by bar height, while counts per replicate are indicated by points. Whiskers indicate minimum-maximum range across replicates. (**B**) Enrichment purity for new and 4-month-old Halo-sSrc beads, measured as the ratio of phosphorylated peptides in the sample based on count (left) and intensity (right). Bar height indicates the mean across replicates; points represent individual replicates. (**C**) Distribution of pY peptide intensities across the four replicates for new and 4-month-old Halo-sSrc beads. Box and whisker definitions as in Fig. EV3B.

Using this control, we assessed batch-to-batch reproducibility across seven independently prepared Halo-sSrc bead preparations and observed ≥3000 unique pY peptides and >98% enrichment efficiency in every batch (Appendix Fig. S4C,D). To evaluate the shelf life, we compared freshly prepared beads to beads stored for four months at 4 °C in PBS pH 7.4. Aged and fresh beads showed comparable specificity and capacity, yielding an average of 3400 unique pY peptides per replicate with similar intensity distributions (Fig. EV4A–C). Together, these results indicate that Halo-sSrc bead preparation is reproducible and that the beads retain activity for at least four months under refrigeration.

Beyond bead quality control, the vSrc yeast lysate can serve as a standardized control during large-scale experiments—monitoring enrichment efficiency, optimizing LC gradients and MS methods, and tracking instrument performance across large batches. It is markedly cheaper and easier to produce than the mammalian cell controls used in prior work (Rush et al, 2005; Sharma et al, 2014; Bian et al, 2016; Dong et al, 2017; Tong et al, 2017; Yao et al, 2018, 2019; Chua et al, 2020; Kong et al, 2022; Martyn et al, 2022; Chang et al, 2023).

### Deep phosphotyrosine profiling of EGF-stimulated HeLa cells

To benchmark R2HaPpY on a well-characterized signaling pathway, we profiled epidermal growth factor receptor (EGFR) signaling in HeLa cells. EGFR is a prominent drug target in cancer (Cohen et al, 2022; Huang et al, 2007), particularly non-small cell lung cancer (Rikova et al, 2007; Yoshida et al, 2014) and its signaling dynamics have been characterized by phosphoproteomics in prior studies (Blagoev et al, 2004; Rush et al, 2005; Olsen et al, 2006; Guo et al, 2008; Yoshida et al, 2014; Reddy et al, 2016) providing both a reference for evaluating sensitivity and an opportunity to identify regulated pY sites missed by earlier approaches. HeLa cells were stimulated with EGF and harvested at 1, 3, 5, and 15 min post-stimulation, alongside untreated controls (*n* = 6 biological replicates per condition).

**Figure 4:**
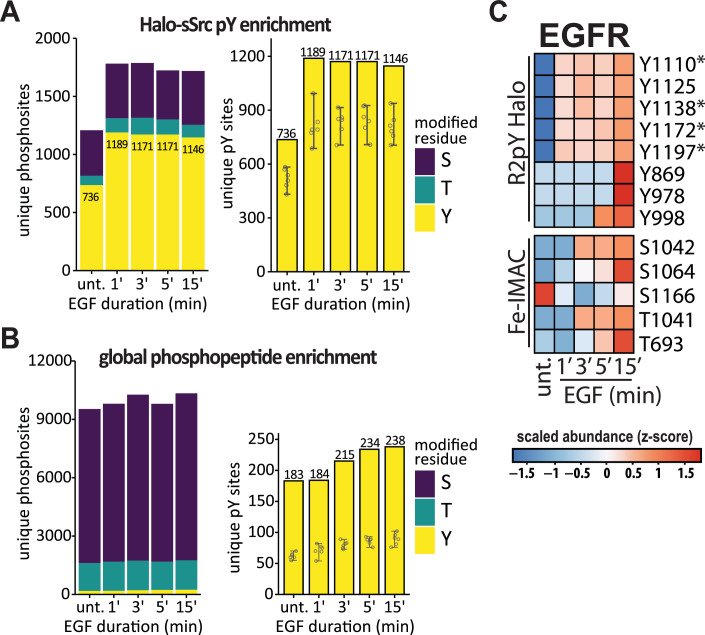
Overview of EGF-stimulated HeLa phosphopeptide enrichments. (**A**) Stacked bar plot indicates counts of unique phosphosites following pY-specific enrichment using Halo-sSrc reagent. Identity of phosphorylated amino acid is indicated by purple (pS), teal (pT), or yellow (pY). Numbers indicate counts of unique pY sites across all six replicates. The right plot only shows counts of unique pY sites. Points indicate unique sites per replicate, while whiskers indicate the range of individual replicates. The height of the yellow bar indicates the total unique pY sites across all six replicates. (**B**) Stacked bar plot indicates counts of unique phosphosites following global phosphopeptide enrichment with Fe^3+^ metal affinity (Fe-IMAC). Identity of phosphorylated amino acid is indicated by purple (pS), teal (pT), or yellow (pY). The right plot shows counts of only unique pY sites. Numbers indicate counts of unique pY sites across all six replicates. Points, whiskers, error bar, and height of the yellow bar indicate same as in (**A**). (**C**) Heatmap representing the temporal response of EGF-regulated phosphosites on epidermal growth factor receptor (EGFR). Phosphosite intensities were averaged across replicates and scaled by z-score. EGF-regulated pY sites from R2HaPpY are displayed on top, while pSer and pThr sites from global phosphopeptide (Fe-IMAC) enrichment are displayed on the bottom. Asterisks indicate the four pY sites on EGFR detected in both Fe-IMAC and pY enrichment.

In total, we identified 1651 unique pY sites across the time course, with 763–1189 unique pY sites per condition (Fig. 4A; Dataset EV2). Per-replicate depth averaged close to 1000 pY sites from only 1.3 mg of input peptide—substantially deeper than comparable label-free data-dependent acquisition (DDA) pY studies, which typically report 197–720 pY sites per replicate from 2 to 5 mg of input (preprint: Jayavelu et al, 2024; Dong et al, 2017). We also enriched the global phosphoproteome in parallel using Fe^3+^ immobilized metal affinity chromatography (Fe-IMAC). Fe-IMAC recovered 80% fewer unique pY sites per condition than Halo-sSrc enrichment (Fig. 4B) and was less sensitive in distinguishing EGF treatment durations (Appendix Fig. S5). The coverage difference is consequential at the protein level: Fe-IMAC quantified four regulated pY sites on EGFR, while Halo-sSrc quantified 8 (Fig. 4C). Together, these results show that R2HaPpY captures time-resolved EGF signaling at depth unavailable from global phosphoproteomics alone.

To identify EGF-responsive pY sites, we first required each site to be measured in at least 50% of replicates of at least one condition, yielding a filtered set of 1122 unique pY sites (629–1001 per condition). Filtering also lowered the estimated false discovery rate of pY peptides from 4.1 to 0.7%. We used six biological replicates per condition rather than the more common three, as phosphosite-level quantification relies on single-peptide measurements and is noisier for low-abundance sites—a consideration relevant to the novel and lower-abundance pY sites analyzed in later sections.

We then tested for EGF-responsive sites by Welch’s *t*-test between each EGF time point and untreated cells, using imputation for condition-wise missing values as described in Methods. Because proteome-wide abundance changes over 1–15 min of EGF stimulation are minimal, we did not adjust pY intensities for protein-level changes (Appendix Text S2). Relative to untreated, 78% of filtered pY sites changed significantly at one or more time points (Benjamini–Hochberg adjusted *P* ≤ 0.05), with 48–52% upregulated and 7–9% downregulated at any individual time point—consistent with predominantly increased phosphosite abundances reported in prior EGF studies (Reddy et al, 2016; Dong et al, 2017; Blagoev et al, 2004).

### R2HaPpY captures more EGF-responsive pY sites than previously detected

Next, we compared the pY EGF responsive sites we detected to other studies and databases. Recently, Jayavelu et al (2024) developed EasyAb (preprint: Jayavelu et al, 2024), an antibody-based enrichment method which enabled them to quantify more than 1000 pY sites from label-free, unfractionated analysis of EGF-stimulated HeLa cells. Compared to this method, R2HaPpY quantifies more pY sites per replicate and identifies ten times more EGF-regulated pY sites (878 regulated sites using R2HaPpY compared to 84 regulated sites with EasyAb). We observe many of the same regulated sites that Jayavelu et al (2024) detected, including known pY sites on EGFR (Fig. 4C) and on MET, PTPN11, GSK3B, and STAT5 (Appendix Fig. S6A). This also includes a novel pY site on VAV2 they identified with the EasyAb method (Appendix Fig. S6B). We identified these regulated sites and many more using half the input material and 20 times cheaper.

We also compared our data to a time course study of early EGF signaling in MCF-10A cells by Reddy et al that used a combination of three anti-pY antibodies and tandem mass tag (TMT) multiplexing for quantification (Reddy et al, 2016). Reddy et al quantified 159 pY sites using a 100 nM EGF stimulation over an 80 s time course. While we quantified more than ten times the number of pY sites, we did observe many of the sites detected by Reddy et al, with over 50% of the pY sites they detected also being quantified in our dataset (91/159 pY sites). The site overlap with a related but distinct experimental setup indicates R2HaPpY recovers a substantial fraction of pY signaling events detected by antibody-based methods without needing TMT multiplexing for sensitivity.

**Figure EV5:**
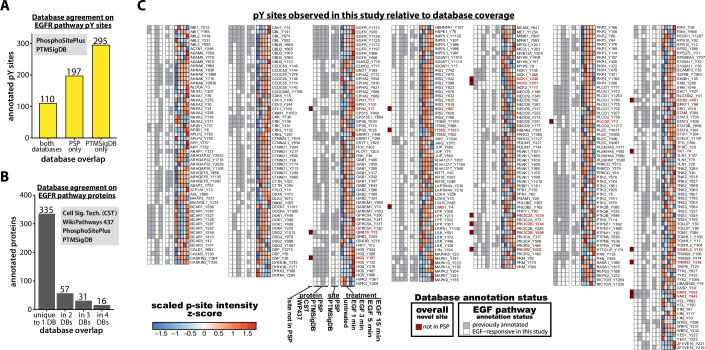
Comparison of pY sites across four databases: EGF response signatures. (**A**) EGF response signatures from two site-level databases, PhosphoSitePlus and PTMSigDB, contain 602 unique phosphotyrosine sites combined. Comparison of the EGF response signatures from each database shows limited overlap. (**B**) Overlap of proteins shared among four different protein-level databases containing the EGF signaling response. Databases include Cell Signaling Technologies (CST) ErbB/HER signaling, WikiPathways #437 (WP437) EGF/EGFR signaling, PhosphoSitePlus (PSP) EGF perturbation, and PTMSigDB EGFR signaling pathway. The x-axis indicates the number of databases in which the protein was included, while the y-axis indicates the number of proteins with the specified overlap. Sixteen proteins were present in all four databases. (**C**) Temporal response of regulated pY sites observed in this study. Annotation status in any of the four databases at either protein- or site-level is indicated by gray boxes. Novel pY sites found on proteins included in at least one of the four databases are indicated by dark red boxes. pY site intensities were averaged across replicates and then scaled by row z-score. Rows represent individual phosphosites, while columns represent EGF stimulation duration. Rows are annotated with gene name and pY site location within the full-length protein.

**Figure 5:**
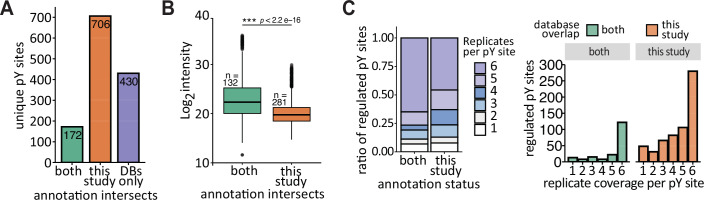
Examination of expanded phosphotyrosine profiling by R2HaPpY. (**A**) Bar plots indicate counts of unique pY sites measured in this study that were previously annotated by at least one site-level database (green) or not previously annotated by either database (orange). For comparison, the purple bar indicates pY sites annotated in either database but not observed to be significantly regulated in this study (purple). (**B**) Boxplots indicate distributions of EGF-regulated pY site intensities identified in this study that were previously annotated in EGF response signatures from either PhosphoSitePlus (Data ref: Hornbeck et al, 2012) or PTMSigDB (Data ref: Krug et al, 2019; Data ref: Perturbation Site Database, 2018) (green) or not previously annotated (orange). Only sites measured in all six replicates of a condition were included to limit the comparison to well-measured sites; *n* indicates the number of unique pY sites. Average intensity of pY sites unique to this study was 4.5- to 5.6-fold lower abundance than sites with prior database annotations within a 95% confidence interval (Welch’s *t*-test, *P* = 7.2 × 10^−288^). The lower and the upper hinges of the boxes correspond to the 25th and 75th percentile, and the bar in the box to the median. The upper and lower whiskers extend from the largest and lowest values, respectively, but no further than 1.5× the IQR from the hinge. Points indicate outliers. (**C**) Left: Distribution of replicate coverage for regulated pY sites, separated by their membership in databased EGF response signatures. pY sites observed in five or all six out of six replicates of a condition are colored purple. Replicates observed in three or four out of six replicates are colored blue to highlight relative proportions of sites at higher risk of being missed in a three-replicate experiment. For reference, pY sites observed in only 1 or 2 replicates of a condition are colored white and gray. Right: Distribution of replicate coverage across regulated pY sites separated by their inclusion in database EGF signatures.

For a broader benchmark, we compared our EGF-regulated pY sites to a union signature assembled from four EGF-response resources: PhosphoSitePlus (Data ref: Hornbeck et al, 2012), PTMSigDB (Data ref: Krug et al, 2019; Data ref: Perturbation Site Database, 2018), Cell Signaling Technology, Inc. (CST) (Data ref: ErbB/HER Signaling Pathway, 2016), and WikiPathways (Data ref: Martens et al, 2021; Data ref: Kandasamy et al, 2010) EGFR signaling (WP437) databases. Inter-database agreement was low—only 18% of pY sites were shared between PhosphoSitePlus and PTMSigDB, and just 4% of EGF-annotated proteins were common to all four databases—reflecting different curation strategies across resources (Fig. EV5A,B). Combining the four resources yielded a union signature of 602 regulated pY sites on 439 proteins. R2HaPpY recovers 29% (172/602) of these union-annotated EGF-regulated sites in a single experiment, and 45% of our regulated pY sites map to proteins with annotated roles in the EGF response pathway (Fig. 5A; temporal responses of overlapping sites are shown in Fig. EV5C). The remaining regulated sites—those on proteins not previously annotated as EGF-responsive—are the focus of the next section.

### R2HaPpY captures novel, low-abundance EGF-regulated pY sites

Of the EGF-regulated pY sites identified by R2HaPpY, 80% were absent from both site-level resources, PhosphoSitePlus and PTMSigDB (Fig. 5A). Two factors account for this expanded detection: improved sensitivity for lower-abundance sites and the higher per-condition replication enabled by the cost and throughput of R2HaPpY.

R2HaPpY’s sensitivity to low-abundance sites is reflected in both intensity and detection consistency. Sites previously annotated in PhosphoSitePlus or PTMSigDB are >4-fold more abundant in our dataset than the novel sites we detect (Fig. 5B), indicating that the novel set sits below the effective detection threshold of prior methods. Consistent with their lower abundance, novel sites are detected less consistently across replicates: 65% of previously annotated sites are detected in all 6 replicates compared to 46% of novel sites (Fig. 5C). Because quantitative phosphosite measurements are known to carry higher technical variability than protein-level measurements (Hogrebe et al, 2018; Lawrence et al, 2016; Bekker-Jensen et al, 2020), replicate depth is essential for reliable regulation calls on low-abundance sites. A three-replicate design would have missed a substantial fraction of the novel set, given 42% (257) of novel sites were recovered in three to five replicates compared to 24% (45) of known sites.

### R2HaPpY identifies 70 pY sites absent from PhosphoSitePlus

Out of the novel EGF-regulated sites detected with R2HaPpY, 70 pY sites have not previously been reported at all in PhosphoSitePlus (Hornbeck et al, 2012), which currently contains 39,118 pY sites (Dataset EV3). To better understand why these sites have never been observed, we examined the peptides and found that a subset of pY sites occurred on peptides with N-terminal clipping and/or acetylation. These included LIMD1-pY4, TKT-pY4, NECAP-pY6, RAB14-pY6, STX12-pY9, PLCG2-pY13, and GRB10-pY15. These sites may have been missed in earlier studies due to database search parameters. We include annotated spectra from a subset of the novel pY sites to indicate confidence in the site assignments (Appendix Figure S18).

To investigate the biological relevance of these 70 novel pY sites, we mapped the sites onto their respective proteins and investigated them in the context of the four different EGF signaling databases described above. We found that 19 of the novel sites were on proteins previously implicated in EGF signaling response (Fig. EV5C). One novel pY site at residue Y15 on GRB10, an important scaffold protein involved in growth and proliferation (Deng et al, 2008), is the first evidence of a post-translational modification within the first 67 residues and may affect tetramerization and downstream signaling (Dong et al, 1998). Another novel EGF-regulated site, Y4 on transketolase protein TKT, could modulate nuclear localization (Qin et al, 2019), as this residue was shown to interact with EGFR, MAPK3, and the nuclear import machinery (Qin et al, 2019). Nuclear localization of TKT has been associated with high metastatic potential in hepatocellular carcinoma (Qin et al, 2019) and modulating this phosphosite could present a new therapeutic avenue. The other 51 novel pY sites were on proteins not previously annotated in EGF response signatures. Half of the remaining pY sites (26 of 51) were observed on proteins involved in endosomal regulation of EGFR (Appendix Text S1). To better understand the functional implications of the novel sites, we selected two sites in the endosomal sorting pathway and one outside for further analysis using published studies, structures, and amino acid conservation. We overview the endocytic proteins with novel sites and provide detailed analysis and description of RNF126 pY27, HGS pY197, and WEE1 kinase pS396/pY397 in the Appendix Text S1. Based on structural alignment (Krysztofinska et al, 2016; van Dijk et al, 2005) and prior functional studies (Smith et al, 2013), novel pY27 on RNF126 may participate in the recognition of ubiquitin linkage type on EGFR during its passage from early to late endocytosis. We additionally identified novel pY on Y197 of HGS/HRS, which likely reduces affinity for PI3P phospholipid head group in early endosomes, given its position in the FYVE domain. Outside of endocytic regulation, we identified novel dual phosphorylation of WEE1 S396 and Y397, in a kinase-specific yet understudied loop between D and E helices, offering a potential handle for WEE1-specific regulation.

Additional previously unseen pY sites on proteins not previously annotated in EGF response databases included several novel pY sites on proteins known to modify other proteins or participate in post-translational modification (PTM) crosstalk (Leutert et al, 2021; Swaney et al, 2013). These include pY117 on the E3 ubiquitin ligase RAD18, pY238 on STAMBPL1 (a zinc metalloprotease that specifically cleaves Lys-63-linked polyubiquitin chains), and the first documented pY site on the protein modifier SUMO1 (pY21). The involvement of these novel sites within EGF signaling is currently speculative but illustrates how a deep systems-level analysis of pY signaling using our method can uncover new, potentially functionally relevant pY sites and can drive hypothesis generation for further experiments.

### Uncovering the phosphotyrosine temporal response to EGF

**Figure 6:**
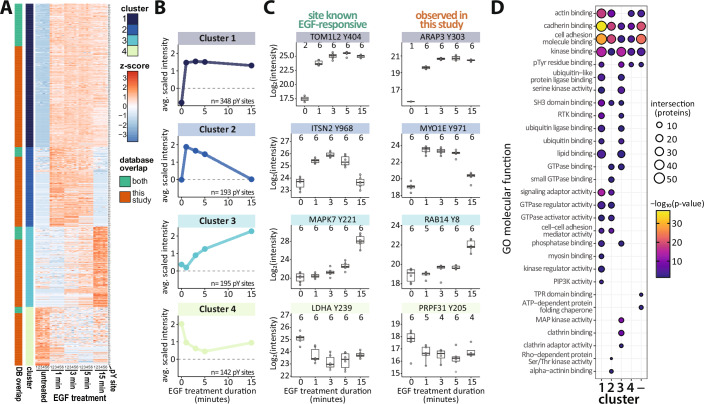
Temporal response of EGF-regulated pY sites and protein functional enrichments. (**A**) pY site responses fall into 4 distinct clusters. Clusters were assigned by the fuzzy c-means soft clustering method. Only pY sites with a significant abundance change relative to untreated were included in the heatmap (878 pY sites). Heatmap shows scaled intensities of individual pY sites (rows) per biological replicate and EGF treatment duration (columns). Rows are organized by cluster and then by overlap with prior database annotation. Rows are annotated to indicate overlap with prior database annotation and cluster membership (left). Database overlap annotation is green if the pY site has been annotated in either PhosphoSitePlus or PTMSigDB EGF response signatures and orange if the site is not in either database. Cluster coloring proceeds from dark blue to light green for clusters 1 through 4. (**B**) The line plot indicates average scaled intensities of all pY sites per cluster across EGF treatment durations. Numbers of pY sites belonging to each cluster are listed at the bottom of each plot. (**C**) Examples of individual pY sites showing temporal responses to EGF treatment for each cluster. The left column shows pY sites previously annotated as EGF responsive by PhosphoSitePlus or PTMSigDB. The right column shows pY sites observed in this study without prior annotation in either database. Points indicate individual replicate measurements, and boxplots summarize the distribution of measurements per treatment duration. The lower and the upper hinges of the boxes correspond to the 25th and 75th percentiles, and the bar in the box to the median. The upper and lower whiskers extend from the largest and lowest values, respectively, but no further than 1.5× the IQR from the hinge. The number of measured replicates is indicated at the top of the plots. (**D**) Gene ontology molecular function enrichment results of proteins containing pY sites within each cluster or otherwise not regulated (x-axis dash). Enrichment scores were determined with the gprofiler2 tool; significance was tested with Fisher’s one-tailed test (cumulative hypergeometric probability test). Significant molecular functions are listed on the y-axis, and the cluster is indicated on the x-axis. The size of points indicates the number of proteins within each cluster that match the molecular function term. The color of points indicates the significance of the functional enrichment, where yellow is more significant and blue is less significant. Absence of a point indicates no significant enrichment.

We examined the temporal response of EGF-regulated pY sites by fuzzy c-means clustering of site intensities across time points, with k = 4 clusters selected by the elbow method (see Methods). The four clusters corresponded to: rapid and sustained increase from 1 min (cluster 1); transient increase peaking at 1–3 min followed by decay after 5 min (cluster 2); gradual increase across the full time course (cluster 3); and steady decrease within the first 5 min (cluster 4) (Fig. 6A,B; Appendix Fig. S7A). Cluster 1 contained 60% (103/172) of previously annotated EGF-responsive pY sites and comprised the highest intensity sites in the dataset (Appendix Fig. S7B,C). In contrast, clusters 2, 3, and 4 were enriched for lower abundance sites, most of which are novel to this study (Fig. 6C), indicating that prior EGF pY studies have predominantly resolved the rapid and sustained response while leaving other temporal patterns under-characterized.

To understand the functions of proteins within each EGF response cluster, we performed gene ontology enrichment analysis, looking at cell component, biological process, and molecular function (Fig. 6D; Appendix Fig. S8A,B). As expected, proteins involved in cell structure modulation were enriched among all clusters but were most significant in clusters 1 and 2, which reached maximal pY site intensity at one minute, consistent with cell morphology changes in early EGF signaling. Additionally, proteins involved in GTPase signaling were enriched in clusters 1 and 2, consistent with the transient proximity of GTPase activators to GTPases during the internalization of the EGFR-GRB2-SOS complex one to five minutes after EGF stimulation (Surve et al, 2021; Pinilla-Macua et al, 2016) (Fig. 6D; Appendix Fig. S8B,C). We also observed that regulated pY sites on serine-threonine kinases are enriched in cluster 1 and 3 response types (Fig. 6D; Appendix Fig. S8C), suggesting that pY activation of these kinases either reaches maximal signaling within 1 min and is sustained or gradually increases through 15 min. Conversely, regulated pY sites on MAPKs were primarily enriched in cluster 3, with a gradual increase across the time course.

In the enrichment analyses, we also included pY sites which were not regulated in response to EGF and stayed the same throughout the time course. These non-EGF-regulated pY sites were enriched in multiple molecular functions, biological processes, and cellular components; however, these sites were uniquely enriched for nucleocytoplasmic transport (Fig. 6D; Appendix Fig. S8A,B). As translocation of proteins is a key part of signaling pathways and often facilitated by pY signaling, this enrichment suggests R2HaPpY detects a broad range of pY sites that could potentially be regulated at longer time points after EGF stimulation, or which could be regulated in response to alternative stimulations.

Together, the clustering and enrichment analyses reveal temporal heterogeneity in the EGF pY response that prior, shallower datasets could not resolve. While sustained, high-intensity sites from the single dominant cluster 1 were well captured by earlier methods, the transient, gradually increasing, and decreasing response patterns are largely populated by sites newly accessible through R2HaPpY. To enable exploration of temporal profiles across all 1651 quantified pY sites, we developed a web-based resource at https://r2happy.gs.washington.edu/.

### Deep pY time course data reveal regulation beyond what canonical pathway maps predict

Deeply resolved pY time courses, now practical with R2HaPpY, allow systematic comparison of site-level dynamics against existing pathway annotations—an analysis previously tractable mainly for serine/threonine phosphoproteomes (Olsen et al, 2006; Leutert et al, 2023). We asked whether a protein’s temporal response profile is predicted by its position in a canonical linear pathway map. We assigned each protein in the human EGF/EGFR WikiPathway (WP437) a node depth by tallying edges from EGFR (Dataset EV4) and ordered all regulated pY sites by this depth alongside their cluster assignment and scaled intensity at each time point (Appendix Fig. S9A). If WP437 captured the regulatory logic of the network, proteins near EGFR would predominantly show rapid (cluster 1 or 2) responses, and those further downstream would show slower (cluster 3) accumulation. Instead, we found no significant relationship between node depth and temporal cluster (Welch’s *t*-test, *P* > 0.05; Appendix Fig. S9A,B).

This result is consistent with prior systems-level studies reporting that signaling networks deviate substantially from their canonical linear representations (Invergo and Beltrao, 2018; Breitkreutz et al, 2010; Abd-Rabbo and Michnick, 2017; Levy et al, 2010; Köksal et al, 2018). It is not a new biological conclusion; rather, it demonstrates that a pY dataset of this depth and temporal resolution can uncover specific site regulation where the canonical map is incomplete. For example, Reddy et al previously reported rapid EGF-induced tyrosine phosphorylation of tensin, cortactin, and plakophilin despite the absence of direct EGFR interactions in pathway annotations (Reddy et al, 2016). We recapitulate these responses and additionally detect lower-abundance sites on the same proteins, illustrating the gain in coverage.

Two further patterns are worth noting. First, among the 15 EGF-regulated pY sites detected across eleven MAPKs and two MAPKAPKs (Appendix Fig. S10), nine occur at known activation loop positions and show the expected gradual accumulation through 15 min. In contrast, MAPKAPK2 pY228 and MAPKAPK3 pY207—both positioned between the T222/S272 and T201/S251 activating sites, respectively (Ben-Levy et al, 1995)—peak within 1 min of stimulation and decline thereafter. MAPKAPK2 pY228 has not been previously observed, and MAPKAPK3 pY207 has no assigned function; both warrant dedicated follow-up. Second, although the scaffold protein IQGAP1, known to bind B-Raf, MEK, and ERK (Roberts and Der, 2007), lies at node depth 5 in WP437 (Martens et al, 2021), we observed rapid regulation of two sites within 1 min: pY1510 (known to be phosphorylated by MET and c-Src (Hedman et al, 2020)) decreases, while pY17 increases. These responses are unexpected for a protein positioned downstream in the canonical map and are consistent with reports that IQGAP1 associates directly with EGFR during stimulation (McNulty et al, 2011).

Several additional proteins annotated at intermediate node depths (2–6), including PTPN11, SOS2, STAT3, GAB1, GAB2, PLCG1, PIK3C2B, PTK2B, and PRKCD, also carry at least one pY site that reaches maximal intensity within 1 min (Appendix Fig. S9A). Conversely, several branches of the network do behave as the canonical map predicts: pY sites on EGFR-proximal proteins (VAV2, CBL, CBLB, NCK1, JAK1, BCAR1, and EPS8) showed primarily cluster 1, 2, or 4 intensity profiles indicative of immediate phosphorylation change and were concentrated to early pathway depths of 1–3. Similarly, pY sites on MAP kinases showed primarily cluster 3 temporal profiles consistent with their deeper network position (Appendix Fig. S9C). Together, this suggests that current signaling maps are valuable references to explain a portion of interactions; however, with sensitive pY profiling, additional network dependencies and connectedness can be revealed.

### Differential regulation of EGF-responsive pY sites on the same protein

EGF-responsive pY sites on the same protein often fall into different temporal clusters. Among proteins annotated in the EGF/EGFR signaling pathway (WP437), 78% of regulated pY sites showed a temporal profile distinct from at least one other pY site on the same protein (Appendix Fig. S9A). A comparable pattern was reported for the global phosphoproteome by Olsen et al, who found 77% of EGF-responsive phosphoproteins carried at least one additional phosphosite with different temporal behavior (Olsen et al, 2006). Our dataset extends this observation to pY-specific resolution at substantially greater depth: 878 EGF-regulated pY sites here compared to 53 in the earlier study.

**Figure 7:**
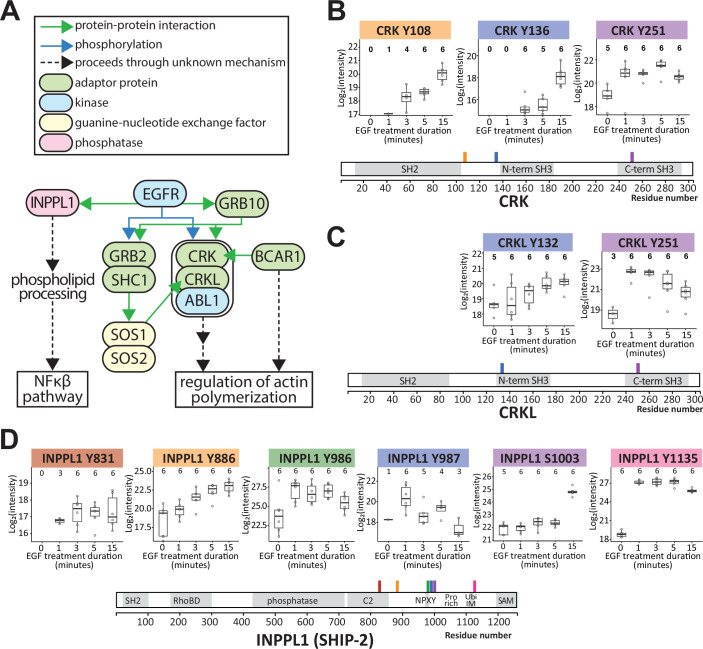
Examples of different pY site temporal profiles within proteins. (**A**) Schematic of a subset of EGF/EGFR signaling adapted from WikiPathways #437. (**B**) Boxplots indicate the distribution of pY site intensity at each EGF treatment duration for the protein CRK. Points indicate individual measurements per replicate. The total number of replicates for which a pY site was measured per treatment duration is indicated near the top of each plot. The title of each plot indicates the protein and pY site location. The background color of the plot title corresponds to the location of the pY site along the schematic of protein domain boundaries indicated. Domain boundaries were adapted from PhosphoSitePlus. The lower and the upper hinges of the boxes correspond to the 25th and 75th percentiles, and the bar in the box to the median. The upper and lower whiskers extend from the largest and lowest values, respectively, but no further than 1.5× the IQR from the hinge. (**C**) Boxplots indicate the distribution of pY site intensity at each EGF treatment duration for the protein CRKL. All other plotting information is the same as for panel (**B**). (**D**) Boxplots indicate the distribution of pY site intensity at each EGF treatment duration for the protein INPPL1 (SHIP-2). Schematic of domain boundaries was adapted from both PhosphoSitePlus and Le Coq et al (2021). All other plotting information is the same as for panels (**B**, **C**). “RhoBD” indicates a putative Rho binding domain. The “phosphatase” domain indicates the 5-inositol phosphatase domain. The “C2” domain indicates the C2 calcium and lipid-binding domain. “NPXY” indicates the conserved amino acid motif in which the Y is phosphorylated and is commonly involved in protein trafficking. “Pro-rich” indicates the proline-rich region spanning amino acids 930–1110. “SAM” indicates the sterile alpha motif. Note: INPPL1 pS1003 was from the global phosphoproteome (Fe-IMAC) dataset rather than the pY-specific enrichment. Boxplots are the same as defined in panel (**B**).

The adapter protein CRK illustrates this pattern (Fig. 7A,B). pY251, located in the C-terminal SH3 domain and known to engage ABL kinase (Sriram et al, 2015), reached maximal intensity within 1 min. Two sites in the flexible linker between the SH2 and N-terminal SH3 domains, pY108 and pY136, instead increased gradually without plateauing by 15 min. CRK pY136 has been reported to decrease with gefitinib treatment (Moritz et al, 2010), indicating that sites in this dataset include targets modulated by clinically used EGFR inhibitors.

The adapter protein CRKL shows an analogous pattern. pY132 and pY251 both increase after EGF stimulation but with different kinetics: pY251 peaks at 1 min while pY132 plateaus by 15 min (Fig. 7C). PhosphoSitePlus annotates pY132 as an EGFR substrate and pY251 as a ZAP70 substrate in T-cell signaling (Hornbeck et al, 2012). The diversity in temporal signaling profiles we observe is consistent with distinct upstream kinase inputs.

The inositol phosphatase SHIP2/INPPL1, recruited to EGFR early in signaling to downregulate the PI3K pathway (Pesesse et al, 2001), carries four EGF-responsive pY sites with divergent temporal profiles (Fig. 7D). The two sites reported as EGFR substrates, pY986 and pY1135—located in the proline-rich region on the NPXY motif and adjacent to the ubiquitin-interacting motif (Le Coq et al, 2021), respectively, reached maximal intensity at 1 min. Two additional sites, pY886 and pY831, located closer to the C2 lipid-binding domain, showed slower increases through 15 min. INPPL1 pS1003, shown alongside in Fig. 7D, is from the parallel global phosphoproteome dataset; we include it to illustrate how pY enrichment and global phosphoproteomics on the same sample together resolve regulatory PTMs that neither approach captures alone.

Across the full regulated dataset, 66% (100/152) of proteins with more than one regulated pY site carry pY sites with distinct temporal profiles. This supports the recommendation that post-translational modifications and their regulation should be reported at the site level and not aggregated to the protein level (Olsen et al, 2006; Krug et al, 2019).

### Concluding remarks

R2HaPpY brings phosphotyrosine enrichment to a scale and cost at which large-scale, replicated tyrosine phosphoproteomics becomes broadly practical for the first time. R2HaPpY couples a single-step preparation of HaloTag-fused Src SH2 superbinder beads with an automated 96-well pY peptide enrichment. The method is sensitive at low input and reproducible, while reducing reagent cost ~20-fold and hands-on preparation time from days down to 1 h relative to existing antibody- and superbinder-based enrichment approaches. Applied to EGF-stimulated HeLa cells, R2HaPpY quantified 1651 unique pY sites and resolved temporal heterogeneity and low-abundance regulation that prior methods have not captured. By removing the principal practical barriers to large-scale pY profiling, R2HaPpY enables tyrosine phosphoproteomics to be addressed at the cohort and timecourse scales that have transformed serine/threonine phosphoproteomics over the past decade. With this scale now accessible, pY signaling can be routinely integrated into multiomic and clinical-scale studies.

## Methods

### Preparation of Halo-sSrc beads

#### Construct design

A HaloTag^TM^ was fused N-terminal to the Src SH2 superbinder (sSrc) in the pET28a(+) *Escherichia coli* expression vector. The sSrc protein contains mutations T8V, C10A, and K15L (Kaneko et al, 2012). A Tobacco Etch Virus (TEV) protease cleavage site was included between the HaloTag and sSrc SH2 domain, and a hexahistidine tag was included at the N-terminus. Each component is connected by short serine-glycine linkers. Sequences and plasmid maps for both the full plasmid and open reading frame are shown in Appendix Figs. S16 and S17; Dataset EV5.

#### Halo-TEV-sSrc expression

*E. coli* culture (BL21 (DE3) pET28a(+)::His₆-Halo-TEV-sSrc Kan^R^) was grown in Luria Broth with kanamycin (50 µg/mL) to an OD_600_ of 0.6–0.8, then induced with 0.1 mM isopropyl β-D-1-thiogalactopyranoside (IPTG) for 3–4 h at 37 °C with 225 rpm shaking. Cells were pelleted, washed in phosphate-buffered saline (PBS), aliquoted into 50 mL culture equivalents, and stored at −80 °C.

#### Halo-TEV-sSrc lysis and conjugation

Frozen *E. coli* cell pellets containing Halo-TEV-sSrc were resuspended in 1× PBS pH 7.4 supplemented with 0.1 mg/mL lysozyme and 12–25 U/mL benzonase nuclease at a ratio of 4 mL buffer to 50 mL culture equivalent of cell pellet. Cell suspension was rocked at 4 °C for 5–10 min prior to sonication using 11–12-watt output in six intervals of 15–30 s with equal rests on ice. Lysate was clarified at 21,000×*g* for 15 min at 4 °C.

Prior to conjugation, Magne® HaloTag® beads (Promega) were equilibrated by washing three times in ultrapure (18 MΩ·cm) water followed by three washes in 1× PBS, pH 7.4.

Clarified *E. coli* supernatant was transferred to equilibrated Magne® HaloTag® beads and rocked end-over-end for 45 min at 4 °C. Ratio of *E. coli* lysate to magnetic beads was 4 mL clarified lysate per 1 mL of 20% magnetic bead slurry.

Unbound lysate was discarded, and magnetic beads were washed six times in 1–2 column volumes (CVs) of 1× PBS, pH 7.4. Prior to enrichment, beads were washed three times in 50 mM Tris, 50 mM NaCl, pH 8.0. Beads were stored at 4 °C for up to 4 months in 1× PBS pH 7.4 or 50 mM Tris, 50 mM NaCl, pH 8.0. To enrich pY peptides, 40 µL of 20% bead slurry was used per sample.

#### Conjugation assessment

Successful conjugation of Halo-TEV-sSrc protein to the Magne® HaloTag® beads was confirmed by TEV protease digest. SDS PAGE analysis of the digest reaction identified the presence of the ~14 kDa sSrc SH2 domain. Briefly, a 50 µL reaction consisting of 10 µL conjugated bead slurry, 0.2 µg TEV protease, 50 mM dithiothreitol (DTT), and 10 mM ethylenediaminetetraacetic acid (EDTA) was incubated 16–24 h at room temperature with mixing. Half of the digest (25 µL) was analyzed by reducing and denaturing SDS PAGE using a 4–12% BisTris NuPAGE Bolt gel run at 130 V for 80 min in 1× MOPS/SDS buffer alongside a PAGERuler Plus size marker (Appendix Fig. S11). A high intensity 14 kDa band indicated ample sSrc was cleaved away from the HaloTag, which remained covalently attached to beads.

### Preparation of NHS-sSrc beads

The His_6_-sSrc-SH2 construct was purified and conjugated to magnetic beads as described previously (Chang et al, 2023).

### HeLa culture, lysis, and quantification

HeLa S3 cells were cultured at 37 °C and 5% CO_2_ in Dulbecco’s modified Eagle’s medium (DMEM) supplemented with 4.5 g/L glucose, l-glutamine, 10% fetal bovine serum (FBS), and 0.5% streptomycin/penicillin. Cells tested negative for mycoplasma contamination. To generate bulk phosphopeptides for method comparisons, cells were grown to an average of 80% confluency before harvesting. For pervanadate treatment, cells were incubated in serum-free medium for 6 h prior to the addition of 1 mM pervanadate for 15 min, followed by the addition of 10% FBS for 15 min. For epidermal growth factor (EGF) stimulation, cells were starved for 20 h in DMEM containing 0.7% FBS before the media was exchanged with DMEM containing 0.7% FBS and 160 ng/mL EGF. Cells were harvested at 1 min, 3 min, 5 min, and 15 min after stimulation. Control plates received no EGF and were directly harvested. To harvest, cells were rinsed three times quickly with ice-cold PBS, then flash-frozen in liquid nitrogen prior to storage at −80 °C. Cells were lysed by scraping in 1 mL denaturing lysis buffer (8 M urea, 50 mM HEPES, 75 mM NaCl, pH 8.0) per 15 cm plate. Lysate was sonicated twice for 8 s at 11–12-watt output on ice with intermittent rests. Lysate was clarified by centrifugation at 21,100×*g* and 12 °C for 10 min. Soluble lysate was transferred to fresh tubes and quantified by bicinchoninic acid (BCA) assay (Pierce). We chose a high concentration of EGF to investigate receptor signaling and endocytic sorting. Users applying R2HaPpY to study dose-dependent biology should titrate treatment concentration to their specific experimental question.

### Yeast strains, growth, lysis, and quantification

The yeast strain expressing beta-estradiol-inducible vSrc kinase was a gift from the Landry lab (Bradley et al, 2024). Cells were grown overnight at 30 °C with 250 rpm shaking in yeast extract peptone dextrose (YEPD) media. Cells were diluted to an OD_600_ of 0.1 in synthetic complete media (1× yeast nitrogen base, 2% glucose, all amino acids) containing 100 nM beta-estradiol prepared in DMSO) and grown to OD_600_ of 0.8–1.0. Cells were pelleted, washed with 1× ice-cold PBS, flash-frozen in liquid nitrogen, and stored at −80 °C. Cell lysis was carried out in 8 M urea, 150 mM Tris, pH 8.2, by bead beating four times with 1 min intermittent rests on ice. Lysate was collected, then clarified by centrifugation at 4 °C and 21,000×*g* for 10 min. Protein concentration was quantified by BCA assay.

### Lysate reduction and alkylation

Proteins were reduced with 5 mM DTT at 55 °C for 30 min, 1000 rpm mixing. Lysate was cooled to room temperature prior to alkylation with 15 mM iodoacetamide at 24 °C and mixing at 700 rpm in the dark. Alkylation was quenched with an additional 5 mM DTT and 700 rpm mixing at 24 °C in the dark for 30 min.

### Digestion and desalting

Reduced and alkylated protein lysate was diluted five-fold in 50 mM ammonium bicarbonate, pH 8.0, containing a 1:100 w/w ratio of trypsin (Promega). Proteins were digested for 15 h at 37 °C while shaking at 200 rpm. Digests were acidified to a final pH <2 with trifluoroacetic acid (TFA). Precipitates were removed by centrifugation at 7000×*g* for 10 min at 24 °C. Peptides were desalted on a Waters C18 Sep-Pak 100 mg capacity. Briefly, cartridges were equilibrated with 1 CV methanol (MeOH), 3 CVs acetonitrile (ACN), 1 CV 70% ACN 0.25% acetic acid (AA), 1 CV 40% ACN, 0.5% AA, and 3 CVs of 0.1% TFA. Peptides were loaded and flow-through was reloaded. Cartridges were washed with 3 CVs 0.1% TFA and 1 CV 0.5% AA prior to elution of peptides with 0.8 mL of 40% ACN, 0.25% AA, followed by 0.6 mL 70% ACN, 0.5% AA. Eluates were mixed and aliquoted for total proteome measurement, global phosphopeptide enrichment, and pY peptide enrichment prior to drying by vacuum centrifugation.

### R2P2 for global phosphopeptide enrichment

Enrichment was performed on the KingFisher flex as previously described (Leutert et al, 2019). Specifically, each 250-µg aliquot of dried peptides was resuspended in 900 µL 80% ACN, 0.1% TFA. Precipitates were removed by centrifugation for 10 min at 21,000×*g* and 4 °C. Clarified peptides were added to a deep 96-well plate, leaving behind 50 µL to avoid transfer of precipitate. Washes were prepared by filling three shallow 96-well plates with 150 µL of 80% ACN, 0.1% TFA. The bead plate was prepared by adding 70 µL of 5% Fe^3+^ NTA magnetic beads in 80% ACN, 0.1% TFA. A tip comb was nested in a shallow well plate. The R2P2 global phosphopeptide enrichment protocol (10.17504/protocols.io.q26g7n3y9lwz/v1) was initiated on the KingFisher Flex. With ~11 min remaining in the protocol, the elution plate was prepared by adding 75 µL of 2.5% ammonia, 50% ACN to wells and added during the programmed pause. During the KingFisher method, two layers of C8 extraction disks were nested in a P200 tip and equilibrated with the following solutions using centrifugation cycles of 2 min at 300×*g*: 60 µL of 100% MeOH, 60 µL of 100% ACN, 60 µL of 70% ACN containing 0.25% AA. Eluates were acidified with 45 µL of 10% formic acid (FA), 75% ACN immediately upon method completion. Eluates were filtered through two layers of C8 material equilibrated as described above. Eluates were collected in MS vials along with a subsequent wash of 60 µL 70% ACN, 0.25% AA. Filtered eluates were dried by vacuum centrifugation and stored at −20 °C until MS measurement.

### R2HaPpY with R2P2 cleanup

Dried peptides ranging in amounts from 0.25 to 4 mg according to experiment, were resuspended in 900 µL of 50 mM Tris, 50 mM NaCl, pH 8.0 by vortexing and sonication. Precipitate was pelleted by centrifugation at ≥10,000×*g* for 10–15 min at 4 °C. Clarified peptide solution was added to the deep well KingFisher “Binding Plate” leaving behind 50 µL to avoid adding precipitate. Halo-TEV-sSrc beads were equilibrated into fresh 50 mM Tris, 50 mM NaCl, pH 8.0 by washing twice and resuspended at a 20% bead slurry concentration. A total of 40 µL of bead slurry was mixed with each peptide sample. The tip plate was prepared by nesting a deep well tip comb in a shallow well plate. Three wash plates were prepared by adding 850 µL of 50 mM Tris, 50 mM NaCl, pH 8.0 per well. A fourth wash plate was prepared by adding 850 µL of HPLC-grade water per well. Two elution plates were prepared by adding 100 µL of 0.5% TFA per well to the first and 120 µL of 1% TFA, 60% ACN per well to the second plate. The R2HaPpY KingFisher Flex BindIt protocol (10.17504/protocols.io.q26g7n3y9lwz/v1) was started with all plates except elution 2, which was prepared and added at the pause in the last 15 min. Upon completion of enrichment, elution 2 was combined with elution 1.

Pooled eluates were transferred to microfuge tubes and diluted to reach 75% ACN in a total volume of 900 µL. Any precipitates or residual beads were pelleted by centrifugation for 4 min at 16,000×*g*. Supernatant was transferred to a deep well plate for additional cleanup using the R2P2 global phosphopeptide enrichment protocol described above, with the following two differences.

First, the deep well plate containing the clarified pY peptide eluate at 75% ACN served as the peptide binding plate. Second, the bead plate contained 80 µL of 5% Fe-NTA IMAC bead slurry in 80% ACN, 0.1% TFA. All other steps described above were the same. Dried pY peptide samples were resuspended in 12 µL of 4% ACN, 3% FA in water for mass spectrometry measurement.

### Mass spectrometry measurement

All measurements were performed on an Exploris 480. The EGF stimulation experiment used an Easy nLC II, while all other experiments used an Easy nLC 1200. Peptides were loaded onto a 100 µm ID x 3 cm silica trap column packed with Reprosil C18 3 µm beads (Dr. Maisch GmbH). Peptides were separated along a 100 µm ID x 30 cm analytical column packed with Reprosil C18 3 µm beads for 2 cm in the tip, followed by 28 cm of Reprosil C18 1.9 µm beads (Dr. Maisch GmbH). The analytical column was housed in a column heater, set to 50 °C and a voltage was applied between the trap and analytical columns. All data were acquired in data-dependent acquisition (DDA) mode on the Thermo Exploris 480 Orbitrap mass spectrometer.

#### Tyrosine phosphoproteome measurements

Peptides were eluted using a gradient of 6.4 to 32% ACN in 66 min followed by 32% to 48% ACN for 7 min, followed by 76% ACN wash for 6 min and 2.4% ACN equilibration for 11 min using a flow rate of 300–400 nL/min, where solvent A is HPLC-grade 0.1% FA and solvent B is HPLC-grade 80% ACN, 0.1% FA.

Full MS scans were acquired from 375 to 1500 m/z at 120,000 resolution with a fill target of 3e6 ions and maximum injection time set to automatic. The most abundant ions on the full MS scan were selected for fragmentation using a 1.6 m/z precursor isolation window and beam-type collisional-activation dissociation (HCD) with 30% normalized collision energy for a cycle time of 3 s. Precursor intensity threshold was set at 2.5e4, and only precursors with charge states determined to be 2–6 were fragmented. MS/MS spectra were collected at 45,000 resolution with a fill target of 2e5 ions and maximum injection time of 86 ms.

For high phosphotyrosine-containing samples such as pervanadate-treated HeLa and Yeast vSrc, precursors were dynamically excluded from selection for 45 s. For low phosphotyrosine-containing samples, such as untreated or EGF-stimulated HeLa cells, precursors were fragmented up to twice within 20 s prior to exclusion for 20 s.

#### Global phosphoproteome measurements

Analysis of the global phosphoproteome was the same as the tyrosine phosphoproteome except with different LC gradient and dynamic exclusion settings. Specifically, phosphopeptides were separated by increasing amounts of ACN in 0.1% FA over a 73 min gradient, specifically ranging from 3.2 to 6.4% ACN in 3 min, 6.4 to 25.6% ACN in 63 min, 25.6 to 48% ACN in 7 min, followed by 76% ACN for 6 min and 2.4% ACN for 11 min using a flow rate of 400 nL/min. Fragmented precursors were dynamically excluded from selection for 45 s.

#### Total proteome measurements

Dried 10 µg aliquots of tryptic peptides representing the total proteome of HeLa cells in the EGF stimulation experiment were resuspended in 3% ACN, 4% FA for measurement of 0.5 µg by data-dependent acquisition. Peptides were separated by a 43-min gradient ranging from 6.4 to 36% acetonitrile in 0.1% FA using a flow rate of 350 nL/min. Full MS scans were acquired from 350 to 1500 m/z at 120,000 resolution with a fill target of 3e6 ions and maximum injection time set to automatic. The 15 most abundant ions on the full MS scan were selected for fragmentation using 2 m/z precursor isolation windows and beam-type collisional-activation dissociation (HCD) with 30% normalized collision energy. MS/MS spectra were collected at 15,000 resolution with the standard fill target and maximum injection time fixed to 40 ms. Fragmented precursors were dynamically excluded from selection for 30 s.

### Data analysis

#### All datasets

MS/MS spectra were searched with Comet (release 2019.01.2) (Eng et al, 2013) against the human proteome downloaded from Uniprot on March 21, 2024, including only reviewed entries and excluding isoforms (20,418 entries). The yeast samples were searched against the *S. cerevisiae* proteome downloaded from Uniprot on February 19, 2021, with the vSrc kinase sequence appended. Peptides were searched with trypsin digestion, allowing up to two missed cleavages. The precursor *m/z* tolerance was set to 20 ppm. The fragment *m/z* mass tolerance was set to 0.02 Da. Constant modification of cysteine carbamidomethylation (57.02146372118 Da) and variable modification of methionine oxidation (15.9949146202 Da, max 2), and N-terminal acetylation (42.01056468472, max 1) were used for all searches. An additional variable modification of serine, threonine, and tyrosine phosphorylation (79.966331 Da, max 3) was used for global and tyrosine-specific phosphopeptide samples. Search results were filtered to a 1% FDR at the peptide-spectrum match (PSM) level using Percolator (Käll et al, 2007). Phosphorylation sites were localized using an in-house implementation of the Ascore algorithm (Beausoleil et al, 2006; Barente and Villén, 2023). Phosphorylation sites with an Ascore ≥13 (*P* ≤ 0.05) were considered confidently localized. Peptides were quantified using an in-house implementation of the moFF algorithm (Argentini et al, 2016) to extract MS1 peak maximum intensities.

### Bioinformatic analyses

Bioinformatic analysis was performed using R (https://www.r-project.org/). Quantitative values were consolidated to the maximum intensity peptide spectral match per precursor ion (peptide sequence with charge state). For total proteome analysis, precursor ion intensities were summed to the peptide level or to the protein level. For phosphorylation site-level analysis, all peptides containing the confidently localized phosphosite were summed up. For phosphosite isoform analysis, all peptides containing the same one to three co-occurring and confidently localized phosphosites were summed up. Intensity distributions of peptides, proteins, phosphosites, or phosphoisoforms were median-normalized across conditions for each experiment.

For all boxplots, the lower and upper hinges of the boxes correspond to the 25th and 75th percentile, and the bar in the box to the median. The upper and lower whiskers extend from the highest and lowest values, respectively, but no further than 1.5× the IQR from the hinge.

All correlation calculations utilize Pearson’s method.

### Bioinformatic analysis of EGF signaling response

#### Completeness filtering and imputation

For the EGF stimulation data, pY sites were required to be observed in at least three of six biological replicates of at least one of five conditions.

Imputation was applied to missing replicates prior to differential abundance testing to capture pY sites that were completely undetected in one condition yet confidently measured in another. For sites missing in all biological replicates of a condition, log_2_-transformed intensities were imputed by sampling a random normal distribution centered at 10 with a standard deviation of 1. The intensity distribution was centered at 10 to represent intensities slightly below the limit of detection, given that the lowest observed log_2_-transformed intensity was 11.02 and the lowest 1% of log_2_-transformed intensities reached 15.02. Values between 8 and 12 were assigned 95% of the time. A standard deviation of 1 was chosen to provide a slightly greater variation than the average observed standard deviation of 0.63 to avoid inflated significance values and to represent higher variability inherent to lower abundance measurements. For sites detected in at least one replication of a condition, missing observations were imputed by sampling a randomized normal distribution, centered at the average intensity of measured replicates, with a standard deviation set to the global average standard deviation of 0.63.

Missing observations were also imputed prior to temporal response profiling by fuzzy c-means clustering (Fig. 6A,B), except without sampling from a randomized normal distribution. Instead, missing observations from all replicates of a condition received a log_2_-transformed intensity of 10, while missing observations from five or less replicates received a log_2_-transformed intensity set to the average intensity of the measured replicates.

By contrast, plotting of measured site intensities, such as in Figs. 5, 6C, and 7, only showed measured observations, excluding any imputed intensities.

#### Differential abundance testing

The calculation of individual *p* values used Welch’s two-sided *t*-test of biological replicates, using the untreated condition as control and applying the Benjamini–Hochberg procedure for multiple testing correction. Phosphosites were considered differentially abundant if the absolute fold-change was greater than 2 and the adjusted *p* value less than 0.05. The protti R package (Quast et al, 2022) version 0.8.0 was used for the calculation of differential abundance.

#### Database annotation analysis

For comparison of EGF-regulated pY sites in this study to database EGF response signatures, two databases were downloaded. The first database was retrieved from the Site Search tool at PhosphoSitePlus.org (Data ref: Hornbeck et al, 2012) on December 19, 2024, specifying treatment as EGF. The second database was downloaded from PTMSigDB (Data ref: Krug et al, 2019) v2.0.0 on September 3, 2024, for the semi-automatically curated EGFR1 perturbation dataset (Data ref: Perturbation Site Database, 2018). Regulated pY sites identified in this study were joined to both databases by UniProt ID, gene name, and pY site location within the full protein.

#### Clustering with temporal response profiling

Clustering was performed by fuzzy c-means soft clustering, specifically using the fcm function from the e1071 R package (https://rdocumentation.org/packages/e1071/versions/1.7-16). Only pY sites measured in at least 50% of replicates of one time point and determined to be significantly regulated with an absolute log_2_(fold-change) greater than one and adjusted *p* value less than 0.05 were included in clustering analysis. Imputation of missing replicates was performed as described above. pY site intensities were scaled prior to clustering. Clusters were assigned by maximum correlation to the overall cluster trend. The total number of clusters representing the distinct temporal response profiles were chosen based on the k-means grouping method, coupled with manual inspection for the minimal number of clusters showing distinct trends. The cluster determination by k-means was performed according to the elbow method by choosing the number of clusters at which the rate of increase in the amount of variation explained by adding another cluster drastically slowed (according to the sum of squares divided by the total sum of squares ratio calculated by the ‘kmeans’ function in the stats package in R (https://rdocumentation.org/packages/stats/versions/3.6.2)).

#### Gene ontology profiling

Unique proteins containing at least one regulated pY site were subset according to cluster and analyzed with gprofiler2 using the R package. To avoid overly broad functional enrichments, gene ontology terms were filtered for only terms containing 1000 or less children terms. Significant enrichment terms were then manually curated to avoid semantic redundancies.

#### Evidence distributions in PhosphoSitePlus

Phosphorylation site records were obtained from PhosphoSitePlus (accessed 31 October 2024; Data ref: Hornbeck et al, 2015), filtered for *Homo sapiens*, and analysed separately by residue type (phosphoserine and phosphothreonine [pSer/pThr] versus phosphotyrosine [pTyr]). Figure 1A reports the number of sites with three or more mass spectrometry (MS) observations, computed separately for (i) all MS sources combined, including both external studies and records from Cell Signaling Technology, Inc. (CST)); and (ii) external non-CST studies only, irrespective of whether CST evidence was also present. For each residue type, the percentage written in text indicates the fraction of phosphosites supported by at least three external studies, relative to phosphosites supported by any three records regardless of source.

### Survey of public phosphoproteomics datasets

Public phosphoproteomics submissions were retrieved from the ProteomeXchange/PRIDE repository via its JSON API using two independent queries: one for global phosphoproteomics and one for phosphotyrosine (pY) enrichment. Hits were ranked in descending order by the number of raw mass spectrometry files associated with each submission, and the top 38 entries in each category were carried forward for manual inspection; the remaining entries in the hit lists were not annotated. For each inspected submission we recorded the number of samples enriched by cross-referencing the raw file list against the methods section of the associated publication (where available), classified the study as biological (applied phosphoproteomics), methods (methods-development), or reanalysis (reprocessing of previously deposited data), and flagged whether any submitting author was an original developer of a phospho-enrichment method (e.g., EasyPhos, Src SH2 Superbinder-based enrichment, R2P2). Annotations for both categories were merged in R into a single table (Dataset EV1) with a Column_Descriptions sheet documenting each field. For the dot plot in Fig. 1B, entries classified as methods or reanalysis were excluded, and the remaining biological submissions were plotted as number of samples enriched by each phosphoproteomics method versus deposition year, colored by original-method-author status.

#### Sample blinding statement

The investigators were not blinded to group allocation during sample preparation or mass spectrometry analysis.

## Structured methods

**Table:** Reagents and tools table

Reagent/resource	Reference or source	Identifier or catalog number
**Experimental models**		
BL21 (DE3) competent *E. coli* cells	New England Biolabs	C2527H
HeLa S3 Adenocarcinoma cells	ATCC	CCL-2.2
**Recombinant DNA**		
pET28a(+) His6-Halo-TEV-sSrc SH2 transformed into NEB BL21 (DE3) *E. coli* cells	Designed in this study using the superbinder Src SH2 domain (sSrc SH2) originally described by Kaneko et al Sci. Signal. 2012 and Halo fusion protein originally described by Los et al ACS Chem Biol 2008.	
**Chemicals, enzymes and other reagents**		
Magne® HaloTag® beads	Promega	G7282
Fe-NTA MagBeads	Cube Biotech	31501-Fe
Isopropyl β-D-1-thiogalactopyranoside	Gold Bio	12481C25
benzonase nuclease │ Pierce™ Universal Nuclease	Sigma-Aldrich │ VWR	E1014-5KU │ PIER88702
B-PER Bacterial Protein Extraction Reagent	Thermo Scientific	90084
Phosphate-buffered saline tablets	Fisher Scientific	BP2944-100
Pierce™ BCA Protein Assay Kit	Thermo Fisher Scientific	23227
Sodium chloride	Sigma-Aldrich	S3014-500G
Sodium hydroxide	Fisher Scientific	S318-500
Trizma base	Sigma-Aldrich	T6066-1KG
Hydrochloric acid, 37%	Sigma-Aldrich	320331-500 ML
Water LC-MS grade	Fisher Scientific	7732-18-5
Acetonitrile LC-MS grade	Fisher Scientific	75-05-8
Methanol LC-MS grade	Fisher Scientific	A456-4
Ethanol 200 Proof (100%)	Decon Labs	2701
Formic acid LC-MS grade	Millipore Supelco	111670
Trifluoroacetic acid	Fisher Scientific	BP555-1
Acetic acid glacial	Fisher Scientific	A35-500
Ammonium hydroxide	Fisher Chemicals	A669S-500
Ferric chloride, FeCl3	Sigma-Aldrich	F2877-100G
Ethylenediaminetetraacetic acid (EDTA)	Thermo Fisher Scientific	17892
**Software**		
Comet	Eng et al, (2013)	
Percolator	Käll et al, (2007)	
Ascore	Beausoleil et al, (2006); Barente and Villén (2023)	
R	https://r-project.org/	
**Other**		
KingFisher™ Flex	Thermo Fisher Scientific	
KingFisher™ 96 KF microplate (200 µL)	Thermo Fisher Scientific	97002540
KingFisher™ 96 microtiter DW plate	Thermo Fisher Scientific	95040450
KingFisher™ 96 tip comb for DW magnets	Thermo Fisher Scientific	97002534
Vacuum concentrator	Labconco	Centrivap
Plate and microtube centrifuge	various	
Probe sonicator (optional)	Fisher Scientific	Model 100
Bath Sonicator	Branson	Model 3800
Aluminum hub blunt needles (16 G x 1.5”)	Kendall Monoject	8881202322
CDS C8 extraction Disks 3 M™ Empore™	Fisher Scientific	13-110-016

### Step-by-step protocol for phosphotyrosine peptide enrichment with R2HaPpY

Phosphotyrosine peptides were enriched using HaloTag-immobilized Src SH2 superbinder (Halo-sSrc) beads, followed by Fe(III)-IMAC cleanup, on a KingFisher Flex magnetic particle processor. A version of the protocol is maintained at 10.17504/protocols.io.q26g7n3y9lwz/v1.

***Preparation of Halo-sSrc enrichment beads***

1. Grow *Escherichia coli* BL21(DE3) carrying pET28a(+)::His6-Halo-TEV-sSrc in Luria broth with kanamycin (50 µg/mL) to OD_600_ 0.6–0.8, induce expression with 0.1 mM isopropyl β-D-1-thiogalactopyranoside (IPTG) for 20–22 h at 16–22 °C, then pellet, aliquot into 50 mL culture equivalents, and store at −80 °C. Conservatively, 50 mL of culture provides ample superbinder for 25 samples to be enriched. Note: Non-refrigerated induction (3–4 h at 37 °C) works as well, albeit with slightly reduced yield.
2. Lyse *E. coli* cells using one of two methods:
  - **Lysis method 1 (sonication):** Resuspend each 50 mL culture pellet in 4 mL ice-cold 1× PBS, pH 7.4, supplemented with 0.1 mg/mL lysozyme and 12–25 U/mL benzonase. Lyse by probe sonication (6 × 15–30 s, on ice with equal rests, 9–12 W), then clarify at ≥16,000×*g* for 15 min at 4 °C and retain the supernatant.
  - **Lysis method 2 (B-PER):** Resuspend each 50 mL culture pellet in 4 mL B-PER reagent supplemented with 0.1 mg/mL lysozyme and 12–25 U/mL benzonase, mixing by pipetting (avoid vortexing to limit foaming). Rotate for 10 min at room temperature, then clarify at 15,000×*g* for 5 min at room temperature and retain the supernatant.
3. Equilibrate Magne HaloTag beads (1 mL of 20% slurry per 4 mL lysate) with two washes in ultrapure water followed by two washes in 1× PBS, pH 7.4. Add the clarified lysate and rock end-over-end for 30 min at room temperature to covalently conjugate Halo-sSrc to the beads.
4. Discard the unbound lysate and wash the beads six times with 1–2 column volumes of 1× PBS, pH 7.4, transferring to a fresh tube after the third wash. Note: conjugated beads are stable for at least 4 months at 4 °C in 1× PBS; performance declines slightly by 10 months.
***Phosphotyrosine peptide enrichment***
5. Resuspend dried peptides (0.5–4 mg input per sample) to 1–5 mg/mL in ≤ 950 µL of 50 mM Tris, 50 mM NaCl, pH 8.0 by vortexing and bath sonication. Incubate up to 1 h for maximal resuspension, then centrifuge at ≥16,000×*g* for 10–15 min at 4 °C to pellet precipitate. Note: carry forward only the cleared supernatant, leaving ~50 µL behind to avoid transferring precipitate if present.
6. Prepare 96-well plates for robotic enrichment:
  - **Binding plate:** Equilibrate the Halo-sSrc beads with three washes in 50 mM Tris, 50 mM NaCl, pH 8.0, and dispense 40 µL of 20% bead slurry per well into the KingFisher binding plate. Load the cleared peptide solution on top of the beads.
  - **Four wash plates:** Add 850 µL 50 mM Tris, 50 mM NaCl, pH 8.0 buffer per well into three deep well plates and 850 µL HPLC-grade water into wash plate 4.
  - **Elution plate 1**: Add 100 µL of 0.5% TFA per well into a shallow well plate.
7. **Start** the R2HaPpY KingFisher Flex method. Load all plates except elution plate 2 (prepared in step 8) at the start.
8. **Prepare elution plate 2** (120 µL of 1% TFA, 60% ACN) ~120 min into the run and add it at the programmed pause (~15 min remaining). Note: delaying preparation of the organic elution limits ACN evaporation.
9. **On completion**, combine elution 2 into elution 1, leaving residual beads behind. Proceed to Fe(III)-IMAC cleanup.
***Fe(III)-IMAC cleanup of phosphotyrosine peptides***
10. Prepare 96-well plates for robotic enrichment:
  - **Binding/sample plate:** Dilute the pooled eluate to ~75% ACN by transferring the 220 µL (100 µL elution 1 + 120 µL elution 2) into 680 µL of 100% ACN in a new deep well plate, using a 96-well magnet to exclude carryover of Halo-sSrc beads.
  - **Bead plate:** Wash Fe(III)-NTA magnetic beads three times in 80% ACN, 0.1% TFA, and dispense 80 µL of 5% slurry per well into the bead plate.
  - **Three wash plates:** Add 150 µL of 80% ACN, 0.1% TFA into three shallow well plates.
  - **Tip plate:** Add a tip comb into an empty shallow well plate.
11. **Start** the Fe-IMAC cleanup KingFisher Flex protocol. Load all plates except the elution plate at the start.
  - **Prepare the elution plate** (75 µL of 2.5% ammonia, 50% ACN) 40 min into the run and add it at the programmed pause (~11 min remaining). Note: delayed addition limits the evaporation of the volatile base. Prepare the ammonia solution fresh (within 1 month) and handle it in a fume hood.
  - During the enrichment, equilibrate C8 stage tips by adding 60 µL each of MeOH, then 100% ACN, then 70% ACN/0.25% acetic acid by centrifugation for 1–2 min at 300×*g* per pass. Keep the membranes wet until use.
12. **On completion**, immediately acidify the eluates with 45 µL of 10% FA, 75% ACN, then pass them through equilibrated C8 (Empore) StageTips into mass spec vial inserts and chase with 60 µL of 70% ACN, 0.25% acetic acid into the same sample insert. Dry by vacuum centrifugation, store at ≤−20 °C, and resuspend in 12–16 µL of 4% FA, 3% ACN for MS measurement.

## Supplementary information

Appendix

Peer Review File

Dataset EV1

Dataset EV2

Dataset EV3

Dataset EV4

Dataset EV5

Source data Fig. 1

Expanded View Figures

## Data Availability

The mass spectrometry proteomics data have been deposited to the ProteomeXchange Consortium via the PRIDE partner repository with the dataset identifier PXD062515 (https://www.ebi.ac.uk/pride/archive/projects/PXD062515). To facilitate the use and dissemination of the data, we have developed a web-based resource (https://r2happy.gs.washington.edu/) in which to explore temporal phosphotyrosine profiles. The detailed enrichment protocol is accessible at 10.17504/protocols.io.q26g7n3y9lwz/v1. Datasets EV2–4 contain detailed proteomics data, including protein identifications, peptides, modification sites, and node depth assignments. R analysis code generated during the course of this study are available at https://github.com/alexis-cmyk/ManuscriptCodeShare_R2HaPpY/tree/master. The source data of this paper are collected in the following database record: biostudies:S-SCDT-10_1038-S44318-026-00843-8.
