# Supplementary material for: Scalable phosphotyrosine enrichment with SH2 superbinder enables deep profiling of EGF responses
Source: EMBO J. 2026 Jul 3;45(15):5556–79. doi: 10.1038/s44318-026-00843-8 (PMC13434655; doi:10.1038/s44318-026-00843-8)
Supplement: Supplementary file 1 [file 44318_2026_843_MOESM1_ESM.pdf]

# Appendix for
# Scalable Phosphotyrosine Enrichment with SH2 Superbinder Enables Deep Profiling of EGF Responses

# Appendix

## Table of Contents


| | |
|---|---|
| **1. Title Page** | 1 |
| 2. **Appendix Text S1**: Structural and Functional Implications of Novel pY Sites | 2-4 |
| 3. **Appendix Text S2**: Summary of Total Proteome Changes in EGF Time Course | 5 |
| 4. **Appendix Text S3**: Detailed Cost Comparison of Enrichment Reagents | 6 |
| **5. Appendix References** | 7 |
| **6. Appendix Figures** | 8-36 |
| a. Appendix Figure S1 | 8 |
| b. Appendix Figure S2 | 9 |
| c. Appendix Figure S3 | 10 |
| d. Appendix Figure S4 | 11 |
| e. Appendix Figure S5 | 12 |
| f. Appendix Figure S6 | 13 |
| g. Appendix Figure S7 | 14 |
| h. Appendix Figure S8 | 15 |
| i. Appendix Figure S9 | 16 |
| j. Appendix Figure S10 | 17 |
| k. Appendix Figure S11 | 18 |
| l. Appendix Figure S12 | 19 |
| m. Appendix Figure S13 | 20 |
| n. Appendix Figure S14 | 21 |
| o. Appendix Figure S15 | 22 |
| p. Appendix Figure S16: Circular Plasmid Map | 23 |
| q. Appendix Figure S17: Linear Plasmid Map | 24-36 |
| r. Appendix Figure S18: Annotated Spectra of Novel pY Sites | 37-49 |

# Appendix Text S1:
# Structural and Functional Implications of Novel pY Sites

## Overview

A sensitive and scalable phosphotyrosine (pY) enrichment method enables both discovery and quantitative comparisons of tyrosine phosphorylation dynamics across time, as presented here, or across other variables such as drug treatments and patient tissue. In this extended text, we discuss the potential structural and functional relevance of a few novel pY sites identified in our temporal profiling of EGF response, based on literature, protein structure, and sequence conservation. This analysis lays out the foundation for future mechanistic studies.

First, we highlight phosphosites in endocytosis. Upon activation, EGFR is rapidly sorted through early endosomes and ultimately degraded in lysosomes to attenuate its signal (Raiborg & Stenmark, 2009). Aberrant downregulation of EGFR allows unchecked signaling, which has been shown to precipitate disease (Schultz *et al*, 2023; Tang *et al*, 2013; Fichera *et al*, 2007).

We quantified phosphorylation events in many proteins involved in endocytic sorting of EGFR, including the discovery of 26 novel pY sites (Appendix Figure S12).

We discuss two novel sites: RNF126 pY27 and HGS pY197. We describe how RNF126 pY27 may play a role in recognition of ubiquitin linkage type on EGFR during its passage from early endocytosis to late endocytosis based on structural alignment and prior functional studies. We additionally identified novel pY on pY197 of HGS/HRS which likely directly interacts with PI3P phospholipid head group in early endosomes, given its position in the FYVE domain.

Last, we highlight a dual phosphorylation in the kinase WEE1 that may be involved in regulating WEE1 activity. Collectively, these examples illustrate how this dataset can be used for novel hypothesis generation of potentially functional phosphosites.

## Novel phosphorylation of Tyr27 in E3 Ligase RNF126 may interact with Lys48 of ubiquitin

Successful processing and degradation of EGFR requires the E3 ubiquitin ligase RNF126. As EGFR transits from the early endosome to the late endosome, RNF126 binds monoubiquitinated EGFR via its N-terminal zinc finger domain (residues 1-40) and regulates ubiquitin chain extension of K63-linkage type to successfully traffic EGFR to the lysosome for degradation (Smith *et al*, 2013). Importantly, deletion of RNF126 causes retention of EGFR in the late endocytic compartment indicating the ubiquitin recognition and ligase activity are critical for proper degradation of activated EGFR (Smith *et al*, 2013).

We identified a novel phosphorylation site on tyrosine 27 in RNF126's ubiquitin binding zinc finger (Appendix Figure S13A). This was exciting because Y27 of RNF126 is already known to participate in two hydrogens bonds with the ubiquitin-like domain of BAG6 as part of the BAG6 sortase complex that sends mis-localized membrane proteins for degradation (Krysztofinska *et al*, 2016). This motivated structural interrogation of the potential binding interface of RNF126 with ubiquitin. Given that no structure of RNF126 bound to ubiquitin has been reported to date, we superimposed ubiquitin (PDB ID: 2BGF, chain A) onto BAG6 ubiquitin-like domain (PDB ID 2N9P, chain C). Alignment was quite good for ubiquitin and BAG6 ubiquitin-like domain (RMSD = 1.9Å). Strikingly, Y27 of RNF126 appeared equidistant from Lys48 in ubiquitin compared to previously described hydrogen bond interactions of Gln62 and Arg64 of BAG6 ubi-like domain (Appendix Figure S13B).

Given the role of RNF126 in regulating ubiquitin topology for timely trafficking of EGFR through late endocytosis, we hypothesize phosphorylation of tyrosine 27 may help position ubiquitin for K63 linkage by binding strongly to K48. To our knowledge, no one has reported the interaction between ubiquitin K48 and tyrosine or phosphotyrosine of a ubiquitin binding zinc finger. To investigate the potential of this interaction as a broader mechanism, we point out that RNF126 zinc finger shares homology with Rabring7 (also known as BCA2, RNF115, or ZNF364) for which conserved Y36 positions similarly relative to a predicted ubiquitin interaction (Appendix Figure S13C). Interestingly,

Rabring7 is also capable of binding to ubiquitinated EGFR, although is not required for proper endocytic sorting (Smith *et al*, 2013). Accordingly, we see one instance of phosphorylation at Y36 in Rabring7. While this site is also novel, it was measured at relatively low abundance and did not pass our filtering criteria of minimum three observations in any condition. Although subtle, the structural conservation and additional detection leave open the possibility of a shared ubiquitin recognition mechanism among zinc fingers of both RNF126 and Rabring7 during endosomal sorting of EGFR.

**Novel phosphorylation of Y197 in HRS/HGS is positioned to replace PI3P interaction**
In the early endosome, EGFR is concentrated by the ESCRT-0 complex for further sorting. ESCRT-0 consists of HRS (also known as HGS) and STAM proteins, which form heterotetramers and bind ubiquitinated cargo (Mayers *et al*, 2011). The FYVE domain of HGS binds phosphatidylinositol 3-phosphate (PI3P) head groups, which are enriched on the early endosome lipid membrane. Within FYVE domains, specific conserved amino acids bind PI3P headgroups (Hayakawa *et al*, 2004). Despite conserved residues, the ability of different FYVE domains to bind endosomes remains variable. Variable interactions are thought to be determined by non-conserved residues distinguishing spatio-temporal control of endosomal membrane interactions (Hayakawa *et al*, 2004). Here we observe a novel phosphorylation of Y197 in the FYVE domain of HGS that reaches maximal measured intensity late into stimulation, at 15 minutes (Appendix Figure S14A-B). HGS Y197 is flanked by the FYVE defining residues yet is one of the variable residues differentiating FYVE domains of different proteins such as Fab1, YotB, Vac1p, and EEA1 (Appendix Figure S14C). Within HGS, Y197 is conserved through zebrafish indicating HGS-specific importance (Appendix Figure S14D). Strikingly, when mapped onto a crystal structure of human HRS/HGS FYVE domain (PDB 4AVX), this phosphorylation is predicted to occupy the same space as phosphate on the PI3P head group (Appendix Figure S14E, right). We hypothesize phosphorylation of Y197 may release HGS from endosomal membranes despite rich PI3P content due to electrostatic repulsion or local pH increase (Appendix Figure S14E, left). Indeed, pH change modulates affinity of HGS and the closely related EEA1 FYVE domain for endosomal membranes (He *et al*, 2009; Lee *et al*, 2005). Importantly, regulation of HRS/HGS FYVE endosomal binding is not fully elucidated; specifically, discrepancy between in vitro and in vivo membrane binding is not fully understood (Hayakawa *et al*, 2004; He *et al*, 2009). Taken together, observation of this novel pY site highlights mechanistic clues that can be learned about in vivo phosphorylation dynamics in fundamental processes such as endocytic receptor regulation using this scalable and streamlined pY enrichment method.

**Novel dual phosphorylation of WEE1 kinase at S396 and Y397**
WEE1 kinase serves as a cell cycle checkpoint and is highly expressed in numerous cancers. When expressed, WEE1 phosphorylates CDK1 on Y15 to prevent mitotic entry. A current approach for targeting cancer inhibits WEE1 activity to induce mitotic catastrophe by forcing cells to progress despite damaged DNA. Targeting has traditionally focused on the ATP binding pocket. We identified novel dual phosphorylation at S396 and Y397 of WEE1, in a specific yet understudied loop between D and E helices, offering a potential handle for its specific regulation (Appendix Figure S15A-B). While the exact function of the dual phosphorylation is unclear, we highlight the sites reside between helices relevant for both ATP binding and peptide substrate recognition (Zhu *et al*, 2017; Arter *et al*, 2022) (Appendix Figure S15B). Ultimately, the loop between D and E helices in WEE1 kinase and its relatives WEE2, CHK1, MYT1 and PLK1 lacks PTM information (Appendix Figure S15C) and identification of these novel phosphorylation sites should motivate inquiry of this understudied loop.

## Appendix Text S2
## Summary of total proteome changes in EGF stimulation timecourse

To account for potential phosphosite abundance changes driven by changes in protein abundance, differential abundance testing was performed on the total proteome data derived from the EGF stimulation time course of HeLa cells. As expected for short treatment durations, the proteome minimally changed. Only 41 proteins showed significant abundance differences of which only one protein, GOLIM4, contained a regulated phosphotyrosine site. The GOLIM4 protein increases in abundance by 1.3 and 1.4-fold at 5 and 15 min of EGF treatment, respectively. However the phosphorylation of Y483 on GOLIM4 decreases by 8.3 to 8.8-fold at 5 and 15 min of EGF treatment. The protein abundance increase is minimal and only exacerbates the abundance decrease of phosphorylation on tyrosine 483. Given only one phosphotyrosine site was affected by a protein abundance change and adjustment moderately amplified the phosphosite abundance decrease as well as if adjusted would not have drastically changed the observed trend, we mention this in the text but did not account for it in the figures.

## Appendix Text S3
## Detailed cost comparison of enrichment reagents

Cell Signaling Technology® (CST®) sells 400 µL of magnetic beads conjugated pTyr- 1000 multi-mAb for $431 and prescribes use at a ratio of 1:20 which equates to 50 µL of bead slurry per 1 mL peptide sample. This translates to $53.8 per sample or $5172 for 96 samples and does not require any in-house labor costs. Cost calculation for EasyAb comparison accounted for recommended use of 100 µL CST PTMScan® magnetic bead slurry per sample resulting in cost of $30,480 for 96 samples (Jayavelu et al 2024). This calculation is based on $2,540 per 800 µL bead slurry (Catalog #8803). Preparation of NHS-sSrc beads according to our 2023 publication requires purchase of Pierce™ NHS activated magnetic beads at $226 per 1 mL of bead slurry. Each milliliter of bead slurry supplies 12.5 samples given 80 µL of beads are required per sample.

This equates to $18 per sample or $1728 for 96 samples in magnetic bead costs. Purification of superbinder requires TALON® resin and minimum of two Amicon centrifugal concentrators. We quoted TALON® resin at $796 for 50 mL and expect use of 5 mL resin to purify enough superbinder for 96-samples which adds $80 in resin costs. We estimated each concentrator to cost $15. For E. coli labor, we estimated preparation of 6L *E. coli* culture to require 8 hours of labor with labor estimated at $30 per hour. We found 1L of *E. coli* culture to yield 25 mg of purified His-sSrc protein and 96 samples requires 16 mg of purified His-sSrc protein. Therefore, the labor cost of growing *E. coli* was adjusted for 0.62 L given cell pellets can be divided and stored frozen. We added an additional $20 for bead quality control (QC) to account for SDS PAGE analysis and 660 nm assay reagent. In total, preparation of NHS-sSrc beads for 96 samples requires an estimated $2604 of which 34% ($876) is accrued from in-house costs.

In contrast, Halo-sSrc beads cost $553 under the same assumptions. Given Promega Magne® HaloTag® beads cost $121 per mL of bead slurry (at the time of writing) and each sample requires 40 µL of bead slurry, the cost per sample of unconjugated magnetic beads is $4.84 or $465 for 96 samples. We use 50 mL of *E. coli* culture to conjugate 1 mL of magnetic bead slurry leaving the labor cost of *E. coli* culture at $7.7 per 96-well plate. This holds the same assumption as above that a 6L *E. coli* culture requires 8 hours of labor at $30 per hour in which cell pellets are divided and stored frozen. Preparation of Halo-sSrc beads requires two hours of labor costing $60 for 96 samples assuming $30/hr. Halo-sSrc bead preparation does not require any purification reagents. We included $20 for bead quality control by TEV digest with SDS PAGE analysis. In total, preparation of Halo-sSrc beads cost $553 for 96 samples of which 16% ($88) are related to in-house bead preparation.

**Appendix References: Structural and functional implications of novel pY sites**


Arter C, Trask L, Ward S, Yeoh S & Bayliss R (2022) Structural features of the protein kinase domain and targeted binding by small-molecule inhibitors. *J Biol Chem* 298: 102247

Fichera A, Little N, Jagadeeswaran S, Dougherty U, Sehdev A, Mustafi R, Cerda S, Yuan W, Khare S, Tretiakova M, *et al* (2007) Epidermal growth factor receptor signaling is required for microadenoma formation in the mouse azoxymethane model of colonic carcinogenesis. *Cancer Res* 67: 827–835

Hayakawa A, Hayes SJ, Lawe DC, Sudharshan E, Tuft R, Fogarty K, Lambright D & Corvera S (2004) Structural basis for endosomal targeting by FYVE domains. *J Biol Chem* 279: 5958–5966

He J, Vora M, Haney RM, Filonov GS, Musselman CA, Burd CG, Kutateladze AG, Verkhusha VV, Stahelin RV & Kutateladze TG (2009) Membrane insertion of the FYVE domain is modulated by pH. *Proteins* 76: 852–860

Krysztofinska EM, Martínez-Lumbreras S, Thapaliya A, Evans NJ, High S & Isaacson RL (2016) Structural and functional insights into the E3 ligase, RNF126. *Sci Rep* 6: 26433

Lee SA, Eyeson R, Cheever ML, Geng J, Verkhusha VV, Burd C, Overduin M & Kutateladze TG (2005) Targeting of the FYVE domain to endosomal membranes is regulated by a histidine switch. *Proc Natl Acad Sci U S A* 102: 13052–13057

Mayers JR, Fyfe I, Schuh AL, Chapman ER, Edwardson JM & Audhya A (2011) ESCRT-0 assembles as a heterotetrameric complex on membranes and binds multiple ubiquitinylated cargoes simultaneously. *J Biol Chem* 286: 9636–9645

Raiborg C & Stenmark H (2009) The ESCRT machinery in endosomal sorting of ubiquitylated membrane proteins. *Nature* 458: 445–452

Schultz DF, Billadeau DD & Jois SD (2023) EGFR trafficking: effect of dimerization, dynamics, and mutation. *Front Oncol* 13: 1258371

Smith CJ, Berry DM & McGlade CJ (2013) The E3 ubiquitin ligases RNF126 and Rabring7 regulate endosomal sorting of the epidermal growth factor receptor. *J Cell Sci* 126: 1366–1380

Tang J, Liu N & Zhuang S (2013) Role of epidermal growth factor receptor in acute and chronic kidney injury. *Kidney Int* 83: 804–810

Zhu J-Y, Cuellar RA, Berndt N, Lee HE, Olesen SH, Martin MP, Jensen JT, Georg GI & Schönbrunn E (2017) Structural basis of Wee kinases functionality and inactivation by diverse small molecule inhibitors. *J Med Chem* 60: 7863–7875

## Appendix Figures

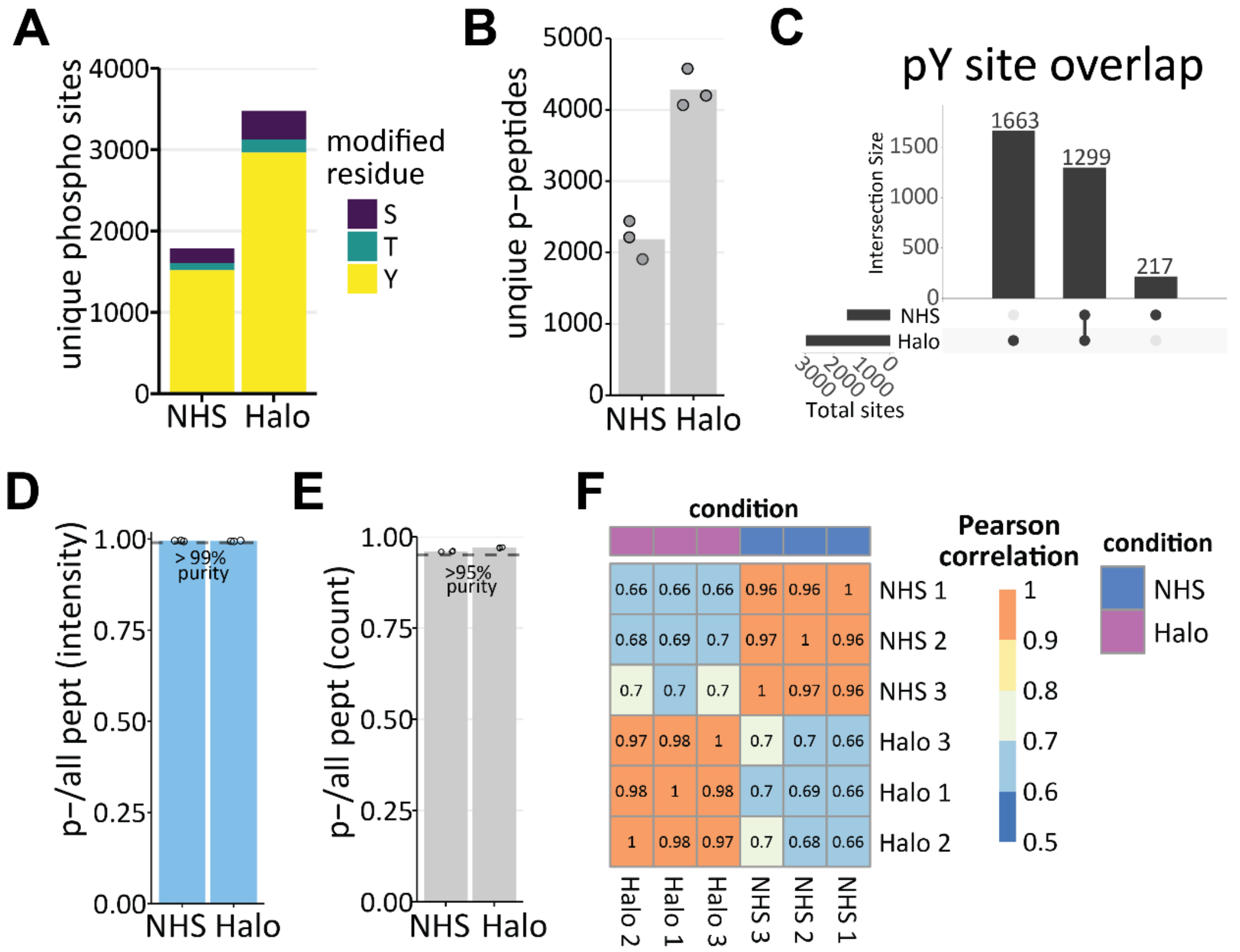


**Appendix Figure S1. Additional metrics for the comparison of Halo-TEV-sSrc beads to NHS-sSrc beads.** NHS-sSrc and Halo-sSrc beads were directly compared by enriching pY peptides from 1 mg of pervanadate-treated HeLa digest per replicate, for a total of three replicates per bead type.

- **A.** Net unique phosphosites colored by modified residue across three technical replicates per bead type. Phosphoserines are colored purple, phosphothreonines are teal, and phosphotyrosines are yellow.
- **B.** Counts of unique phosphopeptides with at least one confidently localized phosphosite. Points indicate replicates and height of bar indicates average number of phosphopeptides considering all three replicates.
- **C.** Intersections of unique phosphotyrosine sites between bead types across all three replicates.
- **D.** Enrichment efficiency based on intensity of phosphorylated peptides relative to all peptides measured.
- **E.** Enrichment efficiency based on the count of phosphorylated peptides relative to all peptides measured.
- **F.** Pearson correlation (R) of pY site intensities between bead types and technical replicates.

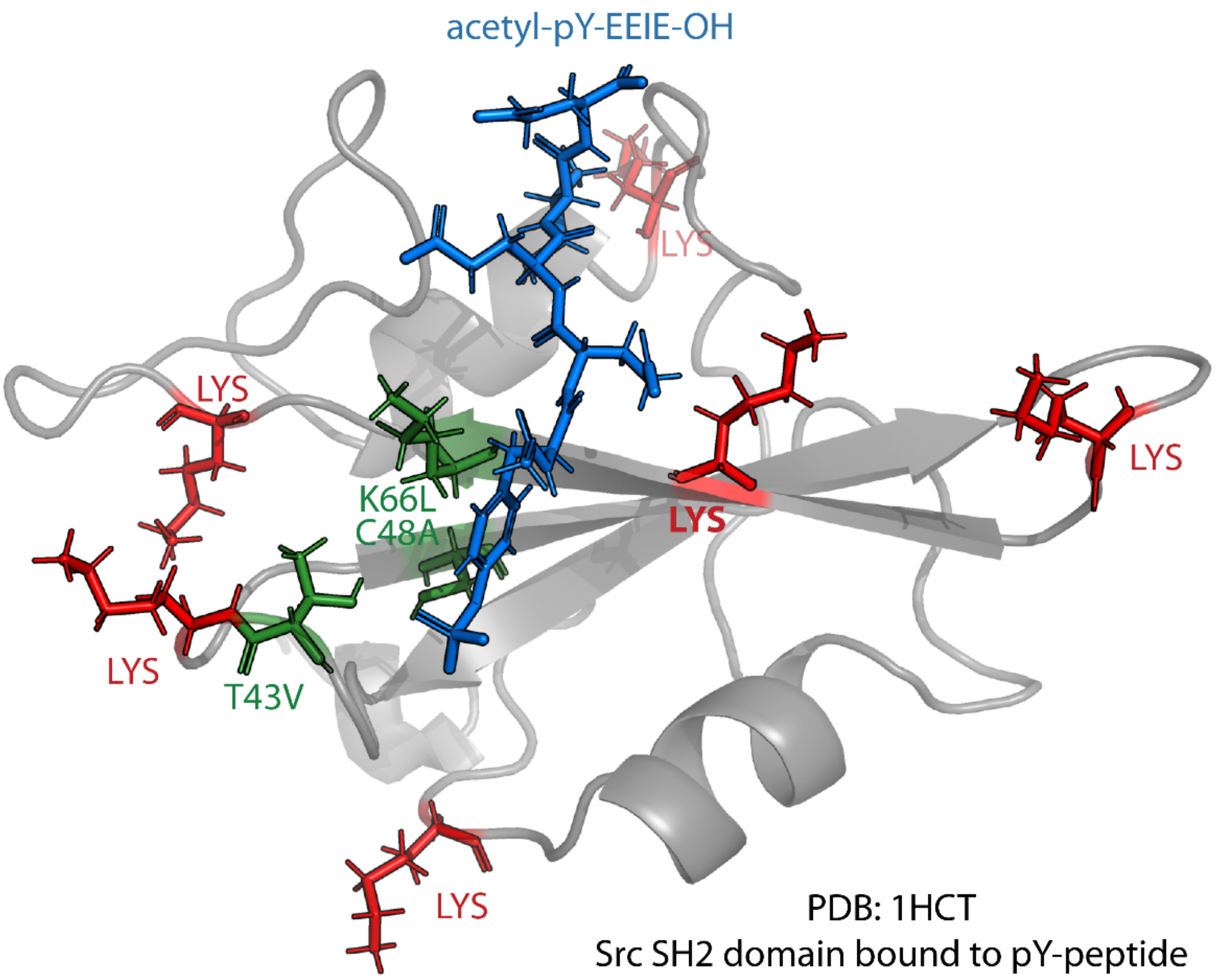


**Appendix Figure S2. Location of lysines in the Src SH2 pY superbinder relative to a short pY peptide.** Wild type sSrc SH2 with acetyl-pY-EEIE-OH peptide in blue (PDB: 1HCT). Residues mutated in superbinder are shown in green; lysines shown in red. One of the six lysines in Src SH2 superbinder directly contacts the bound pY peptide (bolded lysine). NHS conjugation likely occludes the pY peptide binding pocket in 16% of sSrc molecules based on probability of conjugation between sSrc and magnetic beads at this site.

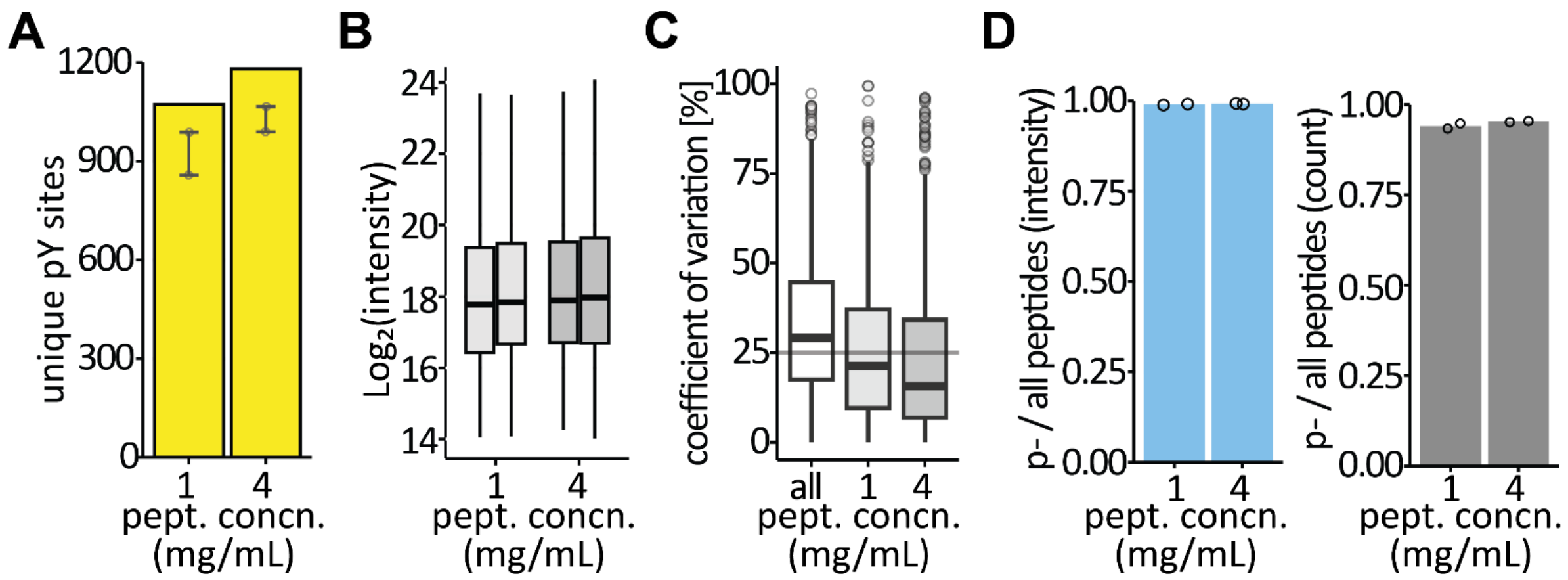


**Appendix Figure S3. R2pY Halo is robust to low and high peptide concentration even for non-pervanadate treated HeLa lysate.**

**A.** Bar plot indicates net unique pY sites enriched from two replicates of untreated HeLa cells. Each replicate was enriched from 4 mg of peptide input, except diluted to different concentrations, either 1 mg/mL or 4 mg/mL. Points indicate number of unique pY sites enriched per replicate. Whiskers indicate range of pY sites enriched per replicate with center of error bar indicating average pY site count per replicate.

**B.** Boxplots indicate intensity distributions of pY peptides per replicate for each peptide input concentration.

**C.** Boxplots indicate distributions of percent coefficient of variation for pY peptides between replicates for each peptide concentration. The white boxplot on left indicates the variation of individual pY peptides across both conditions.

**D.** Measurement of sample purity. Ratios of phosphopeptides relative to all peptides detected by either intensity (left) or count (right) indicates relative enrichment of phosphopeptides. Bar height indicates average enrichment efficiency across triplicates, while points indicate enrichment efficiencies of individual replicates.

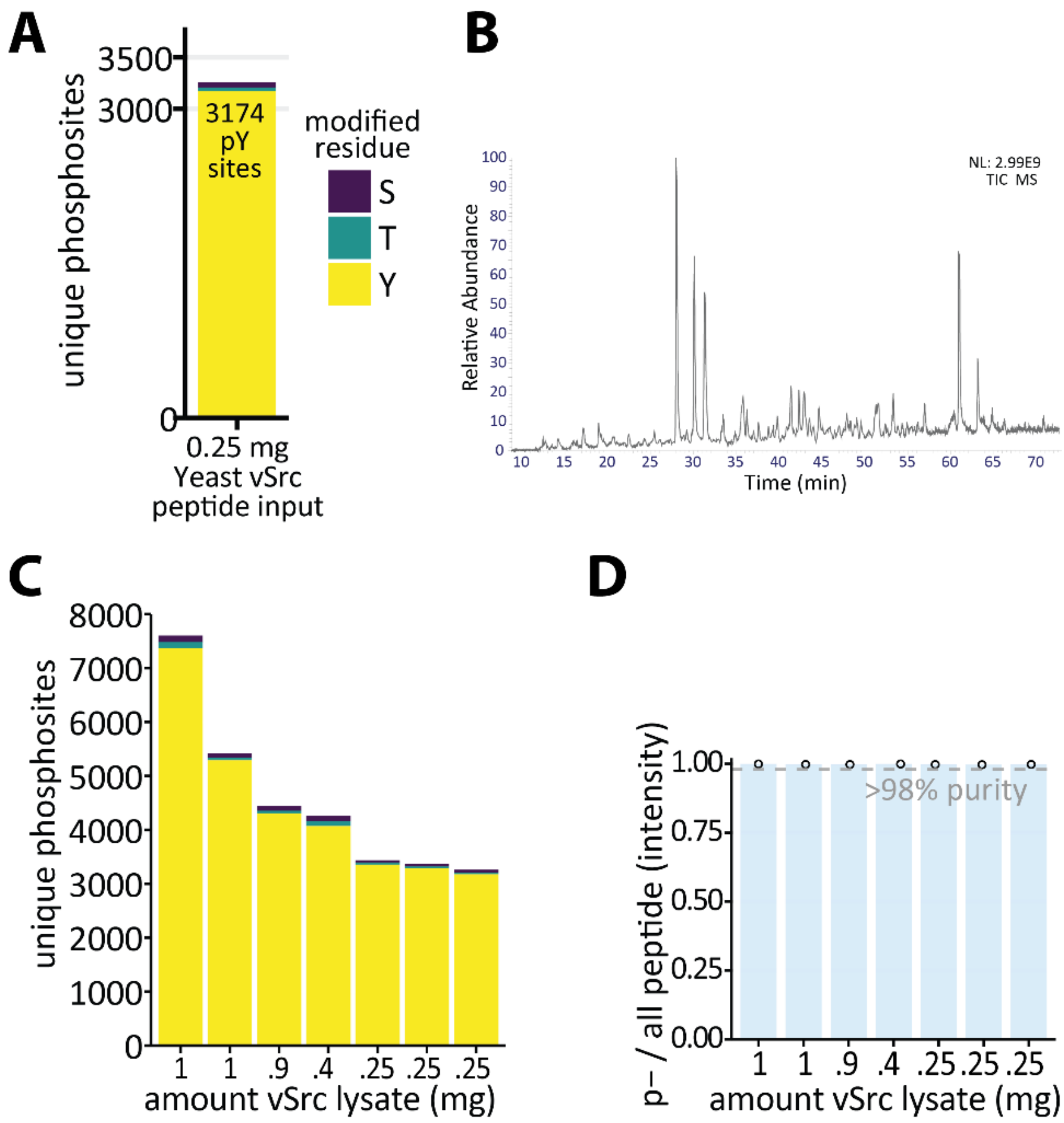


**Appendix Figure S4. Benchmarking yeast vSrc positive control sample**

**A.** Barplot indicates number of unique phosphosites enriched from 0.25 mg of yeast vSrc lysate with 40 µL of Halo-sSrc beads. Modified residues were indicated by color. Number of unique phosphotyrosine sites listed on the plot.

**B.** Example chromatogram for the yeast vSrc positive control sample. Zoomed into the linear gradient. Trace is of total ion current from full MS scans. Maximal intensity is listed on the chromatogram.

**C.** Results from 7 different batches of Halo-sSrc beads tested by enriching pY from yeast vSrc lysate. Different peptide input amounts and injection volumes were used to test different batches, however we find 0.25 mg of input lysate and injecting 60% of the same is the best to assess bead quality. Each bar representes a different batch of Halo-sSrc beads. Amount of yeast vSrc lysate used in each test of beads is listed below each bar.

**D.** Barplot indicates the ratio of phosphopeptides intensity relative to intensity of all peptides quantified in each sample. All bead batches produced high purity samples indicated by >98% phosphopeptide enrichment efficiency (gray dotted line). Points indicate individual measurements; only one replicate per batch is shown.

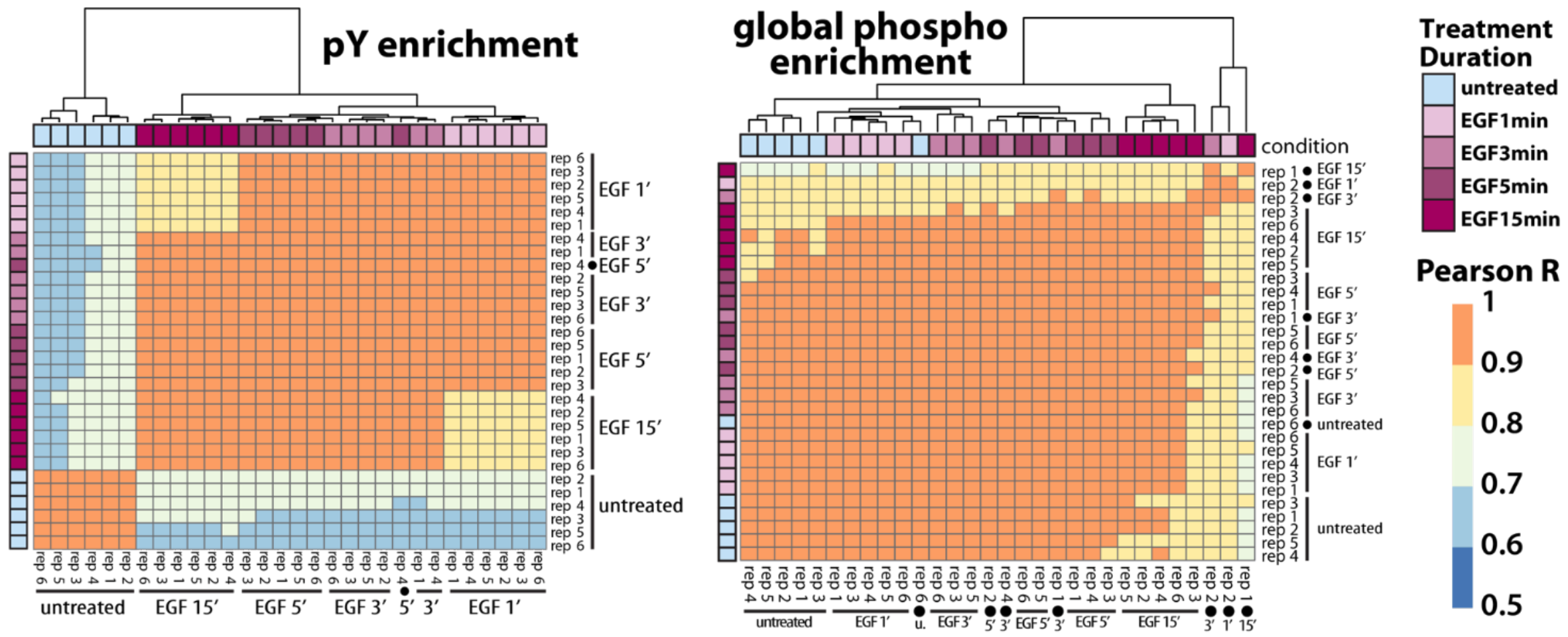


**Appendix Figure S5. pY specific enrichment better distinguished EGF treatment durations compare to global phosphopeptide enrichment.** Heatmaps of Pearson correlations of confidently localized phosphosites for phosphotyrosine specific enrichment (left) and global phosphopeptide enrichment (right) from EGF treatment time course. EGF treatment duration is indicated by light blue to dark pink colors at top and left of plot. Correlation coefficients between any two samples are indicated by color gradient. In the left plot, only phosphotyrosine sites were included, while for global enrichment any phosphoserine, phosphothreonine, and phosphotyrosine were included. Dots on y- and x-axis indicate replicates that clustered between replicates of a different condition. In contrast, lines indicate two or more replicates from the same EGF treatment duration that clustered together.

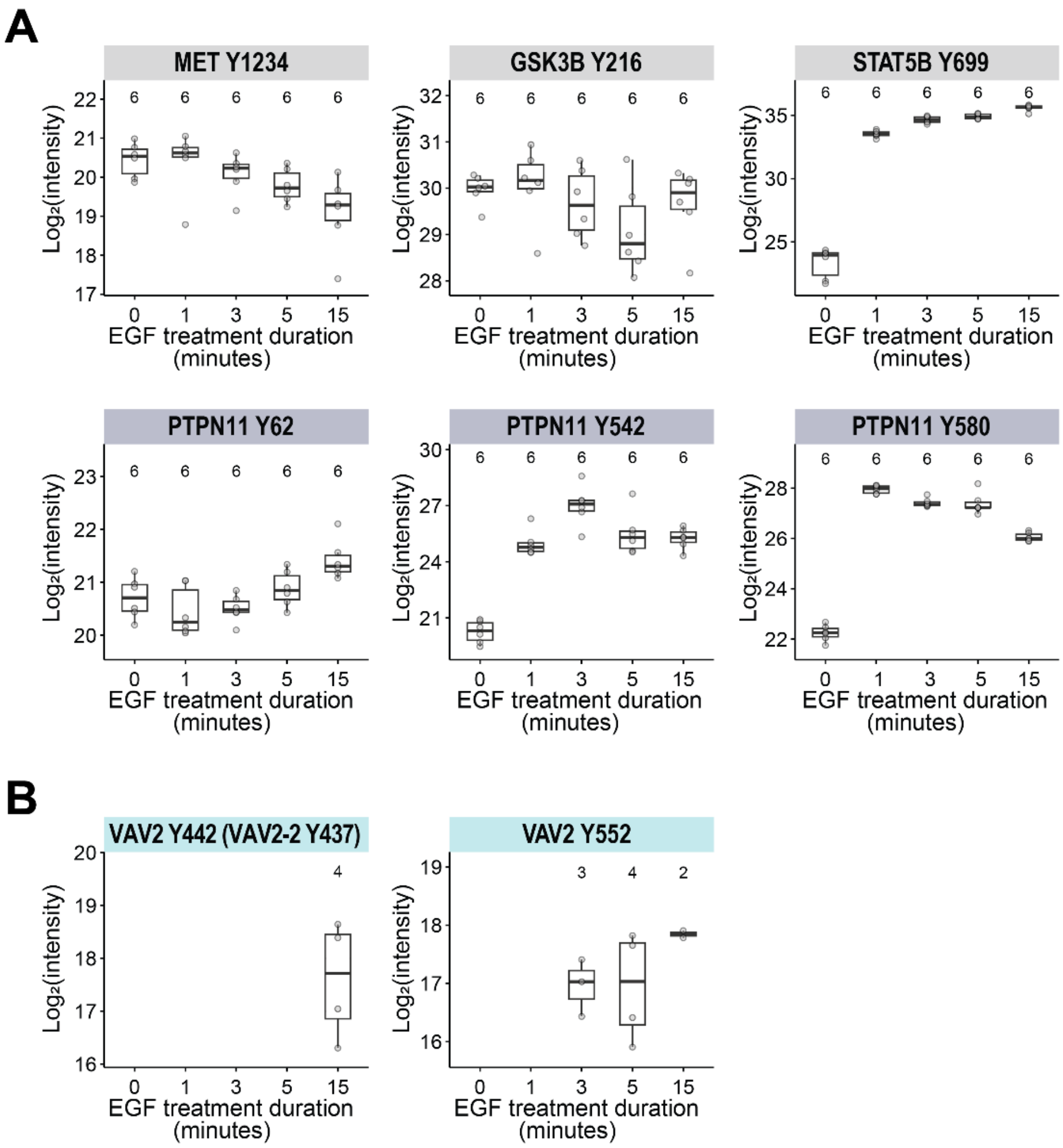


**Appendix Figure S6. Known and novel pY site comparison to EasyAb**

- **A.** pY sites on known EGF responsive proteins detected in this study and by Jayavelu *et al* (2024). Background color of plot titles are gray for proteins with a single pY site detected and purple for multiple pY sites per protein.
- **B.** Novel pY sites detected in this study and Jayavelu *et al* (2024) included VAV2-2 Y437. We also detected VAV2 Y552 on the same protein, but not detected by Jayavelu *et al* (2024).

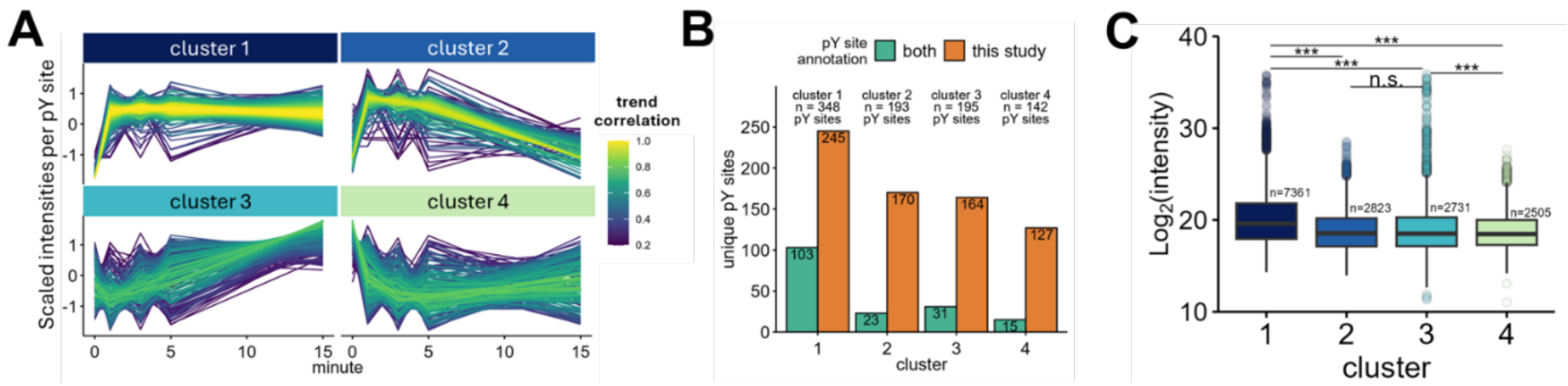


**Appendix Figure S7. Extended characterization of pY-site temporal responses**.

**A.** Line plots indicate profiles of individual phosphotyrosine sites within each cluster. The y-axis depicts scaled intensity and x-axis EGF treatment duration. Timepoint 0 indicates untreated HeLa cells. Profiles are colored according to their correlation with the average cluster trend; dark blue indicates low correlation and yellow indicates high correlation.

**B.** Counts of pY per cluster, separated by prior annotation status in either of two EGF response databases (PhosphoSite Plus and PTMSigDB). Annotation status of 'both' (green) indicated the pY site was both found regulated by EGF in this study and was previously annotated as EGF responsive in at least one of the two EGF response databases (PhosphoSite Plus and PTMSigDB). Sites observed regulated only in this study and not in either database were labelled 'this study' and colored orange.

**C.** Boxplots show intensity distributions of pY sites in each cluster. Numbers above the upper box bound indicate total number of observations noting individual replicates were counted separately. Lines at top of the plot indicate comparisons tested for significant difference by Welch's two-sample t-test with Bonferroni correction. *** indicates adjusted $p$-value < 0.0001; n.s. indicates intensity distributions were not significantly different.

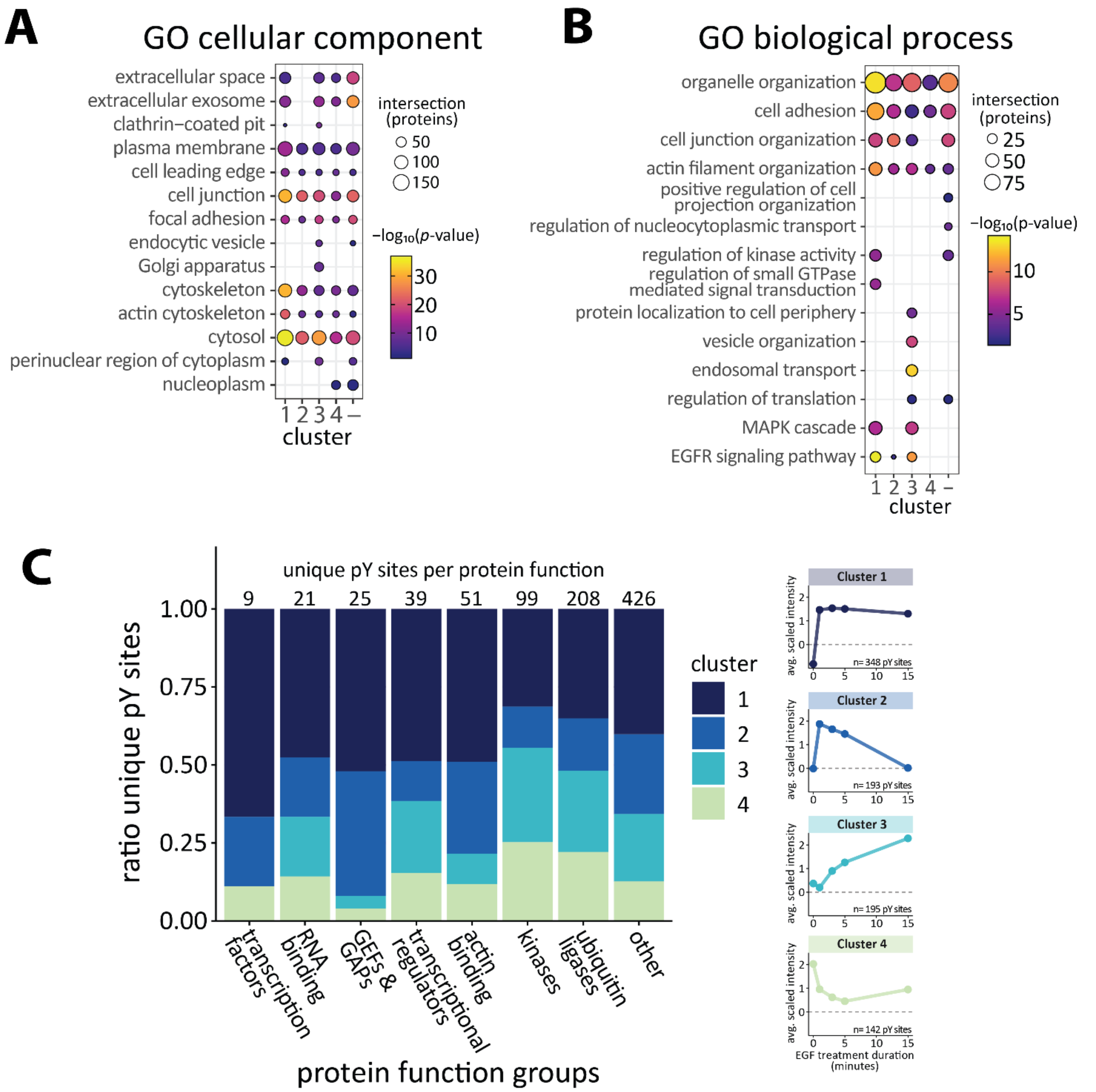


**Appendix Figure S8. Distribution of protein functions by pY site intensity profile**

**A.** Enrichment analysis of gene ontology cellular component terms for proteins with tyrosine phosphorylation. Enrichment analyses were performed separately for pY sites regulated by EGF and belonging to each cluster as well as for sites not significantly regulated by EGF (dash on x-axis) and were visualized together. Terms were manually curated to avoid redundancies and remove overly broad terms. Enrichment was performed with gprofiler2.

**B.** Enrichment analysis of gene ontology biological process terms for proteins containing pY sites observed in this study. Analysis was performed same as for panel A.

**C.** Bar plots indicate relative contributions of pY site temporal profiles per protein function category. Categories correspond to those chosen by Olsen *et al* (2006). Total numbers of EGF-regulated pY sites belonging to each protein function category are listed at top of bars. Cluster temporal profiles copied from Figure 5B are shown for reference.

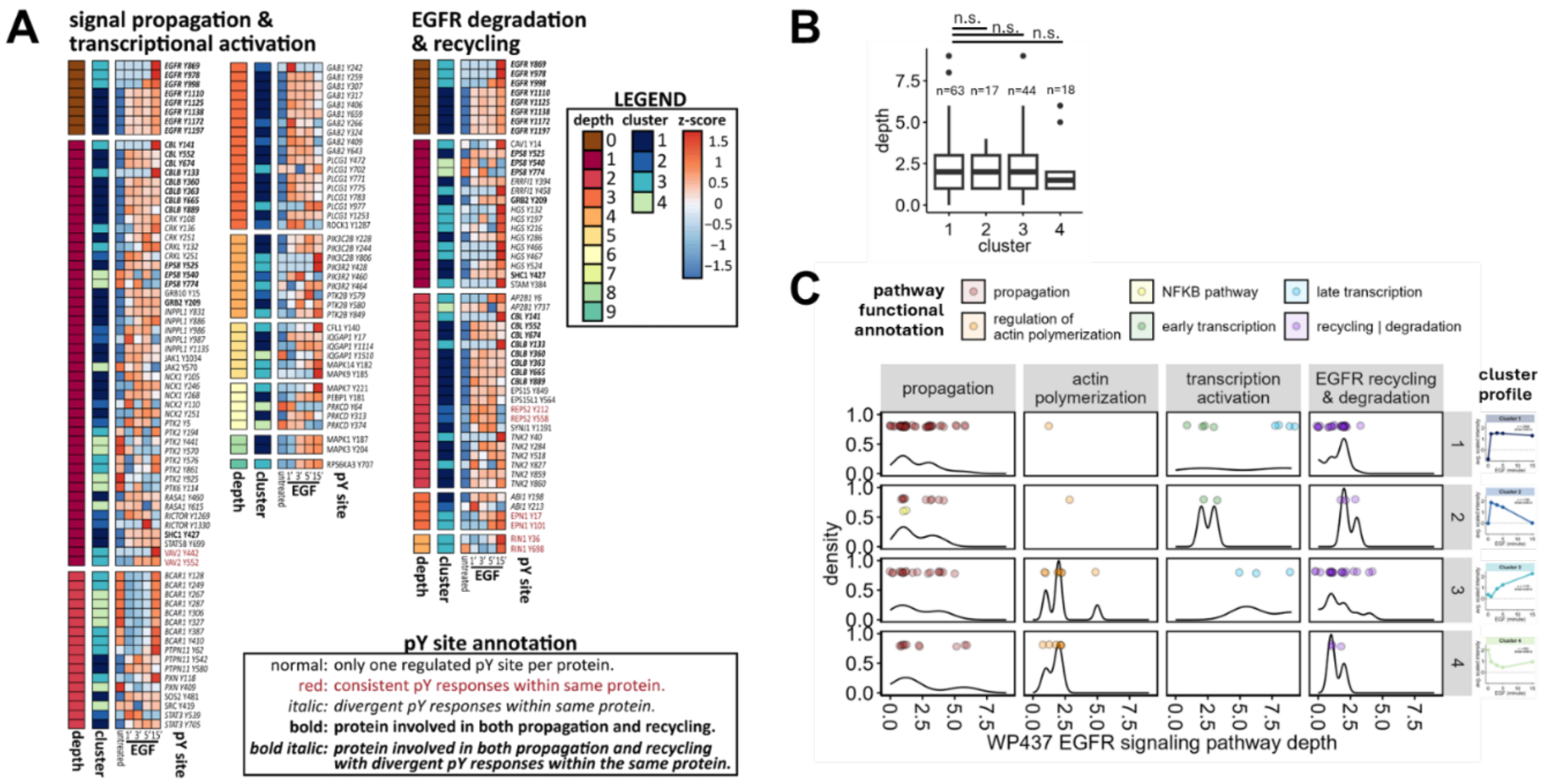


**Appendix Figure S9. Temporal response profiles of pY sites do not correlate with node depth per WP437**

**A.** Signaling pathway depths were manually assigned to proteins mapped in the WikiPathway 437 (WP437) by counting nodes along edges extending from EGFR. Proteins involved in signal propagation *en route* to transcriptional activation were visually separated from proteins involved in the internalization, degradation and recycling of EGFR on the left and right of the figure and are denoted by headers.
We coupled the temporal response profiling displayed in Figure 5 with the node depth analysis by plotting a heatmap with the assigned depth, assigned temporal response cluster (see Figure 5), and the scaled average intensities of pY sites at each EGF treatment duration (untreated, 1 min, 3 min, 5 min, or 15 min EGF application). The text format of the protein and phosphotyrosine site annotations were differently encoded to indicate characteristics about phosphotyrosine site response profiles. Specifically, normal text indicates a single pY site was found to be regulated on the protein. Red text indicates a minimum of two pY sites were found to regulated on the same protein with the same response profile. Bold text indicates the protein is involved in both the signal propagation and recycling arms. Bold italic text indicates the protein is involved in both signal propagation and receptor recycling arms of the pathway and that multiple pY sites were found to be regulated with different temporal response profiles.

**B.** Boxplot indicates distribution of depths (y-axis) assigned to individual pY sites, separated by cluster membership (x-axis). Distributions of pY site depths were not significantly different between clusters (Welch's t-test, *$p < 0.05$).*

**C.** Proteins were grouped into broad functional categories based on annotations within WP437 and mapped along node depth (x-axis) relative density of sites (y-axis). pY sites were separated by temporal response profile cluster in the vertical direction and by broad protein function in the horizontal direction. Points above the density plots denoted individual pY sites positioned along the x-axis according to their node depth and colored according to their broad protein category.

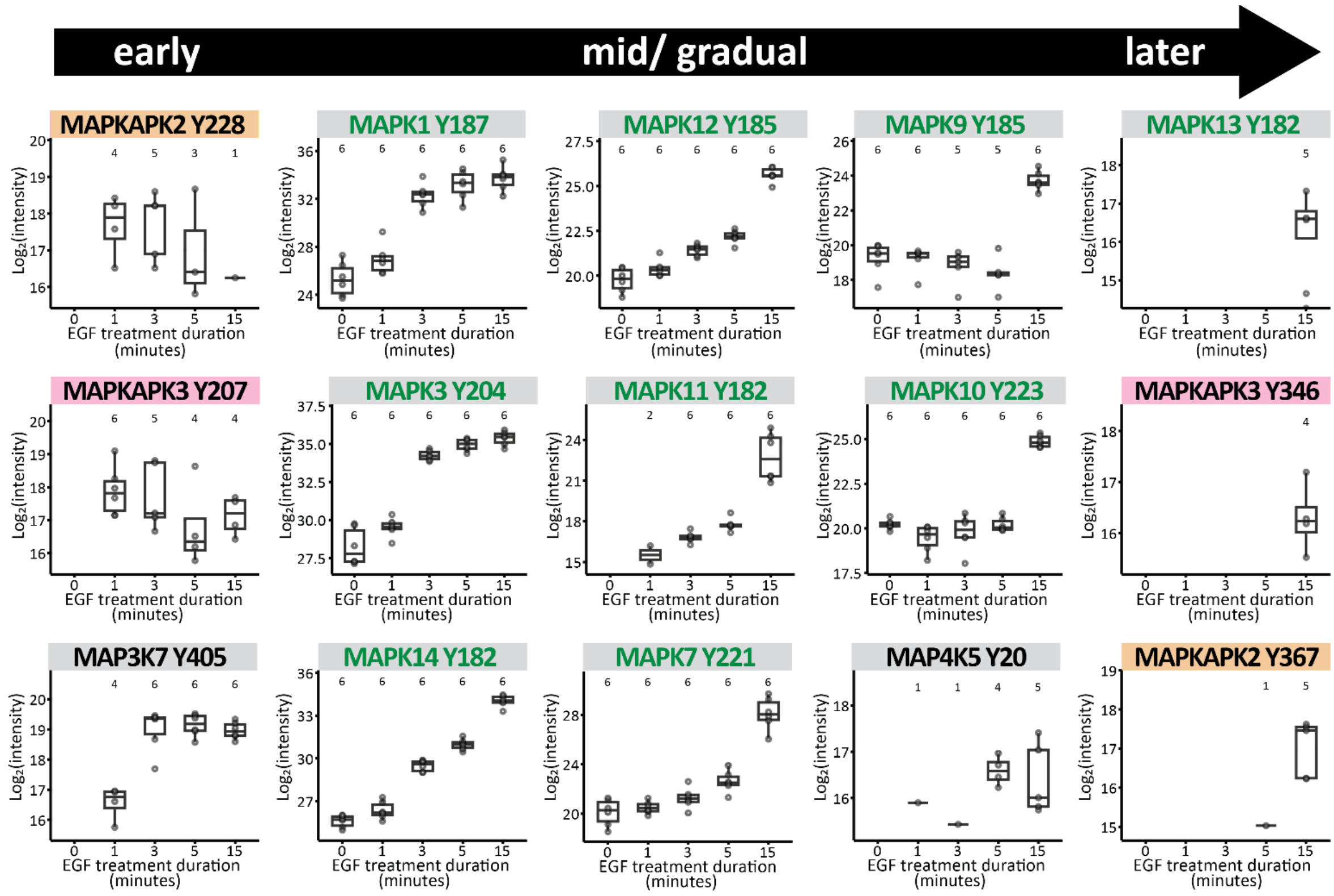


**Appendix Figure S10. MAPK and MAPKAPK proteins show a range of pY temporal profiles.**
Boxplots indicate distributions of pY site intensities (y-axis) per EGF treatment duration (x-axis). The pY sites are organized left to right according to duration required for maximal pY site abundance change. Gene name and pY site location within the full protein sequence is listed on the title of each plot. Green title text indicates the pY site was known to activate kinase activity, while the black title text indicates unknown function. The background color of the title is gray if only one pY site was detected on the protein while colors correspond to proteins with multiple pY sites detected. Number of replicates in which a pY site was observed is listed near the top of each plot. Absence of a number indicates the pY site was not observed at the time point.

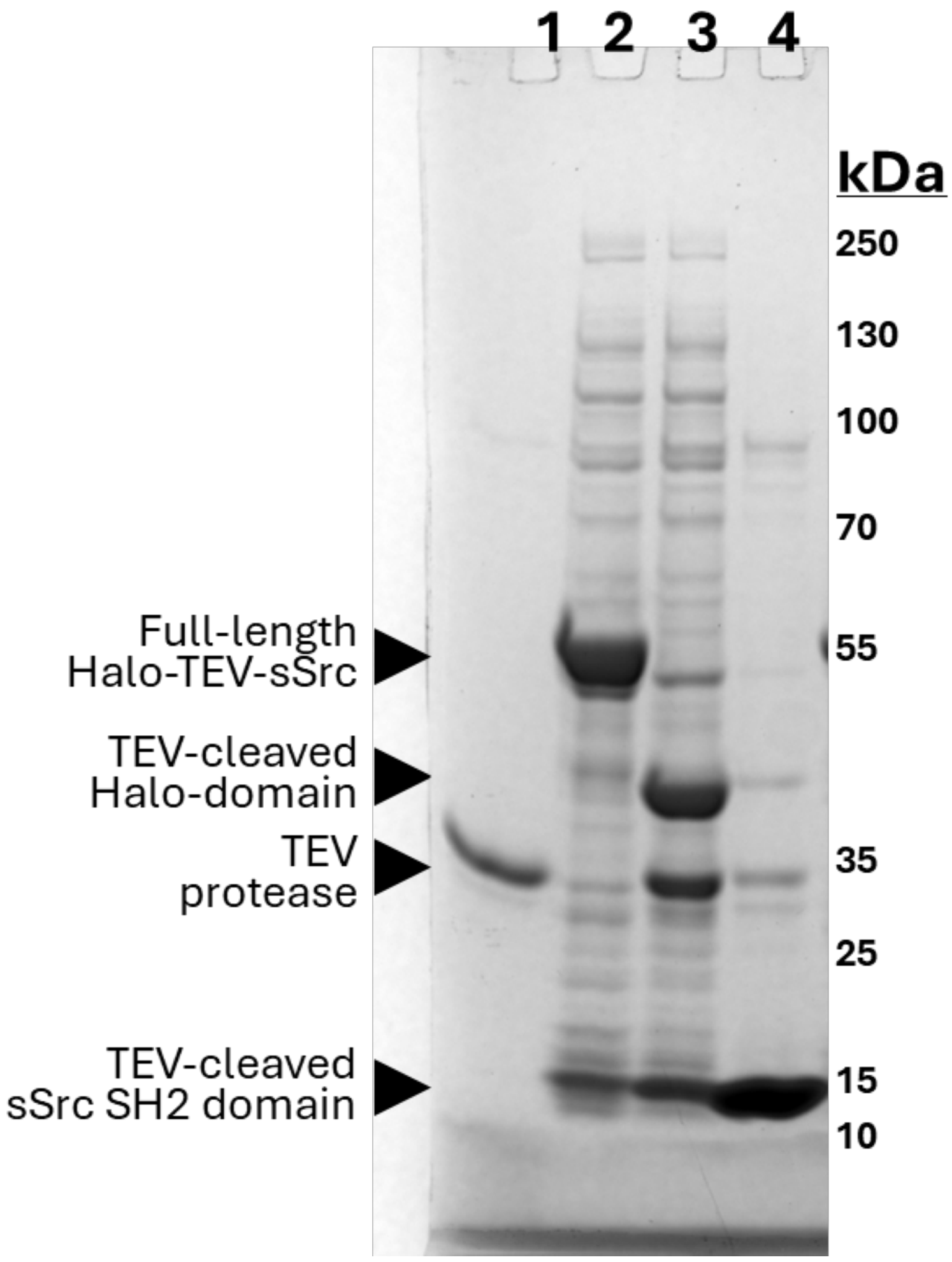


**Appendix Figure S11. TEV protease digest of Halo-TEV-sSrc conjugated to magnetic beads separates sSrc SH2 domain from Halo tag domain.**
Lane 1: 40 ng of TEV protease alone 27 kDa (apparent 30 kDa).
Lane 2: Control for full length Halo-TEV-sSrc in E. *coli* lysate.
Lane 3: TEV cleavage of Halo-TEV-sSrc in E. *coli* lysate. Both 33 kDa HaloTag (apparent 37 kDa) and 14 kDa sSrc SH2 domain visualized alongside 27 kDa (apparent 30 kDa) TEV protease.
Lane 4: TEV cleavage of bead-bound Halo-TEV-sSrc. sSrc SH2 domain appears at 14 kDa while the HaloTag remains bound to magnetic beads.
Sizes of PAGERuler Plus 10 – 260 kDa molecular weight marker are indicated on the right of the gel image.

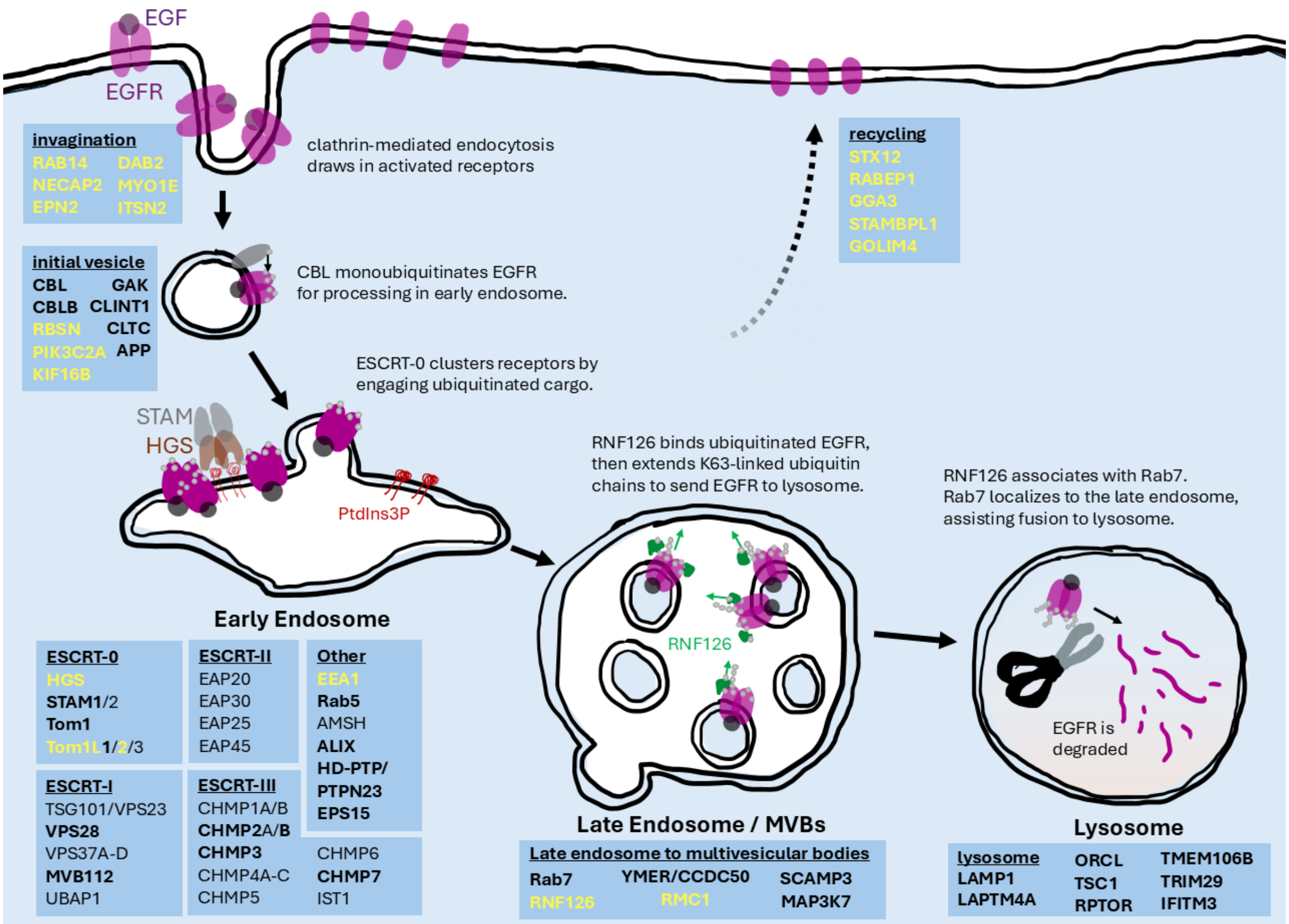


**Appendix Figure S12. Overview of EGFR endocytosis machinery highlighting phosphotyrosine site coverage.** Simplified schematic of endocytosis based on several sources. For each step, relevant proteins are listed in boxes. Proteins for which we measured known pY sites are bold and proteins with at least one novel pY site are colored yellow. Non-bolded protein names indicate proteins for which we did not detect a phosphotyrosine site in this study yet are known players of the process. Not all known proteins are listed. Illustrations depict proteins we highlighted in the text, specifically HGS/HRS (brown) and RNF126 (green).

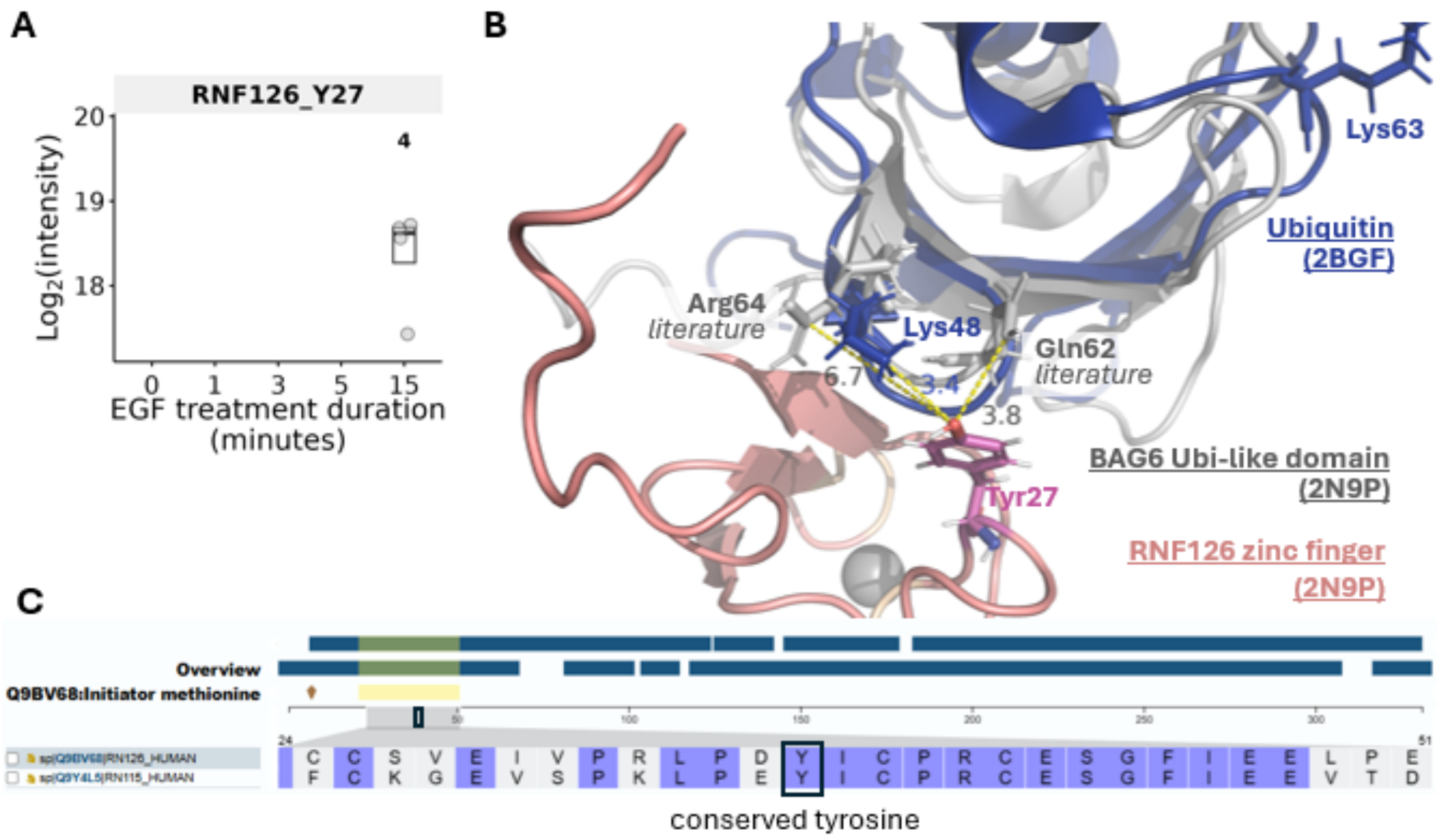


**Appendix Figure S13. Novel phosphotyrosine 27 of RNF126 positions near Lys48 of ubiquitin.**

**A.** Abundance measurements of phosphotyrosine 27 of RNF126 through EGF time course indicate intensity increase at 15 minutes EGF treatment. Confident detection made in 4 of 6 replicates indicated by '4' inside plot.

**B.** Superposition of ubiquitin (PDB ID: 2BGF, chain A; blue) onto BAG6 ubiquitin-like domain (PDB ID: 2N9P, chain C; gray); RMSD = 1.89Å. RNF126 (PDB ID: 2N9P, chain A, pink) shows Tyr27 highlighted in bright pink with distance measurements between relevant residues in Angstroms.

**C.** Multiple sequence alignment of RNF126 and RNF115 (Rabring7) showing conservation of Y27 and Y36 in zinc finger domains, respectively.

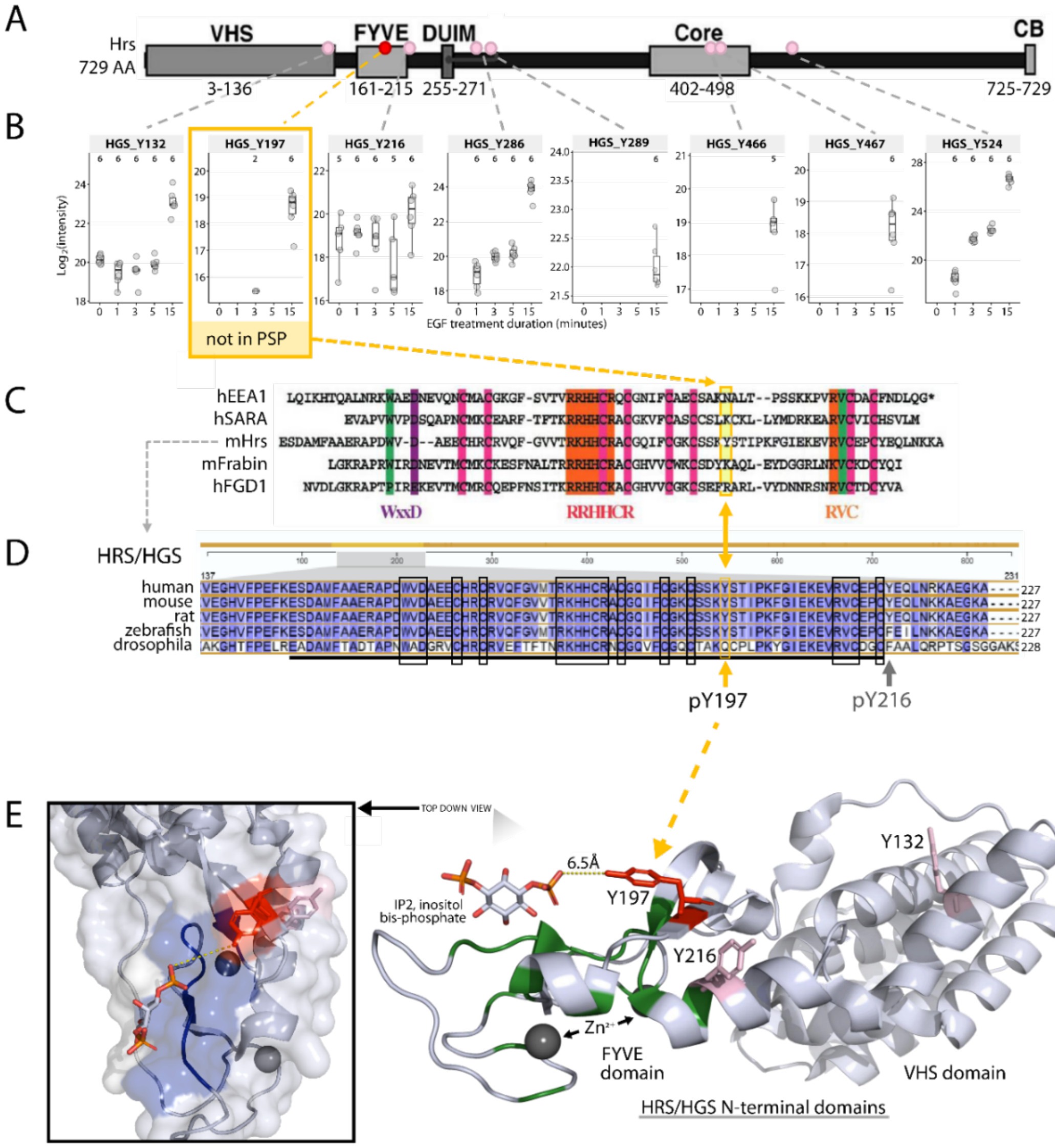


**Appendix Figure S14. Novel pY197 in HRS/HGS FYVE phospholipid binding domain is positioned for abrogation of PI3P interaction.**

**A.** Domain structure of HRS/HGS from Mayers *et al* 2018 annotated with observed phosphotyrosine sites as points; red is the novel pY 197 while the previously measured pY sites are shown in pink.

**B.** Boxplots and points indicate measured intensities of each pY site along HRS/HGS. The novel pY197 is boxed in yellow; this site is not listed in PhosphoSitePlus.org (PSP).

**C.** Sequence alignment of FYVE domains from five different proteins, mouse (m) or human (h) analyzed by Hayakawa *et al* 2009. Highlighted residues are conserved FYVE domain defining residues. Novel phosphorylation of Y197 of HRS/HGS is highlighted yellow.

**D.** Multiple sequence alignment of FYVE domains of HRS/HGS from human, mouse, rat, zebrafish, and drosophila melanogaster, top to bottom.

**E.** Structure of human HRS/HGS FYVE and VHS domains bound to inositol bis-phosphate (IP2) (PDB 4AVX). Endogenously, HGS dimerizes and binds phosphatidyl inositol 3 phospholipid head groups of early endosomal membranes instead of IP2. Right: Conserved FYVE domain defining residues are colored green. Tyrosines with measured pY are shown as sticks with novel pY197 colored red and previously observed pY sites colored pink. Only pY sites within FYVE or VHS domains are shown on structure. Left: Top down view of PIP3 binding pocket. Histidines and arginines known to contact PI3P are colored blue while Y197 is colored red. Proximity of Y197 to positively charged binding pocket illustrates potential for electrostatic or pH adjustment by phosphorylation.

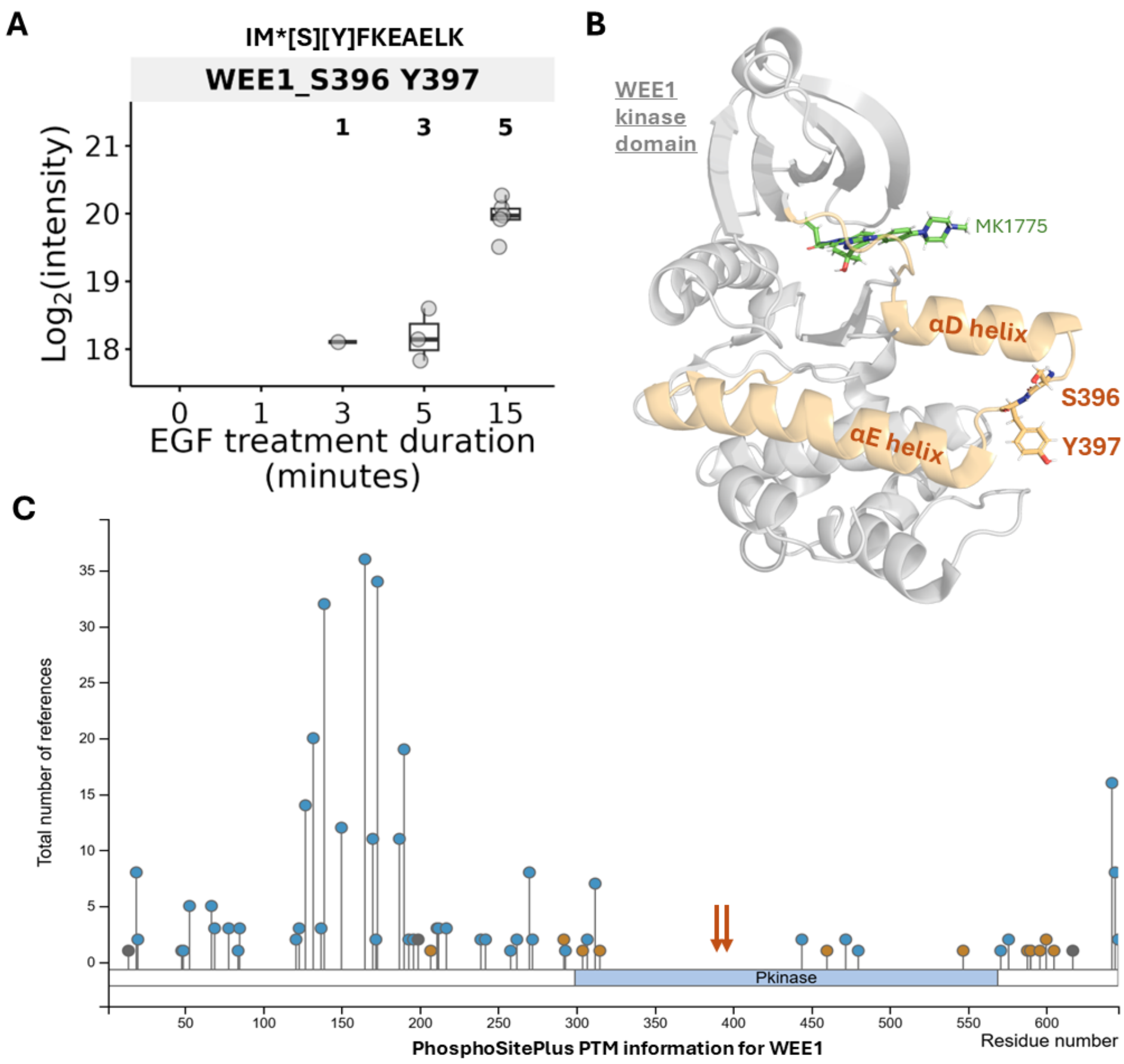


**Appendix Figure S15. Novel dual phosphorylation in WEE1 kinase observed in a vastly understudied loop between helices D and E.**

**A.** Measured intensities of the doubly phosphorylated peptide. The observed phosphopeptide is displayed at top of plot; asterisk is methionine oxidation, and brackets are phosphorylation sites. Points represent measurement from individual replicates while boxplot summarizes distribution of measured intensities for each EGF treatment duration. Inset numbers at top of plot indicate number of measured biological replicates out of six possible.

**B.** Structure of WEE1 kinase domain with sites of novel phosphorylation, S396 and Y397 shown as sticks. WEE1 is bound to the ATP-competitive inhibitor, MK1775 (green). Alpha helices D and E are connected by loop containing S396 and Y397 (colored orange).

**C.** Lollipop plot downloaded from PhosphositePlus.org on Oct 20th, 2025 for WEE1 protein. Orange arrows indicate location of novel pS396 and pY396.

## Halo-sSrc *E. coli* Expression Construct

**Appendix Figure S16: Circular Plasmid Map**

**Halo_TEV_sSrc in pET-28a(+) (6522 bp)**

5/16/2023 4:53:57 PM

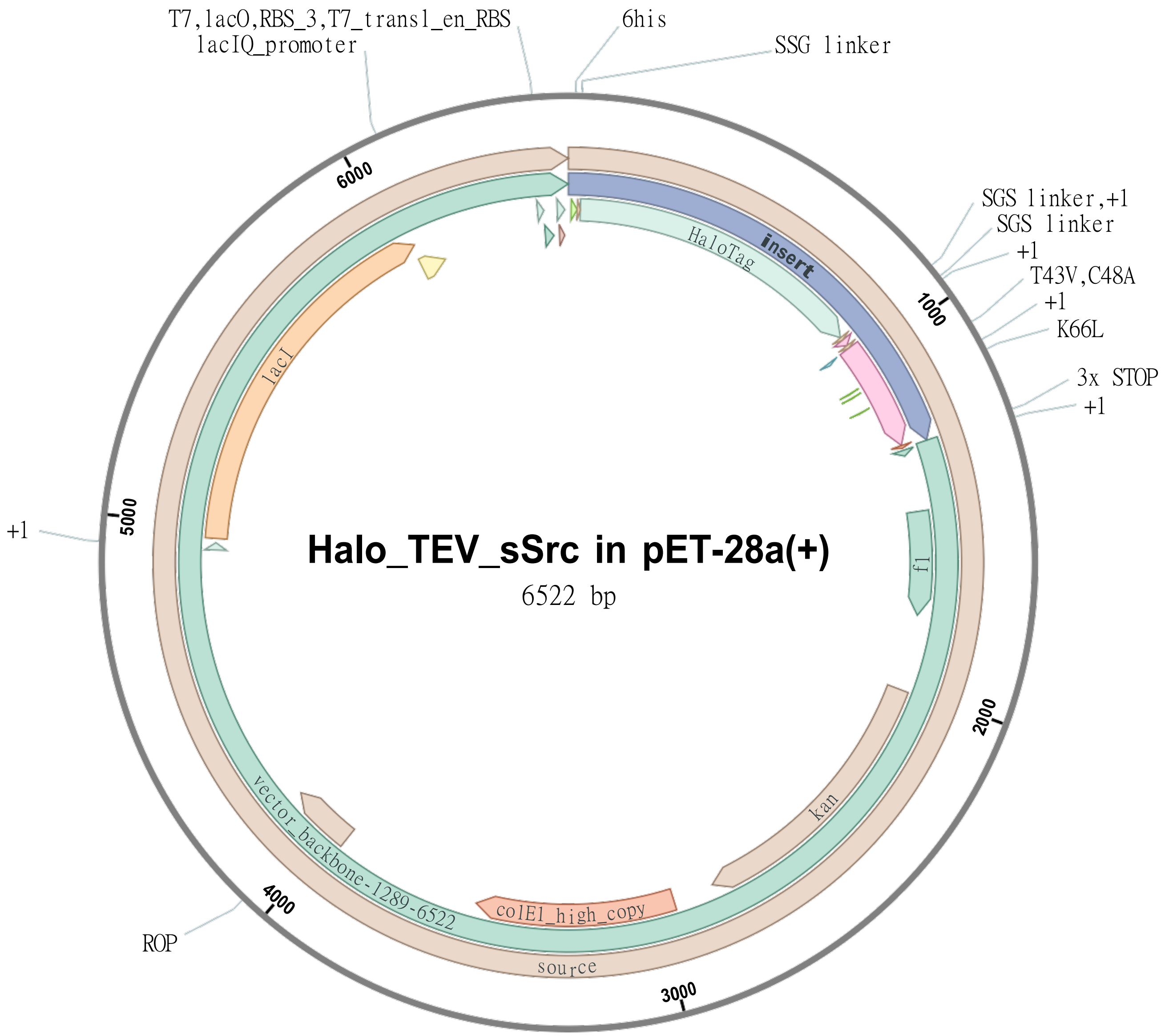

## Halo-sSrc *E. coli* expression construct

## Appendix Figure S17: Linear Plasmid Map (from 1-321 bp)

1/23/2025 7:22:27 PM

Halo_TEV_sSrc in pET-28a(+) (6522 bp)

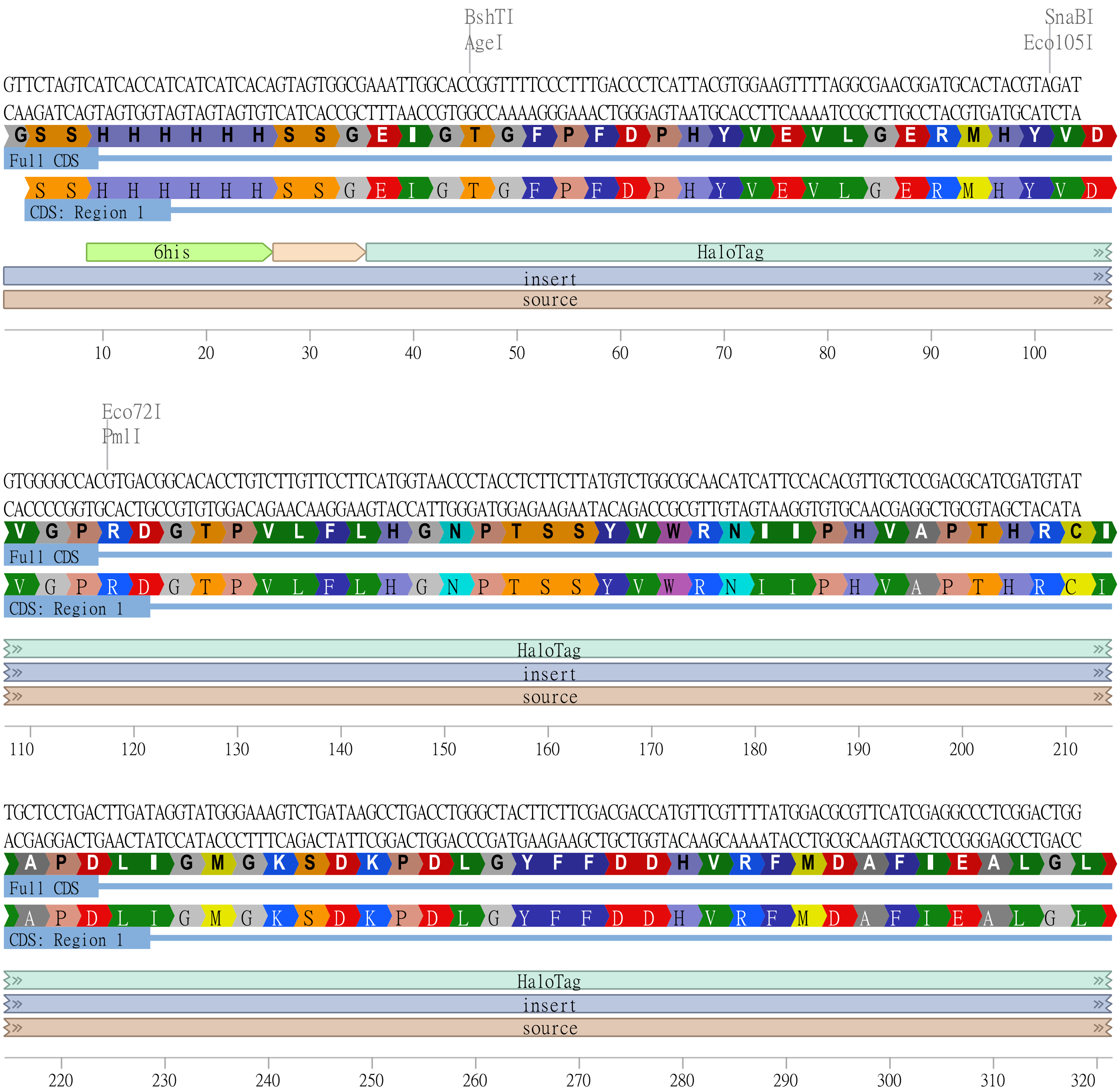

## Halo-sSrc *E. coli* expression construct

Halo_TEV_sSrc in pET-28a(+) (6522 bp) (from 322-749 bp) 1/23/2025 7:22:27 PM

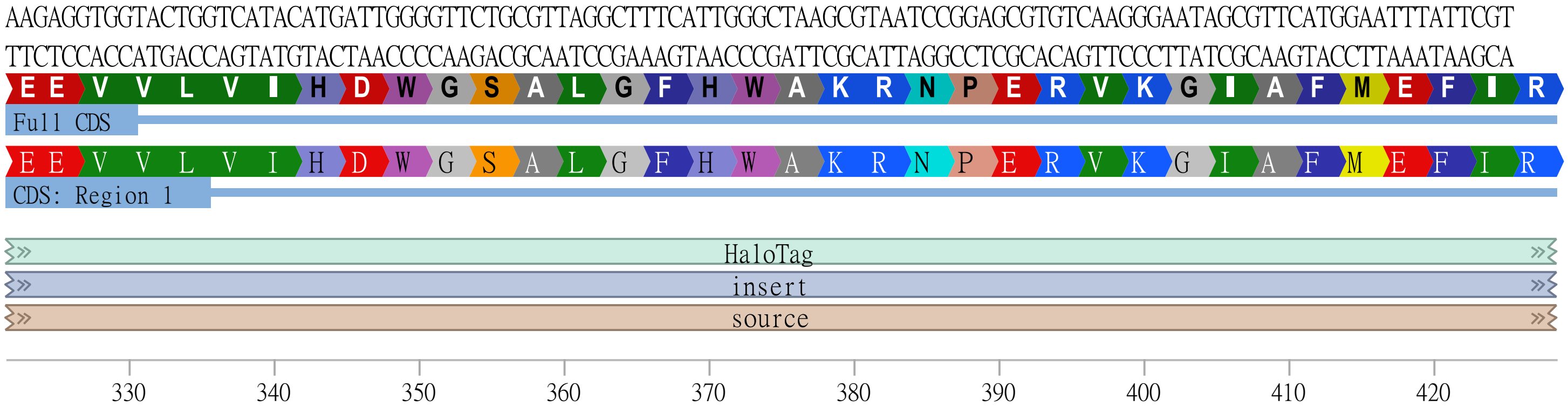


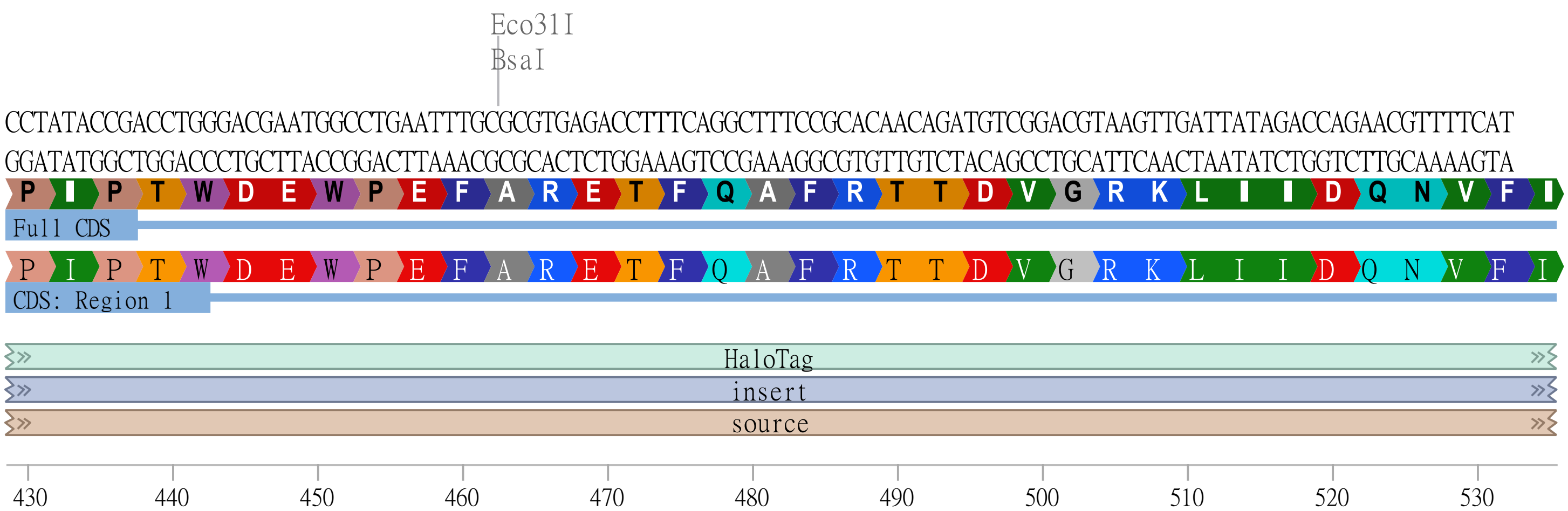


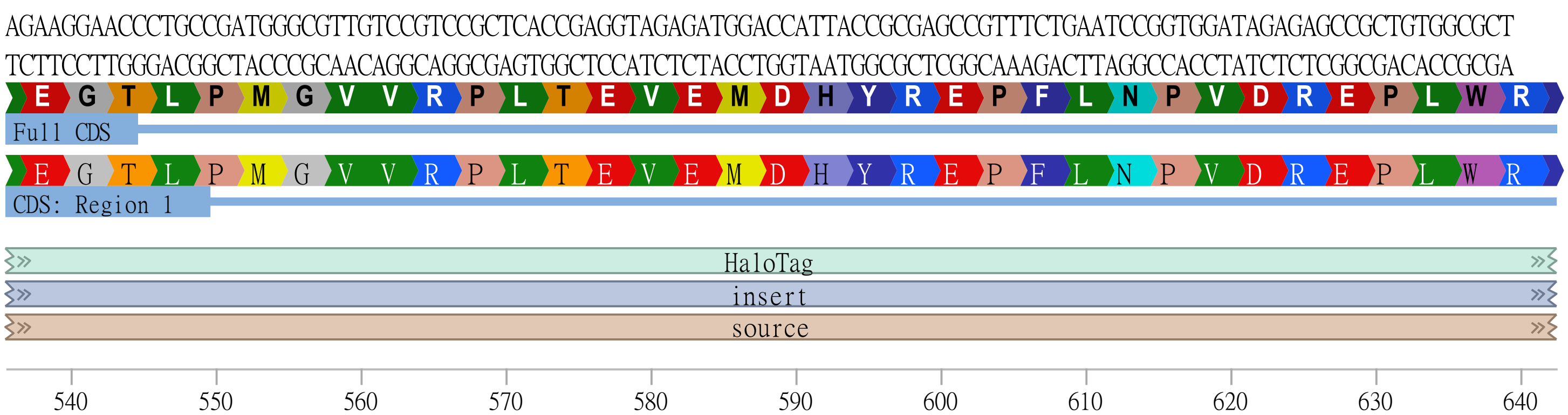


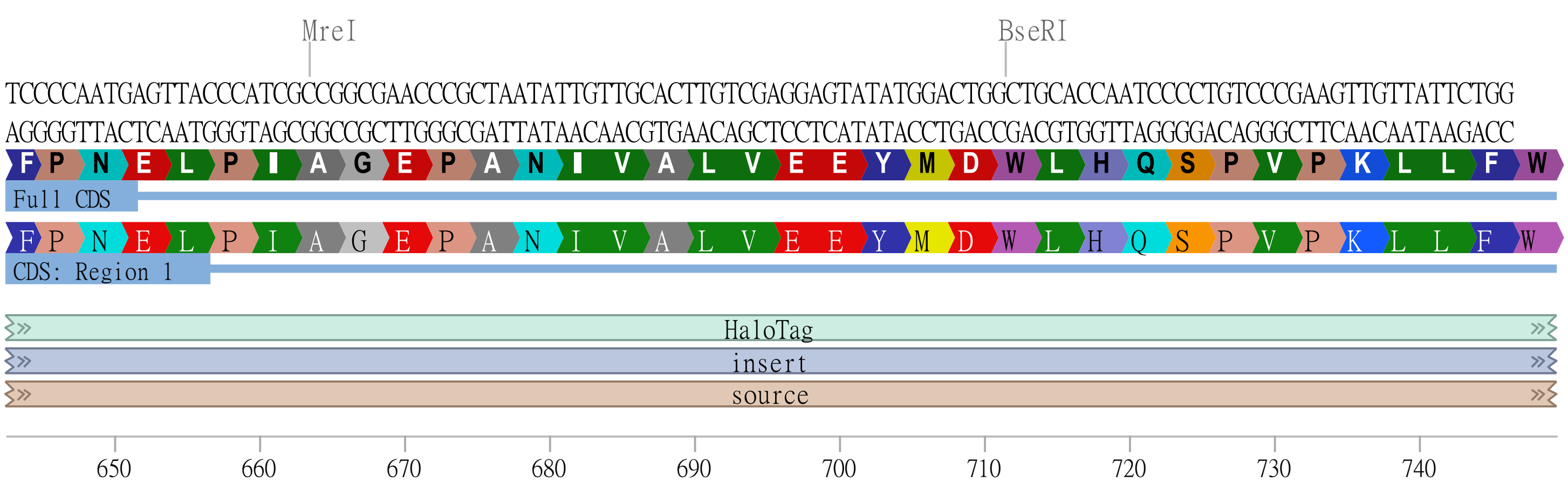

# Halo-sSrc *E. coli* expression construct

Halo_TEV_sSrc in pET-28a(+) (6522 bp) (from 750-1070 bp) 1/23/2025 7:22:27 PM

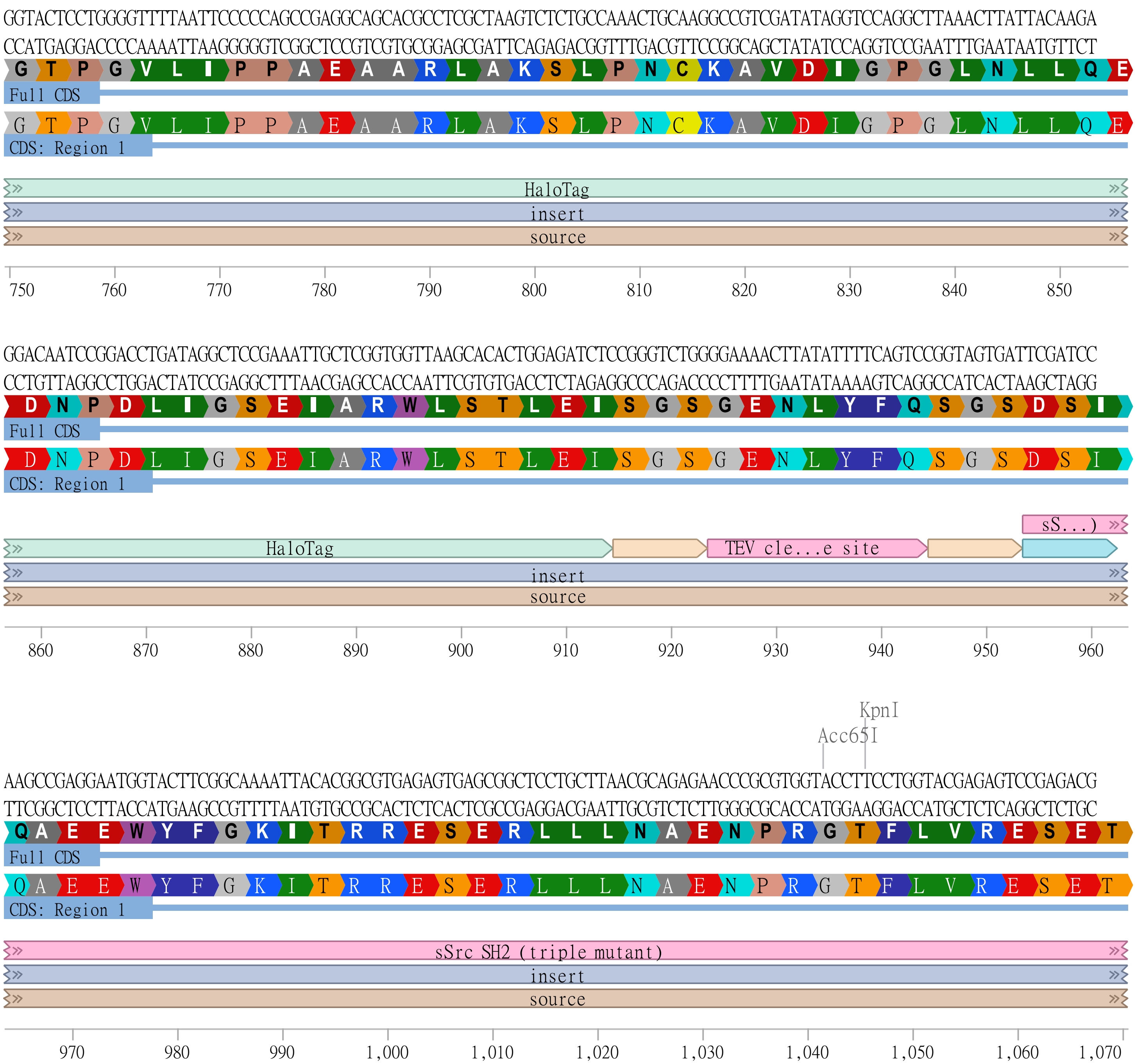

# Halo-sSrc *E. coli* expression construct

Halo_TEV_sSrc in pET-28a(+) (6522 bp) (from 1071-1498 bp)    1/23/2025 7:22:27 PM

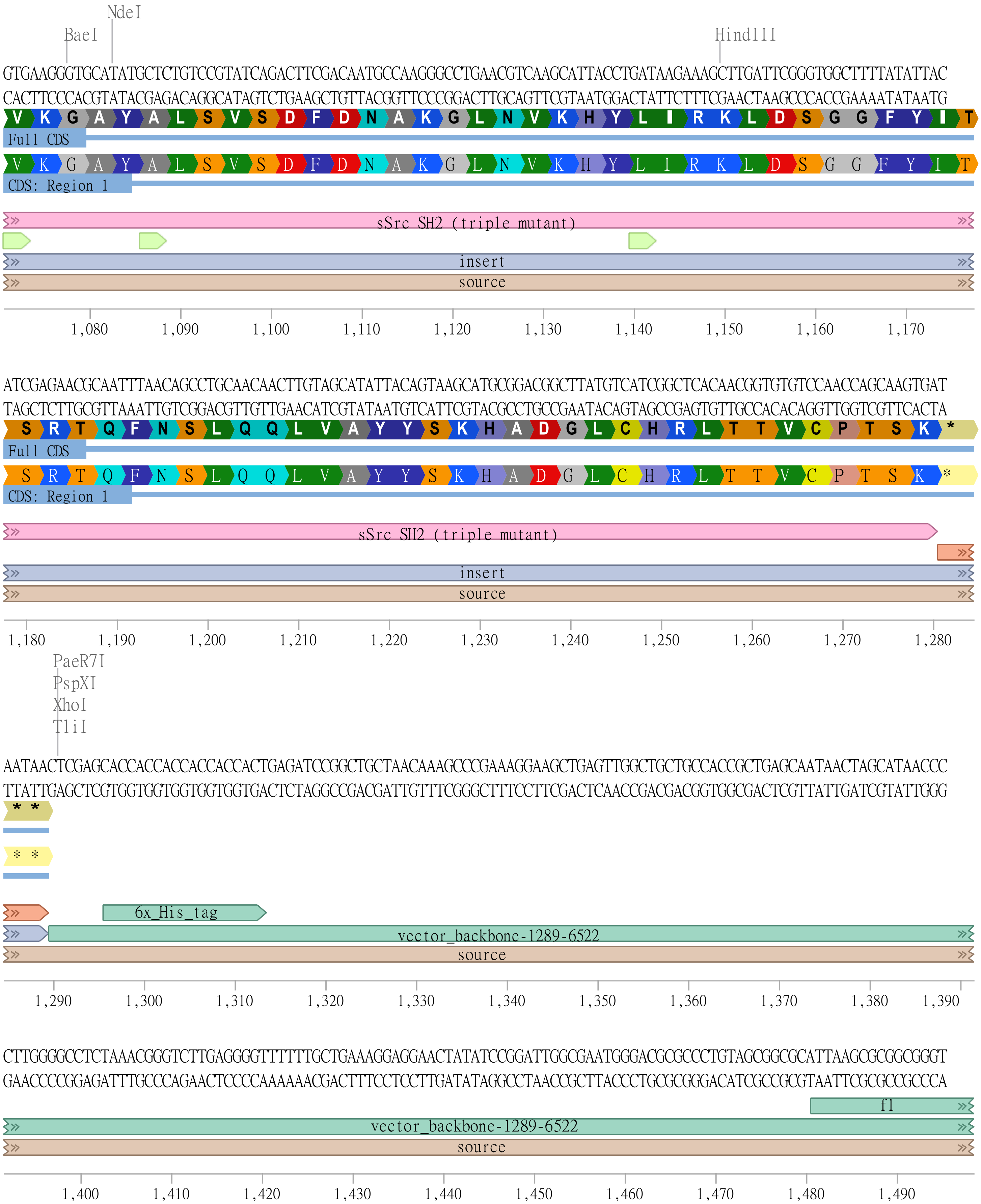

## Halo-sSrc *E. coli* expression construct



GTGGTGGTTACGCGCAGCGTGACCGCTACACTTGCCAGCGCCCTAGCGCCCGCTCCTTTCGCTTTCTTCCCTTCCTTTCTCGCCACGTTCGCCGGCTTTCCCCGTCA
CACCACCAATGCGCGTCGCACTGGCGATGTGAACGGTCGCGGGATCGCGGGCGAGGAAAGCGAAAGAAGGGAAGGAAAGAGCGGTGCAAGCGGCCGAAAGGGGCAGT

f1
vector_backbone-1289-6522
source

1,500 1,510 1,520 1,530 1,540 1,550 1,560 1,570 1,580 1,590 1,600

AGCTCTAAATCGGGGGCTCCCTTTAGGGTTCCGATTTAGTGCTTTACGGCACCTCGACCCCAAAAAACTTGATTAGGGTGATGGTTCACGTAGTGGGCCATCGCCCT
TCGAGATTTAGCCCCCGAGGGAAATCCCAAGGCTAAATCACGAAATGCCGTGGAGCTGGGGTTTTTTGAACTAATCCCACTACCAAGTGCATCACCCGGTAGCGGGA

f1
vector_backbone-1289-6522
source

1,610 1,620 1,630 1,640 1,650 1,660 1,670 1,680 1,690 1,700 1,710

AloI

GATAGACGGTTTTTCGCCCTTTGACGTTGGAGTCCACGTTCTTTAATAGTGGACTCTTGTTCCAAACTGGAACAACACTCAACCCTATCTCGGTCTATTCTTTTGAT
CTATCTGCCAAAAAGCGGGAAACTGCAACCTCAGGTGCAAGAAATTATCACCTGAGAACAAGGTTTGACCTTGTTGTGAGTTGGGATAGAGCCAGATAAGAAAACTA

f1
vector_backbone-1289-6522
source

1,720 1,730 1,740 1,750 1,760 1,770 1,780 1,790 1,800 1,810

PsiI
AanI
CspCI

TTATAAGGGATTTTGCCGATTTCGGCCTATTGGTTAAAAAATGAGCTGATTTAACAAAAATTTAACGCGAATTTTAACAAAATATTAACGCTTACAATTTAGGTGGC
AATATTCCCTAAAACGGCTAAAGCCGGATAACCAATTTTTTACTCGACTAAATTGTTTTTAAATTGCGCTTAAAATTGTTTTATAATTGCGAATGTTAAATCCACCG

vector_backbone-1289-6522
source

1,820 1,830 1,840 1,850 1,860 1,870 1,880 1,890 1,900 1,910 1,920

ACTTTTCGGGGAAATGTGCGCGGAACCCCTATTTGTTTATTTTTCTAAATACATTCAAATATGTATCCGCTCATGAATTAATTCTTAGAAAAACTCATCGAGCATCA
TGAAAAGCCCCTTTACACGCGCCTTGGGGATAAACAAATAAAAAGATTTATGTAAGTTTATACATAGGCGAGTACTTAATTAAGAATCTTTTTGAGTAGCTCGTAGT

kan
vector_backbone-1289-6522
source

1,930 1,940 1,950 1,960 1,970 1,980 1,990 2,000 2,010 2,020 2,030

AATGAAACTGCAATTTATTCATATCAGGATTATCAATACCATATTTTTGAAAAAGCCGTTTCTGTAATGAAGGAGAAAACTCACCGAGGCAGTTCCATAGGATGGCA
TTACTTTGACGTTAAATAAGTATAGTCCTAATAGTTATGGTATAAAAACTTTTTCGGCAAAGACATTACTTCCTCTTTTGAGTGGCTCCGTCAAGGTATCCTACCGT

kan
vector_backbone-1289-6522
source

2,040 2,050 2,060 2,070 2,080 2,090 2,100 2,110 2,120 2,130 2,140

AGATCCTGGTATCGGTCTGCGATTCCGACTCGTCCAACATCAATACAACCTATTAATTTCCCCTCGTCAAAAATAAGGTTATCAAGTGAGAAATCACCATGAGTGAC
TCTAGGACCATAGCCAGACGCTAAGGCTGAGCAGGTTGTAGTTATGTTGGATAATTAAAGGGGAGCAGTTTTTATTCCAATAGTTCACTCTTTAGTGGTACTCACTG

kan
vector_backbone-1289-6522
source

2,150 2,160 2,170 2,180 2,190 2,200 2,210 2,220 2,230 2,240

GACTGAATCCGGTGAGAATGGCAAAAGTTTATGCATTTCTTTCCAGACTTGTTCAACAGGCCAGCCATTACGCTCGTCATCAAAATCACTCGCATCAACCAAACCGT
CTGACTTAGGCCACTCTTACCGTTTTCAAATACGTAAAGAAAGGTCTGAACAAGTTGTCCGGTCGGTAATGCGAGCAGTAGTTTTAGTGAGCGTAGTTGGTTTGGCA

kan
vector_backbone-1289-6522
source

2,250 2,260 2,270 2,280 2,290 2,300 2,310 2,320 2,330 2,340 2,350

SfaAI
AsiSI
SgfI
AjuI PvuI

TATTCATTCGTGATTGCGCCTGAGCGAGACGAAATACGCGATCGCTGTTAAAAGGACAATTACAAACAGGAATCGAATGCAACCGGCGCAGGAACACTGCCAGCGCA
ATAAGTAAGCACTAACGCGGACTCGCTCTGCTTTATGCGCTAGCGACAATTTTCCTGTTAATGTTTGTCCTTAGCTTACGTTGGCCGCGTCCTTGTGACGGTCGCGT

kan
vector_backbone-1289-6522
source

2,360 2,370 2,380 2,390 2,400 2,410 2,420 2,430 2,440 2,450 2,460

SmaI
TspMI
Cfr9I
XmaI

TCAACAATATTTTCACCTGAATCAGGATATTCTTCTAATACCTGGAATGCTGTTTTCCCGGGGATCGCAGTGGTGAGTAACCATGCATCATCAGGAGTACGGATAAA
AGTTGTTATAAAAGTGGACTTAGTCCTATAAGAAGATTATGGACCTTACGACAAAAGGGCCCCTAGCGTCACCACTCATTGGTACGTAGTAGTCCTCATGCCTATTT

kan
vector_backbone-1289-6522
source

2,470 2,480 2,490 2,500 2,510 2,520 2,530 2,540 2,550 2,560

ATGCTTGATGGTCGGAAGAGGCATAAATTCCGTCAGCCAGTTTAGTCTGACCATCTCATCTGTAACATCATTGGCAACGCTACCTTTGCCATGTTTCAGAAACAACT
TACGAACTACCAGCCTTCTCCGTATTTAAGGCAGTCGGTCAAATCAGACTGGTAGAGTAGACATTGTAGTAACCGTTGCGATGGAAACGGTACAAAGTCTTTGTTGA

kan
vector_backbone-1289-6522
source

2,570 2,580 2,590 2,600 2,610 2,620 2,630 2,640 2,650 2,660 2,670

## Halo-sSrc *E. coli* expression construct



Bsp68I
NruI

CTGGCGCATCGGGCTTCCCATACAATCGATAGATTGTCGCACCTGATTGCCCGACATTATCGCGAGCCCATTTATACCCATATAAATCAGCATCCATGTTGGAATTT
GACCGCGTAGCCCGAAGGGTATGTTAGCTATCTAACAGCGTGGACTAACGGGCTGTAATAGCGCTCGGGTAAATATGGGTATATTTAGTCGTAGGTACAACCTTAAA

kan
vector_backbone-1289-6522
source

2,680 2,690 2,700 2,710 2,720 2,730 2,740 2,750 2,760 2,770 2,780

AATCGCGGCCTAGAGCAAGACGTTTCCCGTTGAATATGGCTCATAACACCCCTTGTATTACTGTTTATGTAAGCAGACAGTTTTATTGTTCATGACCAAAATCCCTT
TTAGCGCCGGATCTCGTTCTGCAAAGGGCAACTTATACCGAGTATTGTGGGGAACATAATGACAAATACATTCGTCTGTCAAAATAACAAGTACTGGTTTTAGGGAA

kan
vector_backbone-1289-6522
source

2,790 2,800 2,810 2,820 2,830 2,840 2,850 2,860 2,870 2,880

AACGTGAGTTTTCGTTCCACTGAGCGTCAGACCCCGTAGAAAAGATCAAAGGATCTTCTTGAGATCCTTTTTTTCTGCGCGTAATCTGCTGCTTGCAAACAAAAAAA
TTGCACTCAAAAGCAAGGTGACTCGCAGTCTGGGGCATCTTTTCTAGTTTCCTAGAAGAACTCTAGGAAAAAAAGACGCGCATTAGACGACGAACGTTTGTTTTTTT

colE1_high_copy
vector_backbone-1289-6522
source

2,890 2,900 2,910 2,920 2,930 2,940 2,950 2,960 2,970 2,980 2,990

Eco57I
AcuI

CCACCGCTACCAGCGGTGGTTTGTTTGCCGGATCAAGAGCTACCAACTCTTTTTCCGAAGGTAACTGGCTTCAGCAGAGCGCAGATACCAAATACTGTCCTTCTAGT
GGTGGCGATGGTCGCCACCAAACAAACGGCCTAGTTCTCGATGGTTGAGAAAAAGGCTTCCATTGACCGAAGTCGTCTCGCGTCTATGGTTTATGACAGGAAGATCA

colE1_high_copy
vector_backbone-1289-6522
source

3,000 3,010 3,020 3,030 3,040 3,050 3,060 3,070 3,080 3,090 3,100

AlwNI
CaiI

GTAGCCGTAGTTAGGCCACCACTTCAAGAACTCTGTAGCACCGCCTACATACCTCGCTCTGCTAATCCTGTTACCAGTGGCTGCTGCCAGTGGCGATAAGTCGTGTC
CATCGGCATCAATCCGGTGGTGAAGTTCTTGAGACATCGTGGCGGATGTATGGAGCGAGACGATTAGGACAATGGTCACCGACGACGGTCACCGCTATTCAGCACAG

colE1_high_copy
vector_backbone-1289-6522
source

3,110 3,120 3,130 3,140 3,150 3,160 3,170 3,180 3,190 3,200 3,210

## Halo-sSrc *E. coli* expression construct



```
TTACCGGGTTGGACTCAAGACGATAGTTACCGGATAAGGCGCAGCGGTCGGGCTGAACGGGGGGTTCGTGCACACAGCCCAGCTTGGAGCGAACGACCTACACCGAA
AATGGCCCAACCTGAGTTCTGCTATCAATGGCCTATTCCGCGTCGCCAGCCCGACTTGCCCCCCAAGCACGTGTGTCGGGTCGAACCTCGCTTGCTGGATGTGGCTT
```

colE1_high_copy
vector_backbone-1289-6522
source

3,220 3,230 3,240 3,250 3,260 3,270 3,280 3,290 3,300 3,310

BauI
BssSI
BssSαI

```
CTGAGATACCTACAGCGTGAGCTATGAGAAAGCGCCACGCTTCCCGAAGGGAGAAAGGCGGACAGGTATCCGGTAAGCGGCAGGGTCGGAACAGGAGAGCGCACGAG
GACTCTATGGATGTCGCACTCGATACTCTTTCGCGGTGCGAAGGGCTTCCCTCTTTCCGCCTGTCCATAGGCCATTCGCCGTCCCAGCCTTGTCCTCTCGCGTGCTC
```

colE1_high_copy
vector_backbone-1289-6522
source

3,320 3,330 3,340 3,350 3,360 3,370 3,380 3,390 3,400 3,410 3,420

```
GGAGCTTCCAGGGGGAAACGCCTGGTATCTTTATAGTCCTGTCGGGTTTCGCCACCTCTGACTTGAGCGTCGATTTTTGTGATGCTCGTCAGGGGGGCGGAGCCTAT
CCTCGAAGGTCCCCCTTTGCGGACCATAGAAATATCAGGACAGCCCAAAGCGGTGGAGACTGAACTCGCAGCTAAAAACACTACGAGCAGTCCCCCCGCCTCGGATA
```

colE1_high_copy
vector_backbone-1289-6522
source

3,430 3,440 3,450 3,460 3,470 3,480 3,490 3,500 3,510 3,520 3,530

PscI
PciI

```
GGAAAAACGCCAGCAACGCGGCCTTTTTACGGTTCCTGGCCTTTTGCTGGCCTTTTGCTCACATGTTCTTTCCTGCGTTATCCCCTGATTCTGTGGATAACCGTATT
CCTTTTTGCGGTCGTTGCGCCGGAAAAATGCCAAGGACCGGAAAACGACCGGAAAACGAGTGTACAAGAAAGGACGCAATAGGGGACTAAGACACCTATTGGCATAA
```

vector_backbone-1289-6522
source

3,540 3,550 3,560 3,570 3,580 3,590 3,600 3,610 3,620 3,630

BspQI
SapI
LguI

```
ACCGCCTTTGAGTGAGCTGATACCGCTCGCCGCAGCCGAACGACCGAGCGCAGCGAGTCAGTGAGCGAGGAAGCGGAAGAGCGCCTGATGCGGTATTTTCTCCTTAC
TGGCGGAAACTCACTCGACTATGGCGAGCGGCGTCGGCTTGCTGGCTCGCGTCGCTCAGTCACTCGCTCCTTCGCCTTCTCGCGGACTACGCCATAAAAGAGGAATG
```

vector_backbone-1289-6522
source

3,640 3,650 3,660 3,670 3,680 3,690 3,700 3,710 3,720 3,730 3,740

# Halo-sSrc *E. coli* expression construct

Halo_TEV_sSrc in pET-28a(+) (6522 bp) (from 3746-4387 bp) 1/23/2025 7:22:27 PM

Bst1107I
BstZ17I
PsyI
XmiI
Pf1FI
TatI
AccI
Tth111I

GCATCTGTGCGGTATTTCACACCGCAATGGTGCACTCTCAGTACAATCTGCTCTGATGCCGCATAGTTAAGCCAGTATACACTCCGCTATCGCTACGTGACTGGGTC
CGTAGACACGCCATAAAGTGTGGCGTTACCACGTGAGAGTCATGTTAGACGAGACTACGGCGTATCAATTCGGTCATATGTGAGGCGATAGCGATGCACTGACCCAG

vector_backbone-1289-6522
source

3,750 3,760 3,770 3,780 3,790 3,800 3,810 3,820 3,830 3,840 3,850

ATGGCTGCGCCCCGACACCCGCCAACACCCGCTGACGCGCCCTGACGGGCTTGTCTGCTCCCGGCATCCGCTTACAGACAAGCTGTGACCGTCTCCGGGAGCTGCAT
TACCGACGCGGGGCTGTGGGCGGTTGTGGGCGACTGCGCGGGACTGCCCGAACAGACGAGGGCCGTAGGCGAATGTCTGTTCGACACTGGCAGAGGCCCTCGACGTA

vector_backbone-1289-6522
source

3,860 3,870 3,880 3,890 3,900 3,910 3,920 3,930 3,940 3,950

GTGTCAGAGGTTTTCACCGTCATCACCGAAACGCGCGAGGCAGCTGCGGTAAAGCTCATCAGCGTGGTCGTGAAGCGATTCACAGATGTCTGCCTGTTCATCCGCGT
CACAGTCTCCAAAAGTGGCAGTAGTGGCTTTGCGCGCTCCGTCGACGCCATTTCGAGTAGTCGCACCAGCACTTCGCTAAGTGTCTACAGACGGACAAGTAGGCGCA

ROP
vector_backbone-1289-6522
source

3,960 3,970 3,980 3,990 4,000 4,010 4,020 4,030 4,040 4,050 4,060

CCAGCTCGTTGAGTTTCTCCAGAAGCGTTAATGTCTGGCTTCTGATAAAGCGGGCCATGTTAAGGGCGGTTTTTTCCTGTTTGGTCACTGATGCCTCCGTGTAAGGG
GGTCGAGCAACTCAAAGAGGTCTTCGCAATTACAGACCGAAGACTATTTCGCCCGGTACAATTCCCGCCAAAAAAGGACAAACCAGTGACTACGGAGGCACATTCCC

ROP
vector_backbone-1289-6522
source

4,070 4,080 4,090 4,100 4,110 4,120 4,130 4,140 4,150 4,160 4,170

GGATTTCTGTTCATGGGGGTAATGATACCGATGAAACGAGAGAGGATGCTCACGATACGGGTTACTGATGATGAACATGCCCGGTTACTGGAACGTTGTGAGGGTAA
CCTAAAGACAAGTACCCCCATTACTATGGCTACTTTGCTCTCTCCTACGAGTGCTATGCCCAATGACTACTACTTGTACGGGCCAATGACCTTGCAACACTCCCATT

vector_backbone-1289-6522
source

4,180 4,190 4,200 4,210 4,220 4,230 4,240 4,250 4,260 4,270 4,280

ACAACTGGCGGTATGGATGCGGCGGGACCAGAGAAAAATCACTCAGGGTCAATGCCAGCGCTTCGTTAATACAGATGTAGGTGTTCCACAGGGTAGCCAGCAGCATC
TGTTGACCGCCATACCTACGCCGCCCTGGTCTCTTTTTAGTGAGTCCCAGTTACGGTCGCGAAGCAATTATGTCTACATCCACAAGGTGTCCCATCGGTCGTCGTAG

vector_backbone-1289-6522
source

4,290 4,300 4,310 4,320 4,330 4,340 4,350 4,360 4,370 4,380

# Halo-sSrc *E. coli* expression construct



CTGCGATGCAGATCCGGAACATAATGGTGCAGGGCGCTGACTTCCGCGTTTCCAGACTTTACGAAACACGGAAACCGAAGACCATTCATGTTGTTGCTCAGGTCGCA
GACGCTACGTCTAGGCCTTGTATTACCACGTCCCGCGACTGAAGGCGCAAAGGTCTGAAATGCTTTGTGCCTTTGGCTTCTGGTAAGTACAACAACGAGTCCAGCGT

vector_backbone-1289-6522
source

4,390 4,400 4,410 4,420 4,430 4,440 4,450 4,460 4,470 4,480 4,490

Psp5II
PpuMI

GACGTTTTGCAGCAGCAGTCGCTTCACGTTCGCTCGCGTATCGGTGATTCATTCTGCTAACCAGTAAGGCAACCCCGCCAGCCTAGCCGGGTCCTCAACGACAGGAG
CTGCAAAACGTCGTCGTCAGCGAAGTGCAAGCGAGCGCATAGCCACTAAGTAAGACGATTGGTCATTCCGTTGGGGCGGTCGGATCGGCCCAGGAGTTGCTGTCCTC

vector_backbone-1289-6522
source

4,500 4,510 4,520 4,530 4,540 4,550 4,560 4,570 4,580 4,590 4,600

FspAI
NsbI
FspI

CACGATCATGCGCACCCGTGGGGCCGCCATGCCGGCGATAATGGCCTGCTTCTCGCCGAAACGTTTGGTGGCGGGACCAGTGACGAAGGCTTGAGCGAGGGCGTGCA
GTGCTAGTACGCGTGGGCACCCCGGCGGTACGGCCGCTATTACCGGACGAAGAGCGGCTTTGCAAACCACCGCCCTGGTCACTGCTTCCGAACTCGCTCCCGCACGT

vector_backbone-1289-6522
source

4,610 4,620 4,630 4,640 4,650 4,660 4,670 4,680 4,690 4,700

AGATTCCGAATACCGCAAGCGACAGGCCGATCATCGTCGCGCTCCAGCGAAAGCGGTCCTCGCCGAAAATGACCCAGAGCGCTGCCGGCACCTGTCCTACGAGTTGC
TCTAAGGCTTATGGCGTTCGCTGTCCGGCTAGTAGCAGCGCGAGGTCGCTTTCGCCAGGAGCGGCTTTTACTGGGTCTCGCGACGGCCGTGGACAGGATGCTCAACG

vector_backbone-1289-6522
source

4,710 4,720 4,730 4,740 4,750 4,760 4,770 4,780 4,790 4,800 4,810

PshAI
BoxI

ATGATAAAGAAGACAGTCATAAGTGCGGCGACGATAGTCATGCCCCGCGCCCACCGGAAGGAGCTGACTGGGTTGAAGGCTCTCAAGGGCATCGGTCGAGATCCCGG
TACTATTTCTTCTGTCAGTATTCACGCCGCTGCTATCAGTACGGGGCGCGGGTGGCCTTCCTCGACTGACCCAACTTCCGAGAGTTCCCGTAGCCAGCTCTAGGGCC

vector_backbone-1289-6522
source

4,820 4,830 4,840 4,850 4,860 4,870 4,880 4,890 4,900 4,910 4,920

TGCCTAATGAGTGAGCTAACTTACATTAATTGCGTTGCGCTCACTGCCCGCTTTCCAGTCGGGAAACCTGTCGTGCCAGCTGCATTAATGAATCGGCCAACGCGCGG
ACGGATTACTCACTCGATTGAATGTAATTAACGCAACGCGAGTGACGGGCGAAAGGTCAGCCCTTTGGACAGCACGGTCGACGTAATTACTTAGCCGGTTGCGCGCC

CAP_binding
lacI
vector_backbone-1289-6522
source

4,930 4,940 4,950 4,960 4,970 4,980 4,990 5,000 5,010 5,020

# Halo-sSrc *E. coli* expression construct



GGAGAGGCGGTTTGCGTATTGGGCGCCAGGGTGGTTTTTCTTTTCACCAGTGAGACGGGCAACAGCTGATTGCCCTTCACCGCCTGGCCCTGAGAGAGTTGCAGCAA
CCTCTCCGCCAAACGCATAACCCGCGGTCCCACCAAAAAGAAAAGTGGTCACTCTGCCCGTTGTCGACTAACGGGAAGTGGCGGACCGGGACTCTCTCAACGTCGTT

lacI

vector_backbone-1289-6522

source

5,030 5,040 5,050 5,060 5,070 5,080 5,090 5,100 5,110 5,120 5,130

HincII
KspAI
HpaI

GCGGTCCACGCTGGTTTGCCCCAGCAGGCGAAAATCCTGTTTGATGGTGGTTAACGGCGGGATATAACATGAGCTGTCTTCGGTATCGTCGTATCCCACTACCGAGA
CGCCAGGTGCGACCAAACGGGGTCGTCCGCTTTTAGGACAAACTACCACCAATTGCCGCCCTATATTGTACTCGACAGAAGCCATAGCAGCATAGGGTGATGGCTCT

lacI

vector_backbone-1289-6522

source

5,140 5,150 5,160 5,170 5,180 5,190 5,200 5,210 5,220 5,230 5,240

Eco32I
EcoRV

BssHII
PauI

TATCCGCACCAACGCGCAGCCCGGACTCGGTAATGGCGCGCATTGCGCCCAGCGCCATCTGATCGTTGGCAACCAGCATCGCAGTGGGAACGATGCCCTCATTCAGC
ATAGGCGTGGTTGCGCGTCGGGCCTGAGCCATTACCGCGCGTAACGCGGGTCGCGGTAGACTAGCAACCGTTGGTCGTAGCGTCACCCTTGCTACGGGAGTAAGTCG

lacI

vector_backbone-1289-6522

source

5,250 5,260 5,270 5,280 5,290 5,300 5,310 5,320 5,330 5,340 5,350

ATTTGCATGGTTTGTTGAAAACCGGACATGGCACTCCAGTCGCCTTCCCGTTCCGCTATCGGCTGAATTTGATTGCGAGTGAGATATTTATGCCAGCCAGCCAGACG
TAAACGTACCAAACAACTTTTGGCCTGTACCGTGAGGTCAGCGGAAGGGCAAGGCGATAGCCGACTTAAACTAACGCTCACTCTATAAATACGGTCGGTCGGTCTGC

lacI

vector_backbone-1289-6522

source

5,360 5,370 5,380 5,390 5,400 5,410 5,420 5,430 5,440 5,450

ApaI
Bsp120I
PspOMI

CAGACGCGCCGAGACAGAACTTAATGGGCCCGCTAACAGCGCGATTTGCTGGTGACCCAATGCGACCAGATGCTCCACGCCCAGTCGCGTACCGTCTTCATGGGAGA
GTCTGCGCGGCTCTGTCTTGAATTACCCGGGCGATTGTCGCGCTAAACGACCACTGGGTTACGCTGGTCTACGAGGTGCGGGTCAGCGCATGGCAGAAGTACCCTCT

lacI

vector_backbone-1289-6522

source

5,460 5,470 5,480 5,490 5,500 5,510 5,520 5,530 5,540 5,550 5,560

# Halo-sSrc *E. coli* expression construct

Halo_TEV_sSrc in pET-28a(+) (6522 bp) (from 5565-6206 bp) 1/23/2025 7:22:27 PM

AAATAATACTGTTGATGGGTGTCTGGTCAGAGACATCAAGAAATAACGCCGGAACATTAGTGCAGGCAGCTTCCACAGCAATGGCATCCTGGTCATCCAGCGGATAG
TTTATTATGACAACTACCCACAGACCAGTCTCTGTAGTTCTTTATTGCGGCCTTGTAATCACGTCCGTCGAAGGTGTCGTTACCGTAGGACCAGTAGGTCGCCTATC

lacI
vector_backbone-1289-6522
source

5,570 5,580 5,590 5,600 5,610 5,620 5,630 5,640 5,650 5,660 5,670

BclI

TTAATGATCAGCCCACTGACGCGTTGCGCGAGAAGATTGTGCACCGCCGCTTTACAGGCTTCGACGCCGCTTCGTTCTACCATCGACACCACCACGCTGGCACCCAG
AATTACTAGTCGGGTGACTGCGCAACGCGCTCTTCTAACACGTGGCGGCGAAATGTCCGAAGCTGCGGCGAAGCAAGATGGTAGCTGTGGTGGTGCGACCGTGGGTC

lacI
vector_backbone-1289-6522
source

5,680 5,690 5,700 5,710 5,720 5,730 5,740 5,750 5,760 5,770

TTGATCGGCGCGAGATTTAATCGCCGCGACAATTTGCGACGGCGCGTGCAGGGCCAGACTGGAGGTGGCAACGCCAATCAGCAACGACTGTTTGCCCGCCAGTTGTT
AACTAGCCGCGCTCTAAATTAGCGGCGCTGTTAAACGCTGCCGCGCACGTCCCGGTCTGACCTCCACCGTTGCGGTTAGTCGTTGCTGACAAACGGGCGGTCAACAA

lacI
vector_backbone-1289-6522
source

5,780 5,790 5,800 5,810 5,820 5,830 5,840 5,850 5,860 5,870 5,880

GTGCCACGCGGTTGGGAATGTAATTCAGCTCCGCCATCGCCGCTTCCACTTTTTCCCGCGTTTTCGCAGAAACGTGGCTGGCCTGGTTCACCACGCGGGAAACGGTC
CACGGTGCGCCAACCCTTACATTAAGTCGAGGCGGTAGCGGCGAAGGTGAAAAAGGGCGCAAAAGCGTCTTTGCACCGACCGGACCAAGTGGTGCGCCCTTTGCCAG

lacI
vector_backbone-1289-6522
source

5,890 5,900 5,910 5,920 5,930 5,940 5,950 5,960 5,970 5,980 5,990

BstAPI

TGATAAGAGACACCGGCATACTCTGCGACATCGTATAACGTTACTGGTTTCACATTCACCACCCTGAATTGACTCTCTTCCGGGCGCTATCATGCCATACCGCGAAA
ACTATTCTCTGTGGCCGTATGAGACGCTGTAGCATATTGCAATGACCAAAGTGTAAGTGGTGGGACTTAACTGAGAGAAGGCCCGCGATAGTACGGTATGGCGCTTT

lacIQ_promoter
lacI
vector_backbone-1289-6522
source

6,000 6,010 6,020 6,030 6,040 6,050 6,060 6,070 6,080 6,090

GGTTTTGCGCCATTCGATGGTGTCCGGGATCTCGACGCTCTCCCTTATGCGACTCCTGCATTAGGAAGCAGCCCAGTAGTAGGTTGAGGCCGTTGAGCACCGCCGCC
CCAAAACGCGGTAAGCTACCACAGGCCCTAGAGCTGCGAGAGGGAATACGCTGAGGACGTAATCCTTCGTCGGGTCATCATCCAACTCCGGCAACTCGTGGCGGCGG

lacIQ_promoter
vector_backbone-1289-6522
source

6,100 6,110 6,120 6,130 6,140 6,150 6,160 6,170 6,180 6,190 6,200

GCAAGGAATGGTGCATGCAAGGAGATGGCGCCCAACAGTCCCCCGGCCACGGGGCCTGCCACCATACCCACGCCGAAACAAGCGCTCATGAGCCCGAAGTGGCGAGC
CGTTCCTTACCACGTACGTTCCTCTACCGCGGGTTGTCAGGGGGCCGGTGCCCCGGACGGTGGTATGGGTGCGGCTTTGTTCGCGAGTACTCGGGCTTCACCGCTCG

vector_backbone-1289-6522

source

6,210 6,220 6,230 6,240 6,250 6,260 6,270 6,280 6,290 6,300 6,310

CCGATCTTCCCCATCGGTGATGTCGGCGATATAGGCGCCAGCAACCGCACCTGTGGCGCCGGTGATGCCGGCCACGATGCGTCCGGCGTAGAGGATCGAGATCTCGA
GGCTAGAAGGGGTAGCCACTACAGCCGCTATATCCGCGGTCGTTGGCGTGGACACCGCGGCCACTACGGCCGGTGCTACGCAGGCCGCATCTCCTAGCTCTAGAGCT

vector_backbone-1289-6522

source

6,320 6,330 6,340 6,350 6,360 6,370 6,380 6,390 6,400 6,410 6,420

XbaI NcoI

TCCCGCGAAATTAATACGACTCACTATAGGGGAATTGTGAGCGGATAACAATTCCCCTCTAGAAATAATTTTGTTTAACTTTAAGAAGGAGATATACCATGG
AGGGCGCTTTAATTATGCTGAGTGATATCCCCTTAACACTCGCCTATTGTTAAGGGGAGATCTTTATTAAAACAAATTGAAATTCTTCCTCTATATGGTACC

M

M

lacO T7_tr..._RBS

T7 RBS_3

vector_backbone-1289-6522

source

6,430 6,440 6,450 6,460 6,470 6,480 6,490 6,500 6,510 6,520

**Appendix Figure S18. Annotated Spectra of Novel pY Sites**
**[see following pages]**

## GRB10 Y15 | z=4 scan=21264 EGF15min

XCorr=3.83 Ascore=46.7 ppm=9.1 | b/y cov=30% pY–imm=YES NL–98=YES site–diag=3 | [Ac]–ALAGCPDSFLHHPpYYQDKVEQTPR

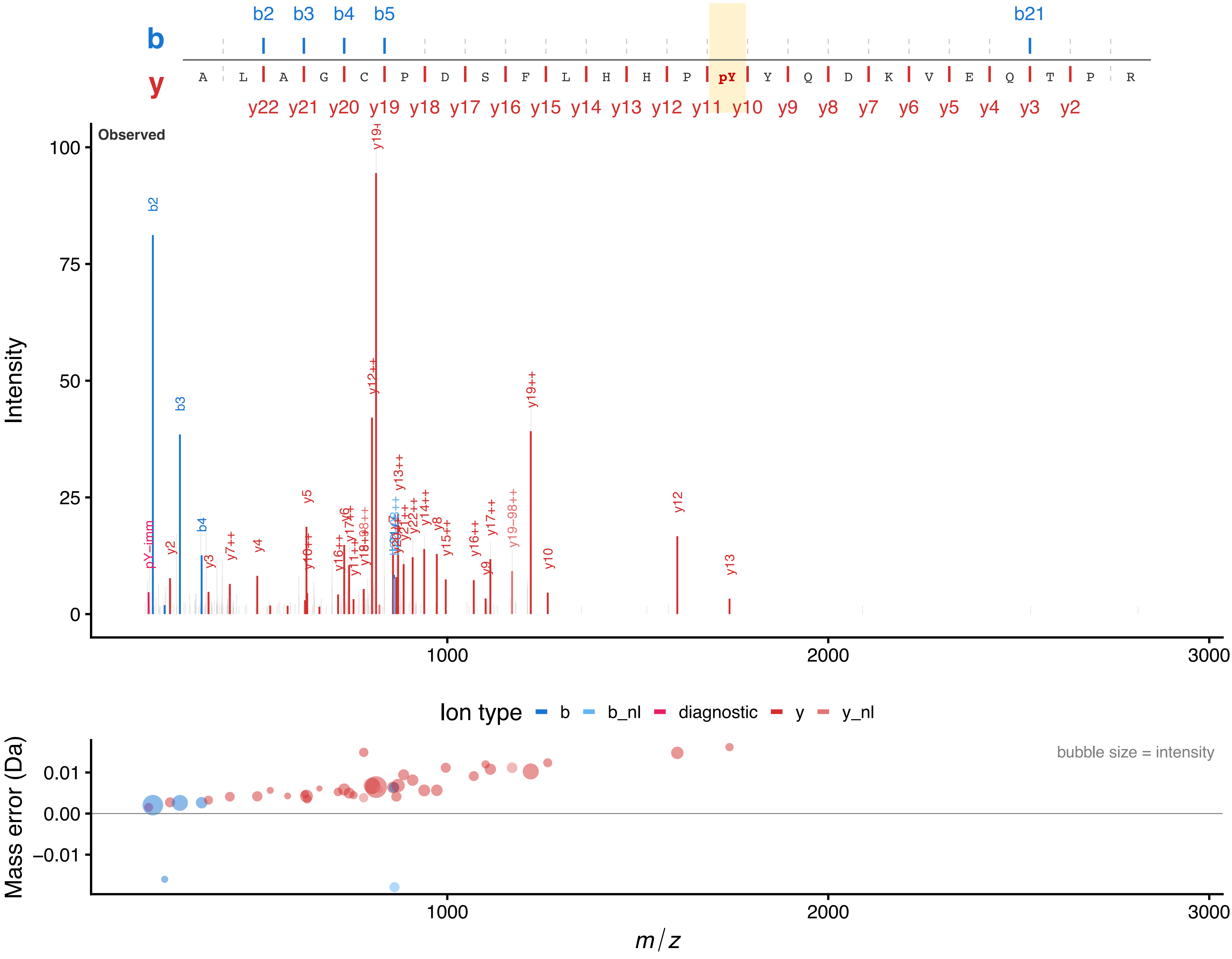


ProForma: <[+57.021]@C>[+42.011]-ALAGCPDSFLHHPY[+79.966]YQDKVEQTPR/4

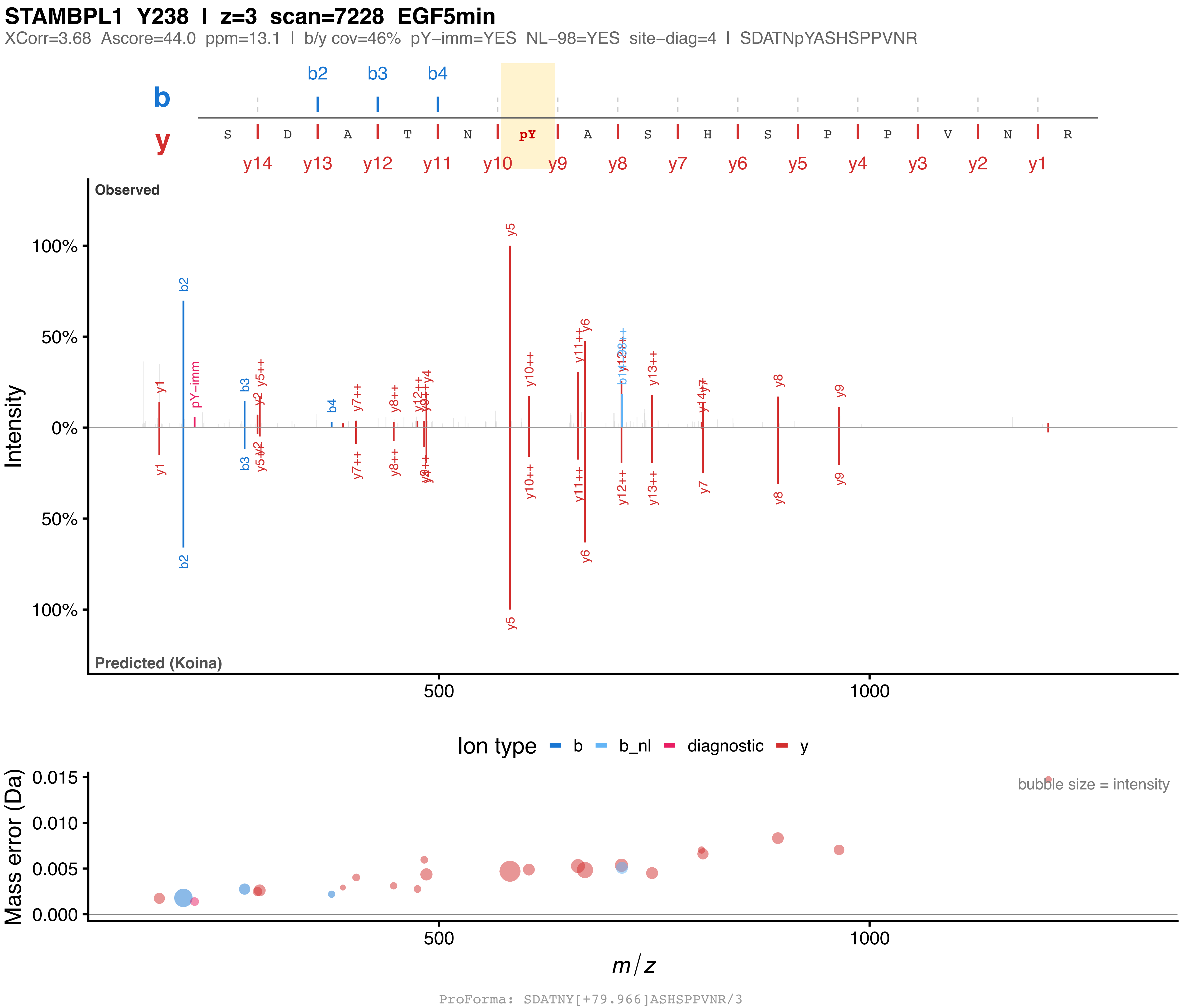
STAMBPL1 Y238 | z=3 scan=7228 EGF5min
XCorr=3.68 Ascore=44.0 ppm=13.1 | b/y cov=46% pY−imm=YES NL−98=YES site−diag=4 | SDATNpYASHSPPVNR
b
y
S D A T N pY A S H S P P V N R
b2 b3 b4
y14 y13 y12 y11 y10 y9 y8 y7 y6 y5 y4 y3 y2 y1
Observed
Predicted (Koina)
Intensity
100%
50%
0%
Ion type b b_nl diagnostic y
Mass error (Da)
0.015
0.010
0.005
0.000
bubble size = intensity
500
1000
m/z
ProForma: SDATNY[+79.966]ASHSPPVNR/3

**PLCG2 Y13 | z=3 scan=19710 EGF1min**

XCorr=3.27 Ascore=48.6 ppm=7.6 | b/y cov=39% pY−imm=YES NL−98=YES site−diag=3 | [Ac]−STTVNVDSLAEpYEKSQIKR

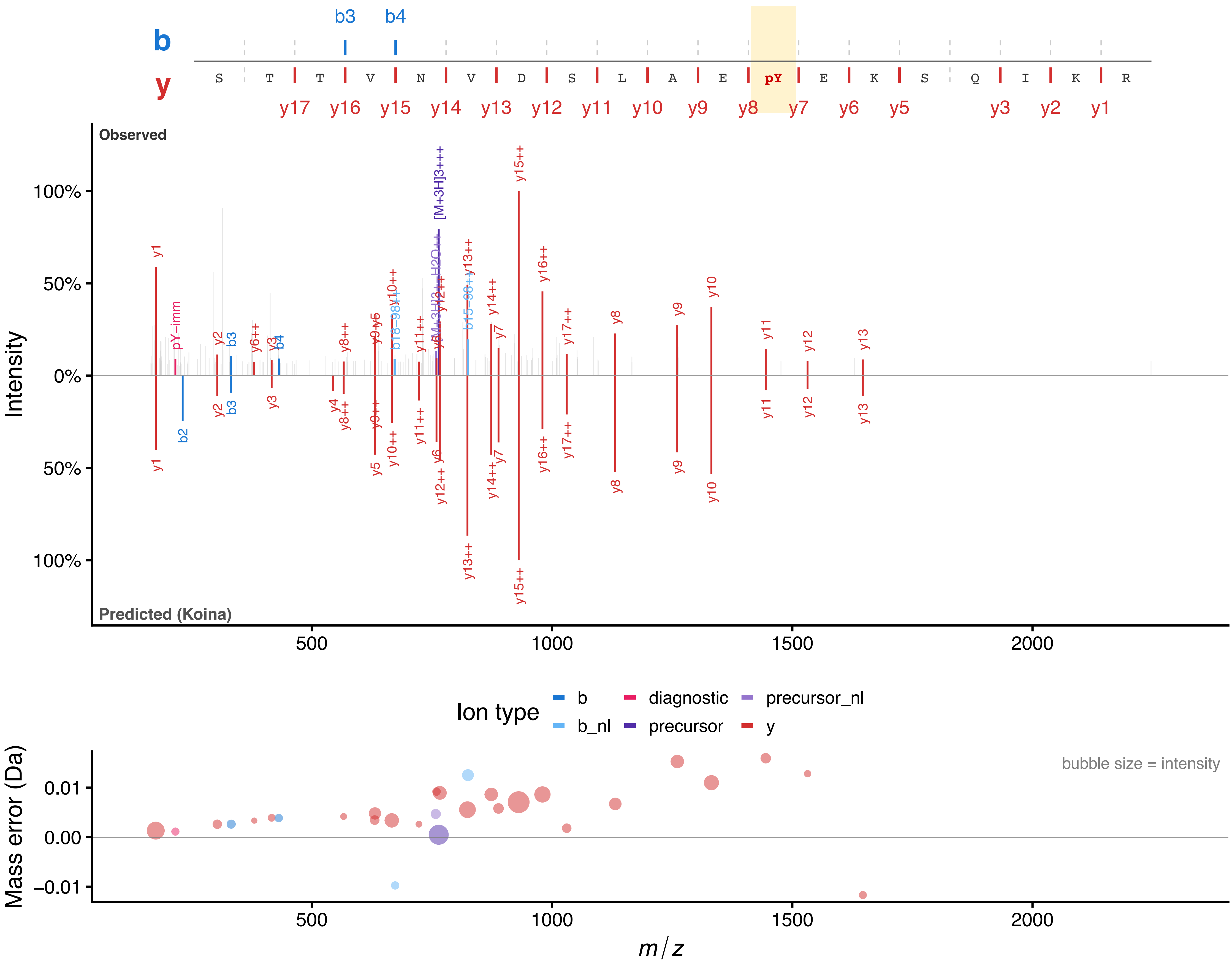


ProForma: [+42.011]-STTVNVDSLAEY[+79.966]EKSQIKR/3

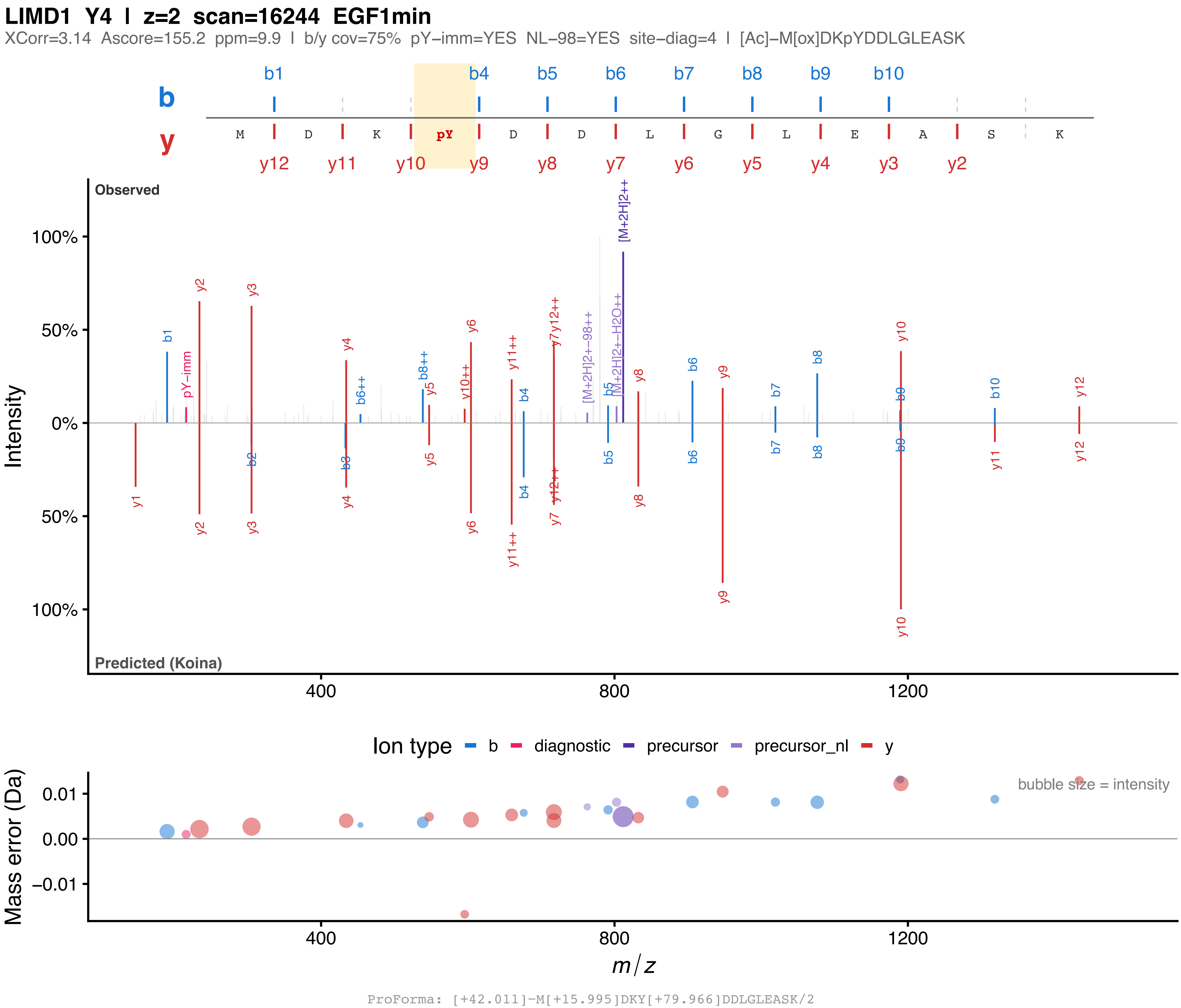

LIMD1 Y4 | z=2 scan=16244 EGF1min
XCorr=3.14 Ascore=155.2 ppm=9.9 | b/y cov=75% pY−imm=YES NL−98=YES site−diag=4 | [Ac]−M[ox]DKpYDDLGLEASK
b
y
M D K pY D D L G L E A S K
Observed
Predicted (Koina)
Intensity
m/z
Ion type b diagnostic precursor precursor_nl y
Mass error (Da)
bubble size = intensity
ProForma: [+42.011]-M[+15.995]DKY[+79.966]DDLGLEASK/2

# STX12 Y9 | z=3 scan=20766 EGF5min

XCorr=2.84 Ascore=88.8 ppm=11.5 | b/y cov=28% pY−imm=YES NL−98=no site−diag=2 | [Ac]−SYGPLDM[ox]pYRNPGPSGPQLR

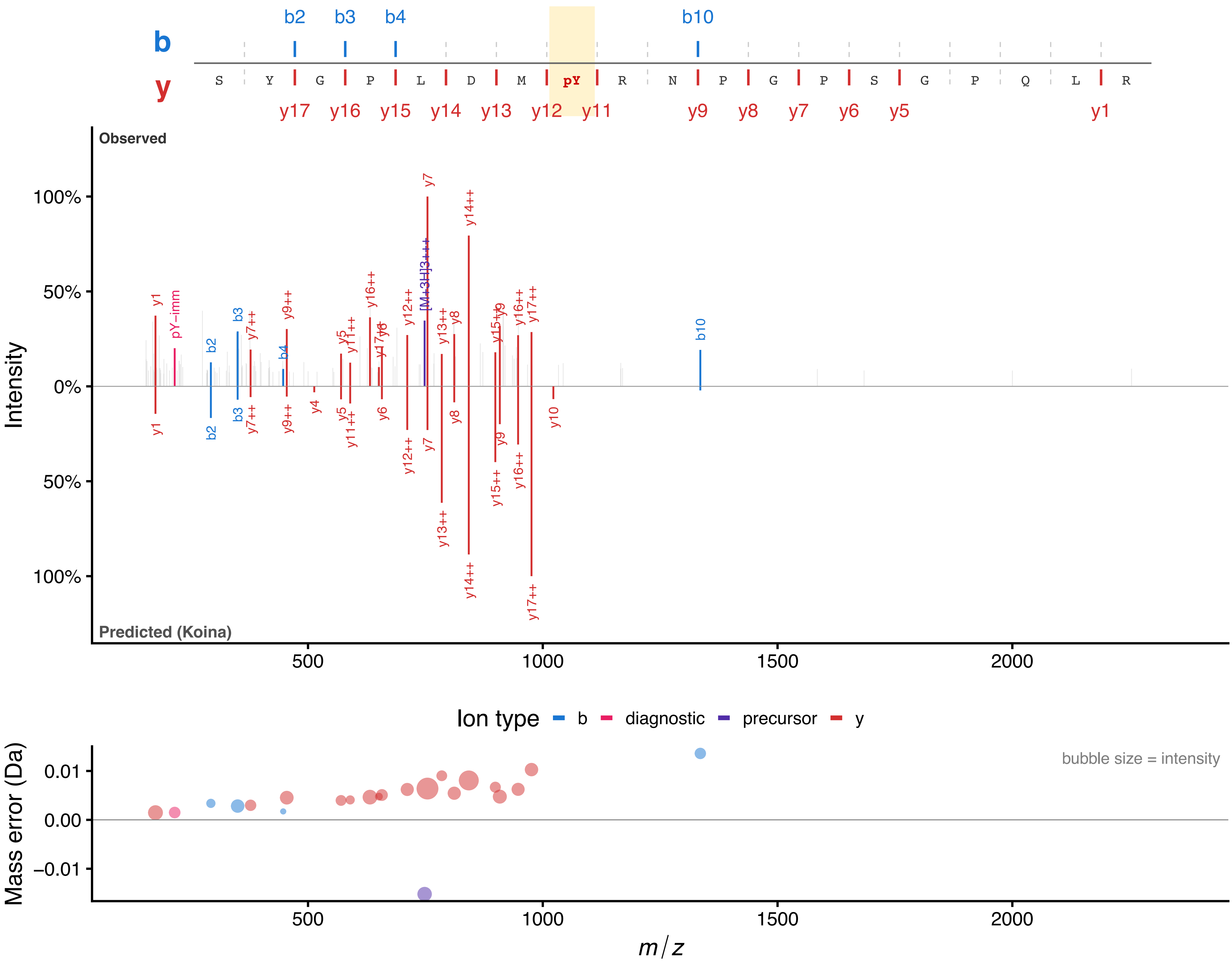


ProForma: [+42.011]−SYGPLDM[+15.995]Y[+79.966]RNPGPSGPQLR/3

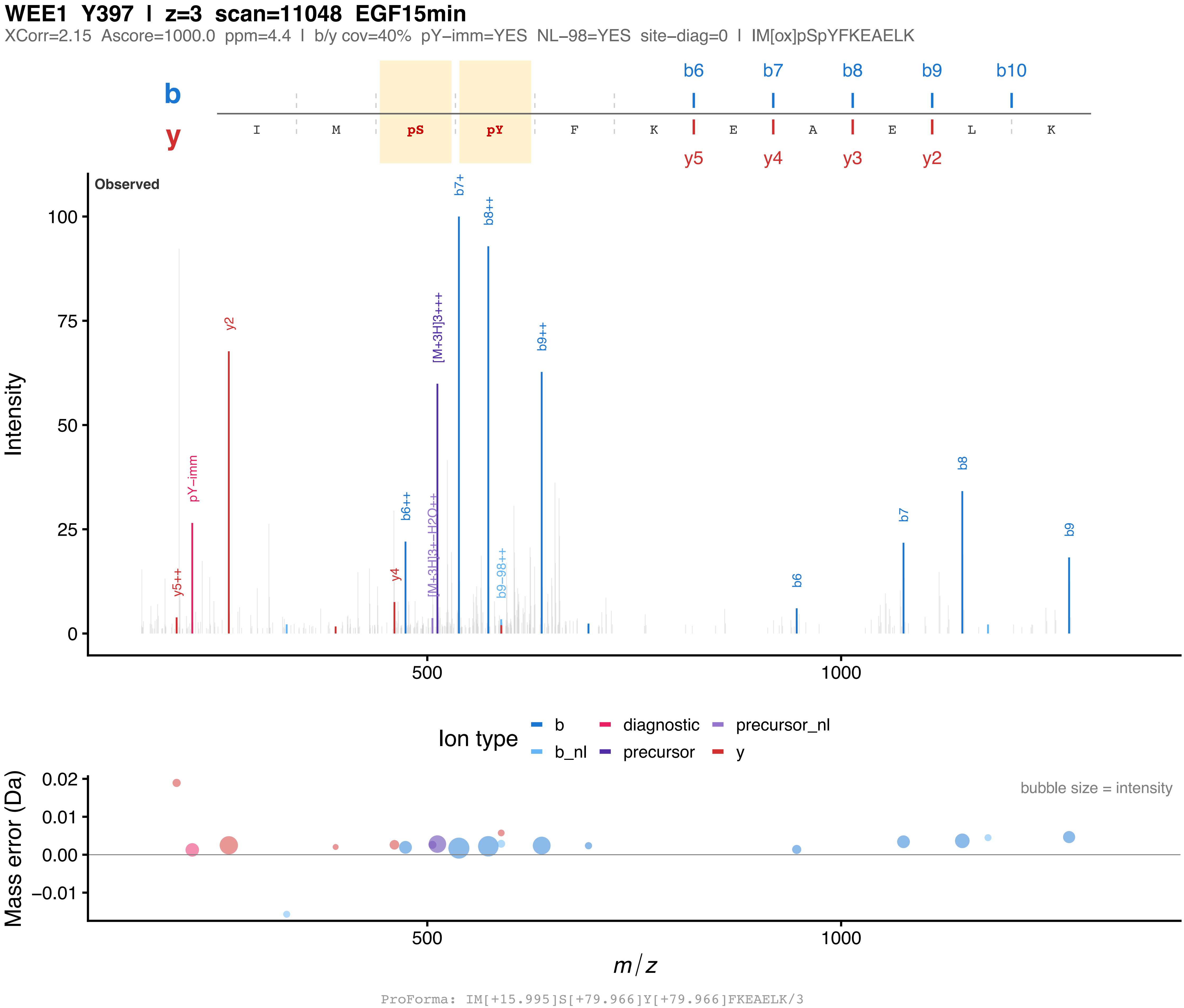

WEE1 Y397 | z=3 scan=11048 EGF15min
XCorr=2.15 Ascore=1000.0 ppm=4.4 | b/y cov=40% pY−imm=YES NL−98=YES site−diag=0 | IM[ox]pSpYFKEAELK
b
y
I M pS pY F K E A E L K
b6 b7 b8 b9 b10
y5 y4 y3 y2
Observed
Intensity
b7+
b8++
y2
[M+3H]3+++
b9++
pY−imm
b6++
[M+3H]3+−H2O++
y5++
y4
b9−98++
b6
b7
b8
b9
Ion type
b
b_nl
diagnostic
precursor
precursor_nl
y
Mass error (Da)
bubble size = intensity
m/z
ProForma: IM[+15.995]S[+79.966]Y[+79.966]FKEAELK/3

**SUMO1 Y21 | z=2 scan=11243 EGF5min**

XCorr=1.95 Ascore=1000.0 ppm=10.1 | b/y cov=79% pY−imm=YES NL−98=YES site−diag=4 | EGEpYIKLK

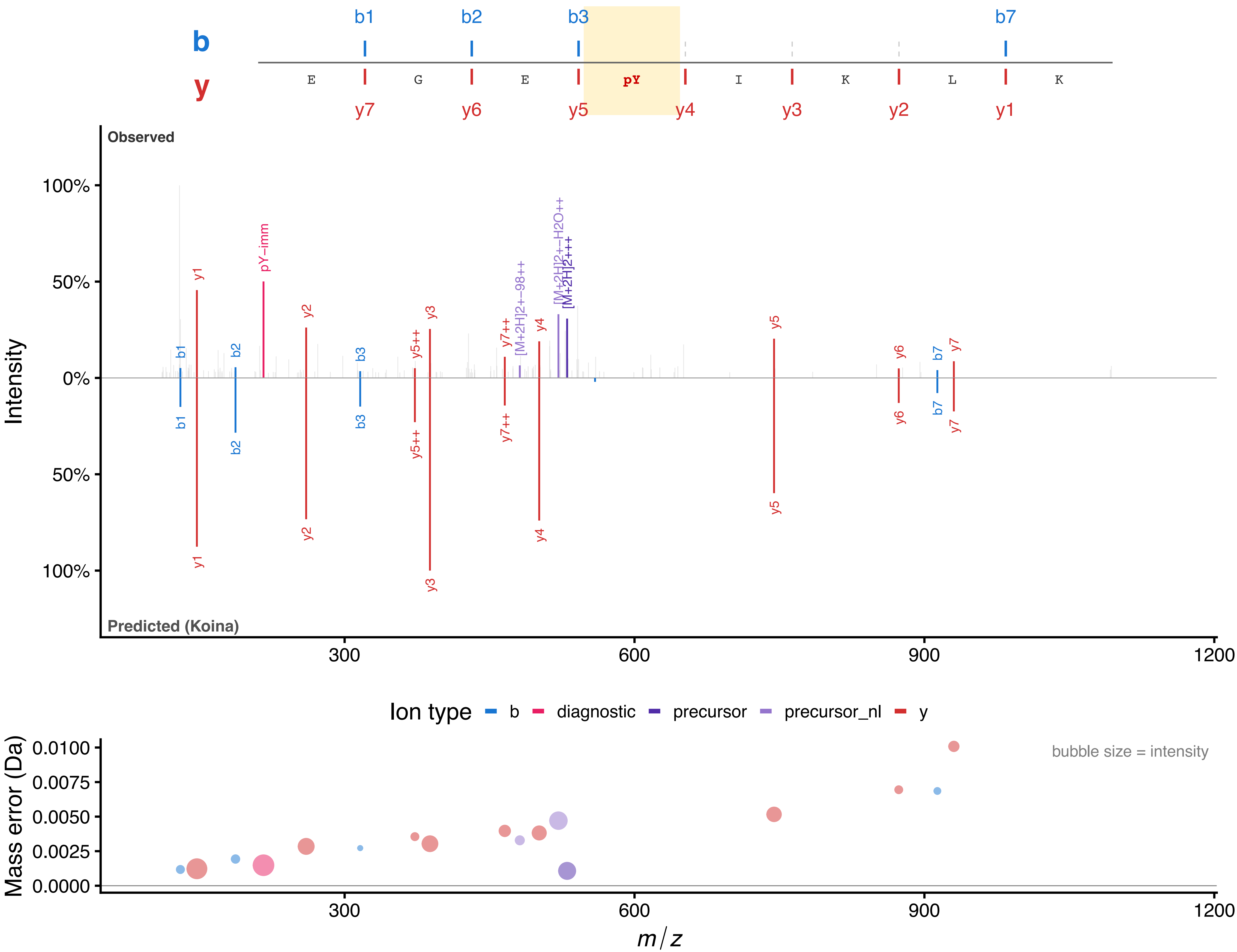


ProForma: EGEY[+79.966]IKLK/2

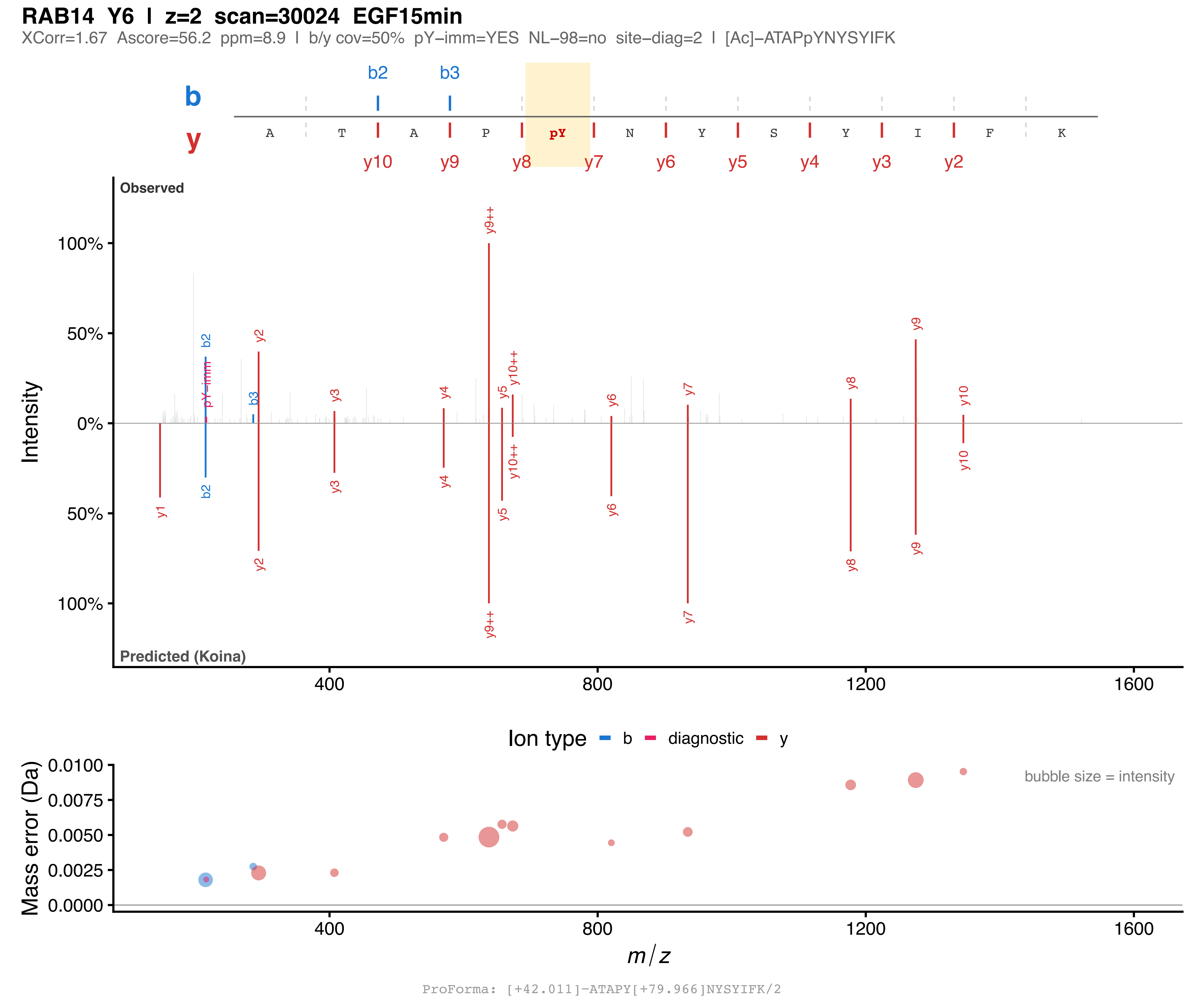
RAB14 Y6 | z=2 scan=30024 EGF15min
XCorr=1.67 Ascore=56.2 ppm=8.9 | b/y cov=50% pY−imm=YES NL−98=no site−diag=2 | [Ac]−ATAPpYNYSYIFK
b
y
b2
b3
A T A P pY N Y S Y I F K
y10 y9 y8 y7 y6 y5 y4 y3 y2
Observed
Predicted (Koina)
Intensity
100%
50%
0%
50%
100%
400
800
1200
1600
y9++
y10++
y1
y2
y3
y4
y5
y6
y7
y8
y9
y10
pY−imm
Ion type
b
diagnostic
y
Mass error (Da)
0.0100
0.0075
0.0050
0.0025
0.0000
bubble size = intensity
m/z
ProForma: [+42.011]−ATAPY[+79.966]NYSYIFK/2

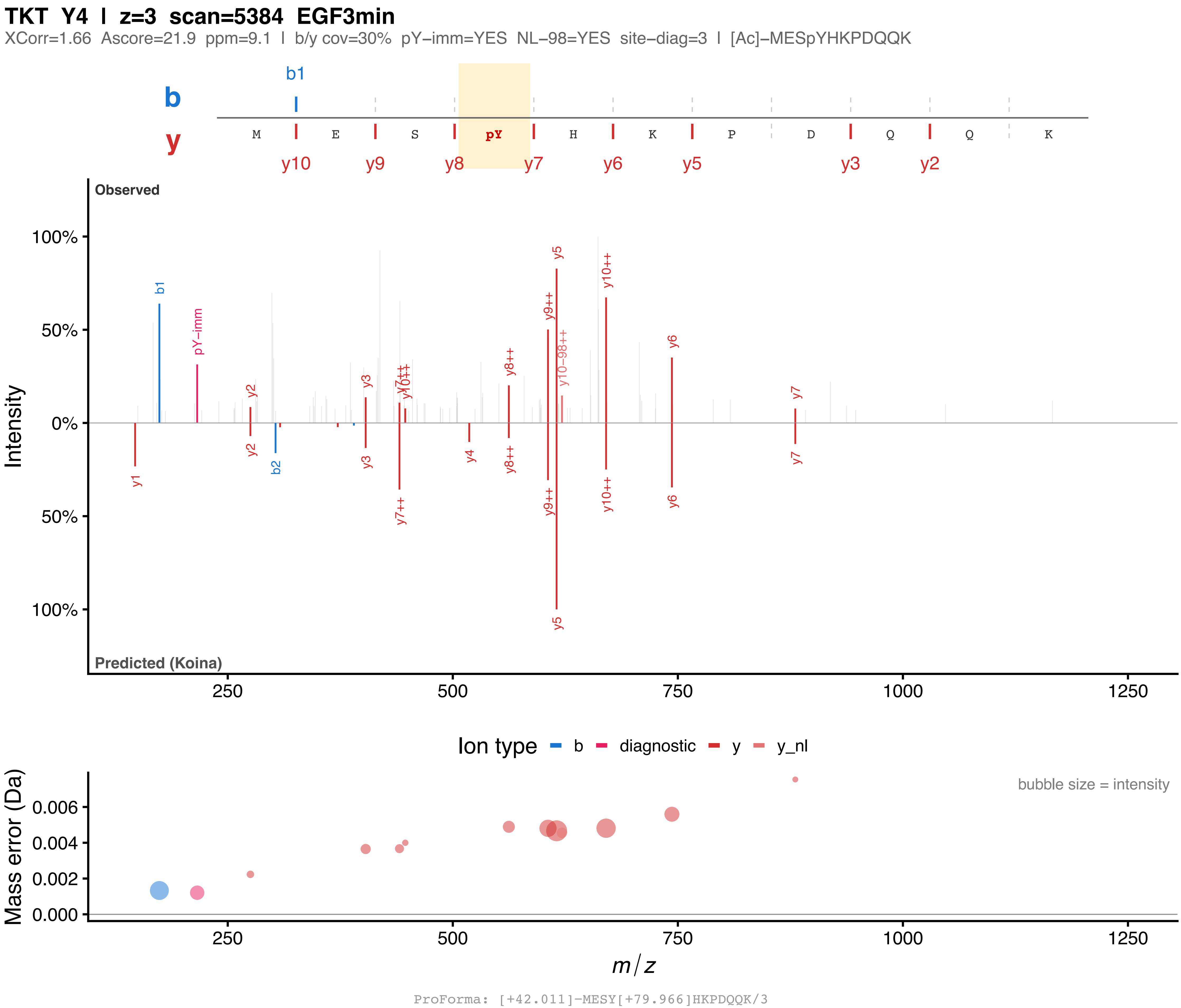

TKT Y4 | z=3 scan=5384 EGF3min
XCorr=1.66 Ascore=21.9 ppm=9.1 | b/y cov=30% pY−imm=YES NL−98=YES site−diag=3 | [Ac]−MESpYHKPDQQK
b
y
b1
M E S pY H K P D Q Q K
y10 y9 y8 y7 y6 y5 y3 y2
Observed
Predicted (Koina)
Intensity
Ion type b diagnostic y y_nl
Mass error (Da)
bubble size = intensity
m/z
ProForma: [+42.011]−MESY[+79.966]HKPDQQK/3

**RNF126 Y27 | z=2 scan=16825 EGF15min**

XCorr=1.50 Ascore=1000.0 ppm=8.6 | b/y cov=71% pY−imm=YES NL−98=YES site−diag=5 | LPDpYICPR

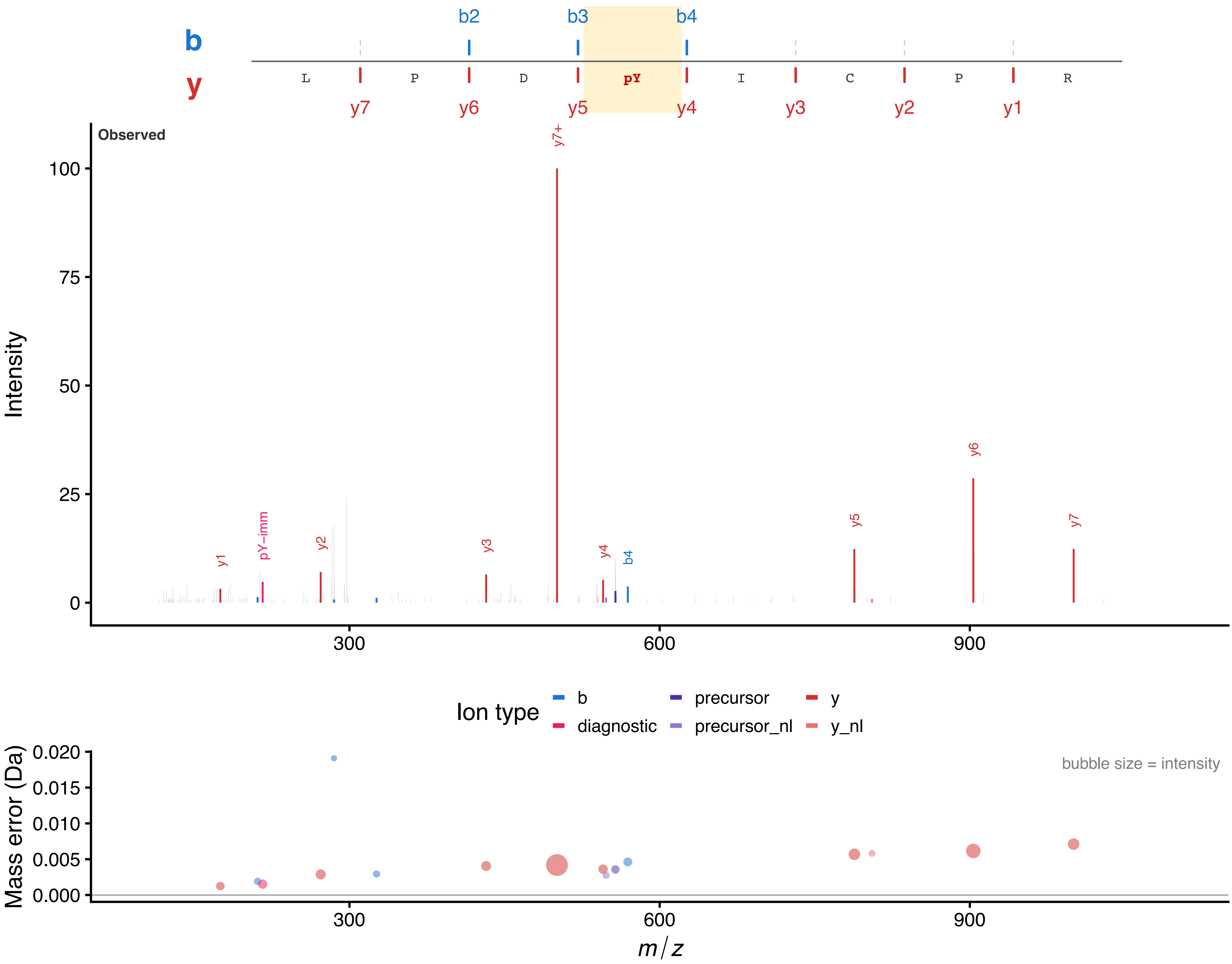


ProForma: <[+57.021]@C>LPDY[+79.966]ICPR/2

**RAD18 Y117 | z=2 scan=8050 EGF5min**

XCorr=1.50 Ascore=68.0 ppm=13.9 | b/y cov=57% pY−imm=YES NL−98=no site−diag=3 | VpYTPVASR

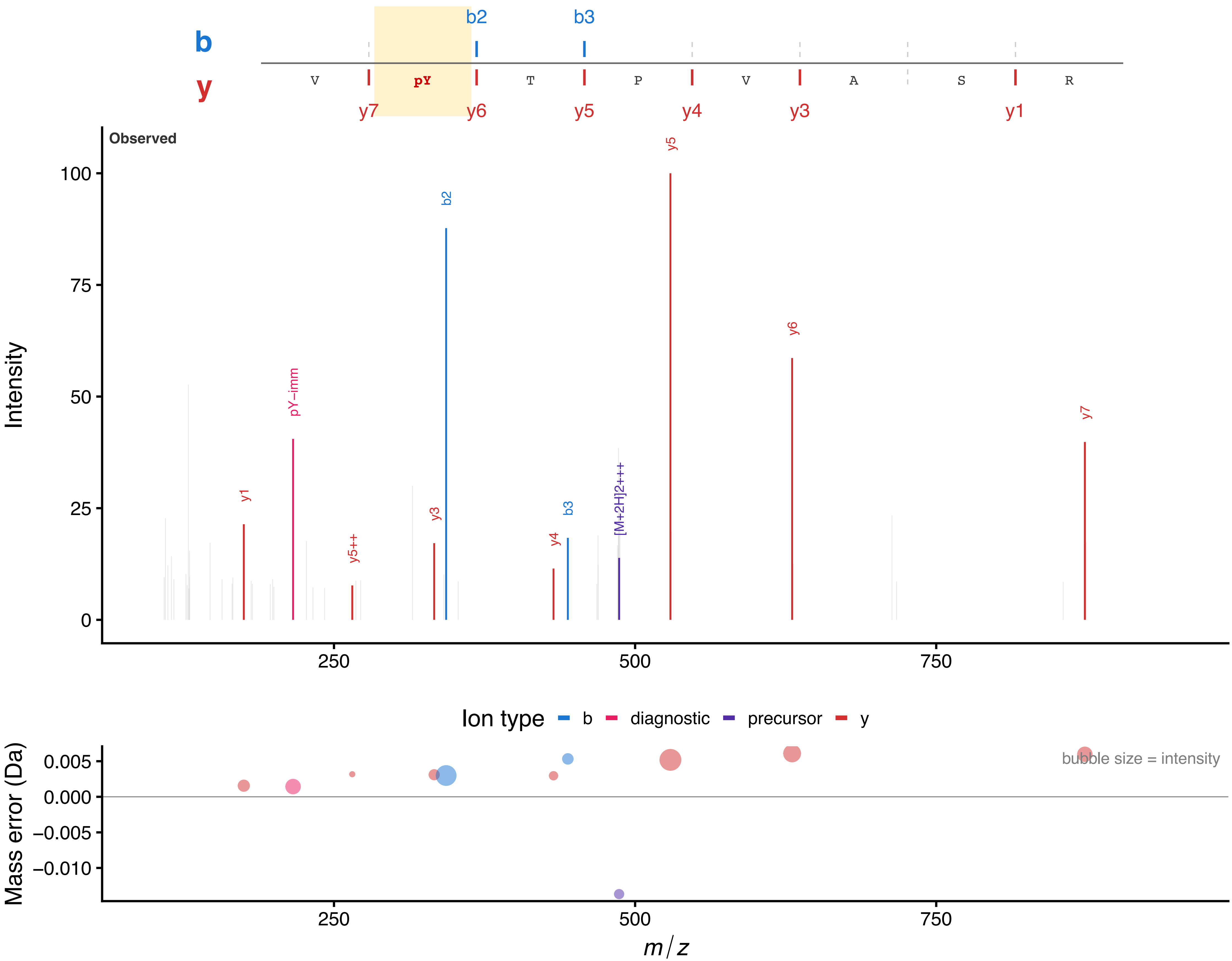


ProForma: VY[+79.966]TPVASR/2

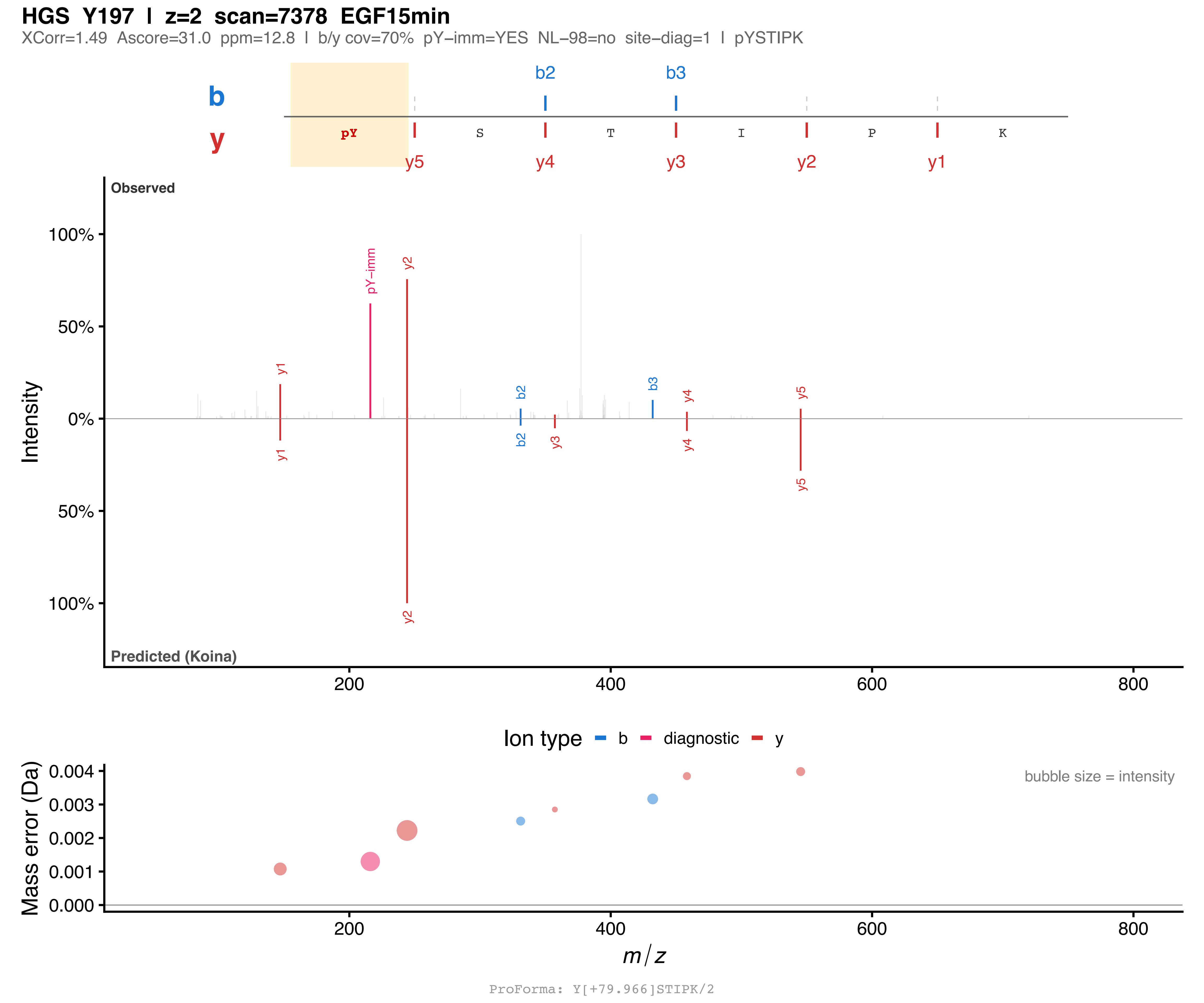

HGS Y197 | z=2 scan=7378 EGF15min
XCorr=1.49 Ascore=31.0 ppm=12.8 | b/y cov=70% pY−imm=YES NL−98=no site−diag=1 | pYSTIPK
b
y
b2
b3
pY
S
T
I
P
K
y5
y4
y3
y2
y1
Observed
Predicted (Koina)
Intensity
100%
50%
0%
pY−imm
Ion type
b
diagnostic
y
Mass error (Da)
bubble size = intensity
m/z
ProForma: Y[+79.966]STIPK/2
