# Supplementary material for: Scalable phosphotyrosine enrichment with SH2 superbinder enables deep profiling of EGF responses
Source: EMBO J. 2026 Jul 3;45(15):5556–79. doi: 10.1038/s44318-026-00843-8 (PMC13434655; doi:10.1038/s44318-026-00843-8)
Supplement: Supplementary file 2 [file 44318_2026_843_MOESM2_ESM.pdf]

THE EMBO JOURNAL

Scalable Phosphotyrosine Enrichment with SH2 Superbinder Enables Deep Profiling of EGF Responses

Alexis Chang, Ricard Rodriguez-Mias, Matthew Berg, Sophie Moggridge, and Judit Villen

Corresponding author(s): Judit Villen (jvillen@uw.edu)

Review Timeline:

Submission Date:	30th Apr 26
Editorial Decision:	22nd May 26
Revision Received:	11th Jun 26
Accepted:	15th Jun 26

Editor: Hartmut Vodermaier

Transaction Report:

(Note: With the exception of the correction of typographical or spelling errors that could be a source of ambiguity, letters and reports are not edited. Depending on transfer agreements, referee reports obtained elsewhere may or may not be included in this compilation. Referee reports are anonymous unless the Referee chooses to sign their reports.)

This manuscript was transferred to The EMBO JOURNAL following peer review at another journal.

Point-by-Point Response to Reviewers

Phosphotyrosine Proteomics Using a Scalable SH2 Superbinder Enrichment Strategy

Chang et al.

We thank the reviewers for their constructive evaluation. Their feedback has strengthened the manuscript, and we have addressed each point. Below we provide our responses (black) to each reviewer comment (blue) with manuscript revisions flagged in **red**. Major additions include:

(1) a new opening Results section illustrating how phosphotyrosine proteomics studies are stalled and motivating the need for accessible methods. Specifically, we show the scale disparity between tyrosine- and global-phosphoproteomic studies in PhosphoSitePlus and ProteomeExchange;

(2) an explicit transitive benchmark of Halo-sSrc against the CST PTMScan pY antibody, anchored in our 2023 JPR head-to-head comparison;

(3) manually annotated MS/MS spectra and false discovery rate (FDR) analysis for novel pY site identifications;

(4) revised framing of biological observations as hypothesis-generating rather than mechanistic; and

(5) revisions throughout for clarity and to address specific technical questions.

General Editorial Concerns

> You will see that, while the reviewers find your work of interest, they raise substantive concerns that cast doubt on the advance your findings represent over earlier work and the strength of the novel conclusions that can be drawn at this stage. Unfortunately, these reservations are sufficiently important to preclude publication of this study in this journal.

We acknowledge that phosphotyrosine enrichment methods using SH2 superbinders or antibodies have been out for a while. However, phosphotyrosine proteomics has not yet been broadly applicable due to the high cost, intensive hands-on time, limited scalability, and specialized protein purification expertise required for previous enrichment methods. We believe the reviewers' assessment of our method overlooks the impact of democratizing technology, which in our opinion is the main success of a method. This is precisely what our method accomplishes for phosphotyrosine proteomics and will enable a broad range of new discoveries.

To show the little progress in phosphotyrosine proteomics we have included a new section and figure at the beginning of the Results, where we explore the scale and depth of publicly available phosphotyrosine studies and datasets, in comparison to global phosphoproteomics:

First, experimental evidence for human pY sites in PhosphoSitePlus is dominated by internal Cell Signaling Technologies (CST) studies. CST has been able to contribute massive amounts of phosphotyrosine observations as they do not face the same cost and infrastructure barriers as their customers. Of the known pY sites validated by at least three independent studies, only 24% (3,167 sites) derive from external studies (Figure 1A). In contrast, 75% (46,972 sites) of triply validated pS or pT sites derive from external studies. This disparity suggests researchers have readily adopted global phosphoproteomic methods but not tyrosine phosphoproteomic methods.

Second, analysis of ProteomeExchange deposits from 2015-2025 shows tyrosine-specific studies average 15 samples per study, versus 169 for global phosphoproteomics – a ~10-fold gap (Figure 1B). The only pY study exceeding 40 enrichments was from a developer of the superbinder reagent itself (Mirhadi *et al*, 2022; Martyn *et al*, 2022; Tong *et al*, 2017), underscoring that external adoption plateaus well below the sample counts now standard for global phosphoproteomics.

The method presented in our manuscript (R2HaPpY) eliminates these barriers with a 20-fold reduction in cost and preparation time. The impact of enabling technologies is best measured by the discoveries

they make possible across the community, not solely by the biological follow-up in the introducing paper.

Action: Added a new opening Results section ("Current tyrosine phosphoproteomics methods are not being scaled up") and Figure 1 quantifying the community scale gap. Added explicit transitive benchmarking statement, FDR analysis, and new Appendix Figures with annotated spectra for novel pY sites.

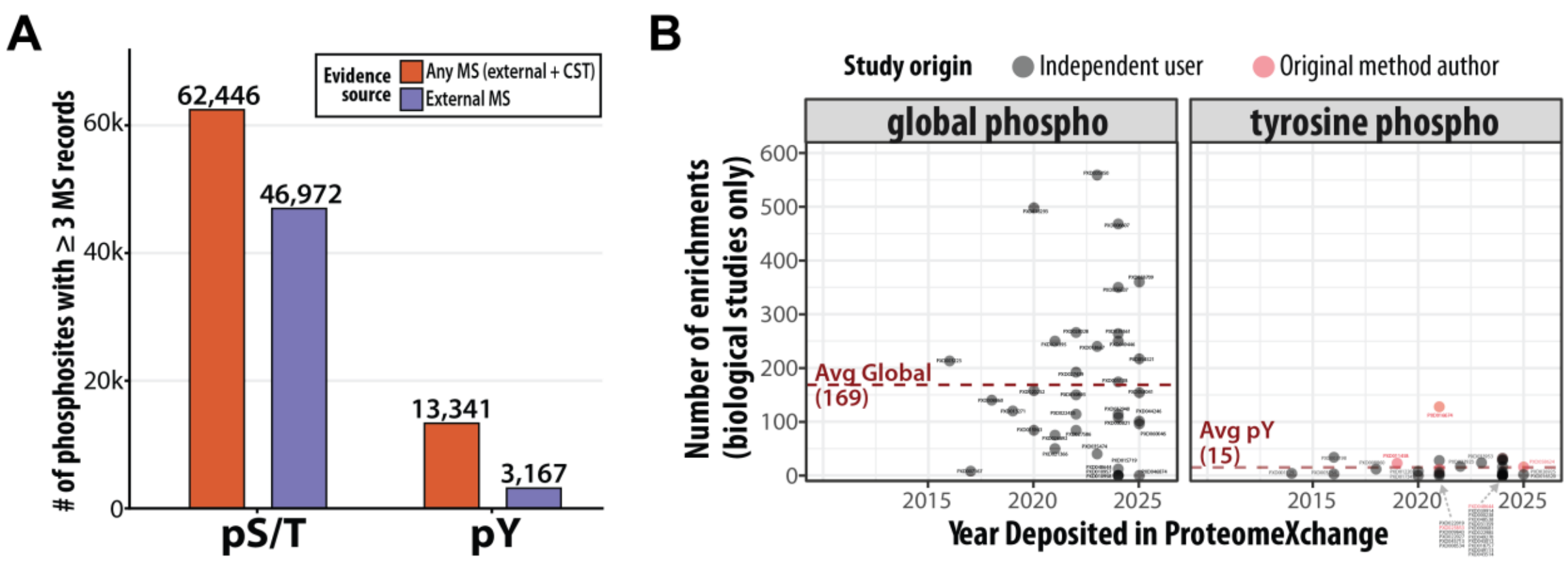

Review Figure 1. Tyrosine phosphoproteomics remains bottlenecked.

A. Evidence source distribution for human phosphorylation sites in PhosphoSitePlus with at least 3 high-throughput mass spectrometry observations, separated by phospho-acceptor residue. Orange indicates sites evidenced by either internal Cell Signaling Technologies (CST) studies or external literature. Purple indicates sites evidenced by at least three external studies. Phosphoserine and phosphothreonine sites are evidenced by a higher percentage of externally backed studies highlighting the difference in adoption of global phospho and tyrosine-specific phosphopeptide studies.

B. Dot plot indicates numbers of enrichments (y-axis) for studies deposited in ProteomeExchange between 2015 to December 2025 (x-axis) that utilized global (left) or tyrosine (right) phosphoproteomics approaches. Points are labelled by ProteomeExchange experiment accession. Studies were identified by keywords ‘phosphoproteomics’ and/or ‘tyrosine’. Associated publications were ranked by raw file count then manually inspected to identify number of enrichments performed for global and tyrosine specific phosphoproteomes using either Src SH2 superbinder or anti-phosphotyrosine antibody. Red lines indicate average enrichment numbers for global phosphoproteome and tyrosine phosphoproteomes. Enrichments of global phosphoproteome utilized on average 10-times more samples than for tyrosine phosphoproteomes underscoring barrier for scaling phosphotyrosine studies.

Reviewer #1

Point 1.1: Incremental advancement

> The manuscript is well written and the results are convincing. The method will be of interest to proteomics practitioners and those interested in protein-pY modifications in particular. Overall, the results represent an incremental advancement of the sSH2-based approach, which dozens of publications have demonstrated.

We thank the reviewer for the positive overall assessment and respectfully disagree with the characterization as incremental. The critical distinction is between demonstrating a concept and making it practical. Prior sSH2 approaches required multi-day protein purification, buffer exchange, and multi-step conjugation — each demanding specialized equipment and expertise. R2HaPpY replaces that entire pipeline with a single HaloTag capture step directly from crude lysate, reducing bead preparation from 3 days to 1-2 hours, lowering cost per sample 20-fold, and integrating with automated 96-well enrichment. This is not parameter optimization within an existing workflow; it is a re-engineered workflow.

The practical impact is visible in the community data: the only pY enrichment study with ≥100 samples is from a laboratory that developed one of the original sSH2 approaches (Mirhadi *et al*, 2022; Tong *et al*, 2017; Martyn *et al*, 2022). Outside of method-developing labs, the largest pY study is 34 samples (Humphrey *et al*, 2016); and the remaining high-sample-count pY enrichments were performed internally by CST with proprietary antibodies (Figure 1). Over the same timeframe, global phosphopeptide enrichments outpace pY by roughly 10-fold. Despite a decade of the initial sSH2-based publication and two decades of antibody-based approach, adoption has been gated by cost and effort — exactly the barriers R2HaPpY removes. Since posting our preprint, we have received inquiries from multiple laboratories seeking to implement the method. Commercial interest from Promega and the recently released Axobind superbinder product provides further evidence that R2HaPpY addresses an unmet need.

Point 1.2: Claims of new insights into EGFR signaling are speculative

> The claims of new insights into EGFR signaling are speculative at best and not tested, for example by characterization of Y-to-F variants.

We agree that Y-to-F validation is the gold standard for establishing site function, and we agree that our biological observations are hypothesis-generating rather than mechanistically validated. Our claim is narrower than the review implies: we identify pY sites on EGF-response proteins that are positioned — structurally and evolutionarily — to modulate established mechanisms (ubiquitin recognition by RNF126, PI3P binding by HGS, or kinase regulation of WEE1). One highlighted site, TKT Y4, has functional support from a prior study of a Y-to-A mutant (Qin *et al*, 2019). These are testable mechanistic hypotheses grounded in structure, not claims of validated function, and they illustrate the starting points that a deeper pY dataset enables. In the original version of the manuscript, we already stated that *"the involvement of these novel sites within EGF signaling is <u>currently speculative</u> but demonstrate how a deep systems-level analysis of pY signaling using our method can uncover new, <u>potentially functionally relevant</u> pY sites and <u>can drive hypothesis generation for further experiments.</u>"*

To build confidence in the novel-site identifications themselves, we have added manually annotated MS/MS spectra for a subset of high-interest novel pY sites and a detailed FDR analysis (Appendix File), confirming these identifications pass stringent quality thresholds.

Action: Added FDR analysis to Results and manually annotated MS/MS spectra for novel pY sites to Appendix File.

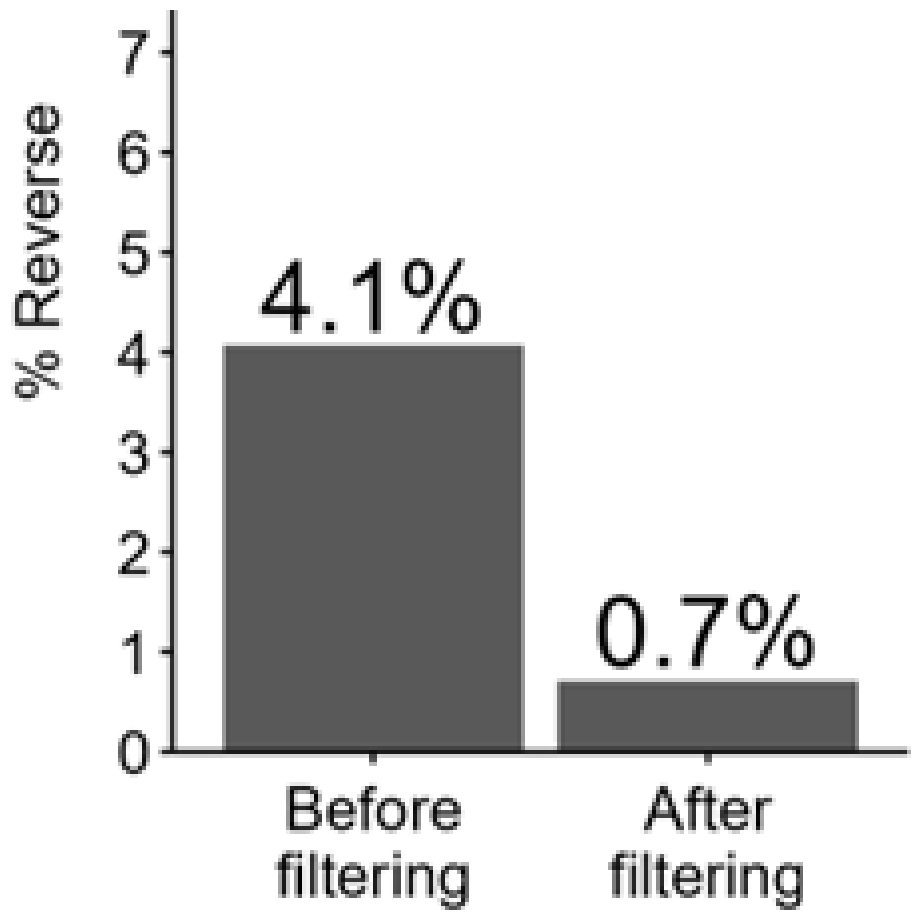

Review Figure 2. False discovery rate of pY sites is controlled by completeness filter.

False detection rate (FDR) estimation was assessed by target-decoy approach both before and after filtering for pY sites present in at least 50% of replicates of minimum one condition within the EGF stimulation time course. In both cases, pY sites were already filtered for confident localization by requiring Ascore ≥ 13. FDR was calculated as counts of pY sites on decoy peptides divided by counts of all peptides (target plus decoy peptides) then converted to percentage. This low FDR adds confidence to the pY sites reported in the EGF stimulation experiment.

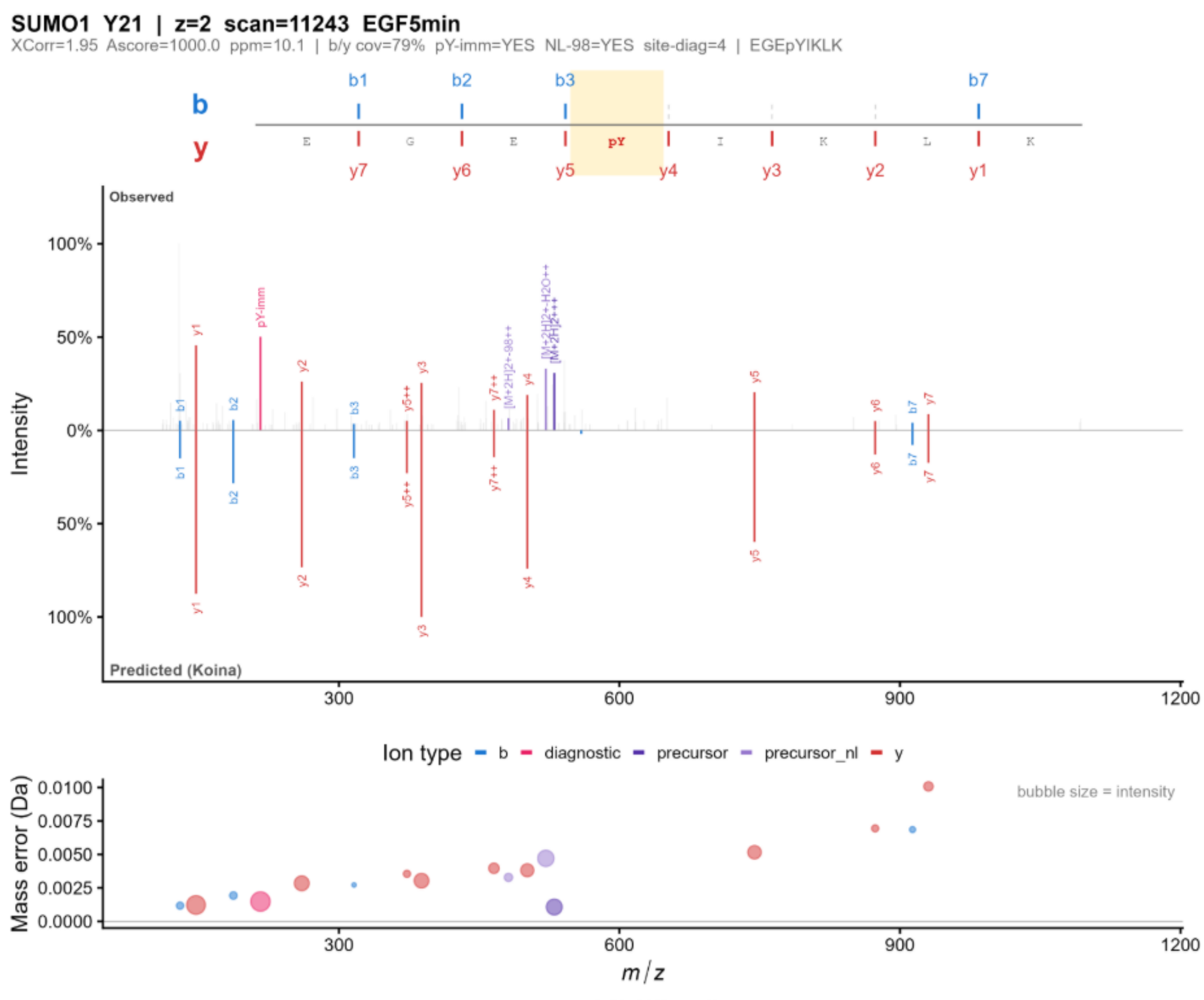

Review Figure 3. Example of an annotated spectra of novel pY sites identified as EGF regulated.
Representative spectra for each site were annotated for ions: b, y, neutral loss, and phosphotyrosine immonium ions using a custom R script based on spectrum-utils (Bittremieux *et al*, 2023; Bittremieux, 2020). When possible, predicted spectra from AlphaPeptDeep (Zeng *et al*, 2022) accessed via Koina (Lautenbacher *et al*, 2025) server are provided for reference in a mirror plot. Additional spectra are included in the Appendix File.

Point 1.3: Contradictory statement about accessibility

> The authors state “However, sSrc bead preparation is intensive and not accessible to most proteomics labs, involving affinity purification of sSrc protein, buffer exchange, and multi-step conjugation; which hinders wide adoption of the approach28–31,33–37.” However, the statement contradicts itself by reference to the various papers that have documented the application of various sSH2 approaches to pY characterization.

We agree that the original phrasing was imprecise. The cited publications demonstrate that the sSH2 approach works, but they do not demonstrate broad adoption — in fact, only one study with >100 pY enrichments exists in ProteomeExchange, and it is from a laboratory that developed one of the original sSH2 methods. We have rewritten the relevant Introduction sentence and added the quantitative evidence for limited adoption as the new opening Results section.

Action: Revised the Introduction sentence to: "Despite a decade of availability, sSrc has not been adopted beyond method-developing laboratories, and pY studies still analyze roughly an order of magnitude fewer samples than their global phosphoproteomic counterparts (see Results)." Supporting data for this claim is in new Figure 1 and accompanying Results section.

Reviewer #2

Point 2.1: Method novelty does not meet high criteria

> This manuscript by Chang et al. presents the development and application of R2HaPpY, a cost-effective method for phosphotyrosine (pY) peptide enrichment. This development simplifies the traditional, laborious bead preparation process, reducing hands-on time from days to hours and cutting reagent costs by up to 20-fold compared to commercial antibody-based methods, while the engineered SH2 domain based pY enrichment has already become popular. The authors rigorously demonstrate that the R2HaPpY based on Halo tag exhibit superior performance to previous methods based on NHS chemistry, are robust across a range of peptide concentrations, and remain stable for at least four months. The power of R2HaPpY is showcased in a deep, time-resolved analysis of EGF signaling in HeLa cells. This manuscript is prepared with high quality. However, given the primary focus on the incremental development of the Src superbinder approach originally reported by Bian et al. (Nat. Chem. Biol., 2016), optimization, and analytical validation of the pY enrichment approach, the method novelty does not meet high criteria.

We thank the reviewer for the recognition of rigor and performance. We address the novelty question on two fronts. First, R2HaPpY is not an optimization of the Bian *et al* (2016) approach — it is a re-engineering of reagent preparation via HaloTag fusion that eliminates protein purification and multi-step conjugation entirely. We have revised the text to make this distinction sharper.

Second, performance against commercial antibodies is established through our published work. In Chang *et al* (J. Proteome Res. 2023), NHS-sSrc beads matched or exceeded the CST pTyr-1000 antibody in head-to-head testing. Here, Halo-sSrc outperforms NHS-sSrc (Figure 2D). By transitive comparison, Halo-sSrc ≥ CST antibody at a fraction of the cost. A method that is simultaneously better-performing, 20-fold cheaper, and dramatically simpler is the advance needed to move pTyr proteomics from a specialized assay to a routine one. See also our response to Point 1.1.

Action: Added explicit transitive benchmarking (Halo-sSrc > NHS-sSrc ≥ CST pTyr-1000) to the bead chemistry Results section, anchored in our 2023 JPR head-to-head comparison.

Point 2.2: Time-resolved pY profiling lacks new biological insights

> The time-resolved pY profiling of EGF signaling has been explored repeatedly by many studies in the past and this deep profiling didn't provide and validate promising new biological insights.

EGF signaling is a well-studied system. We chose it deliberately as a benchmark, precisely because it allows us to evaluate method performance against known biology while simultaneously testing whether deeper profiling reveals previously undetected regulatory events. The value of our EGF dataset lies in three specific contributions:

(1) It is the deepest temporal pTyr dataset of EGF stimulation reported to date (1,651 sites; 878 regulated) from only ~1 mg input, providing a reference resource for the community.

(2) We identify 70 pY sites not previously reported in PhosphoSitePlus, for which we now provide manually annotated MS/MS spectra and FDR analysis confirming identification confidence.

(3) We show that temporal regulation of pY sites on the same protein frequently differs — 66% of proteins with more than one regulated pY site carry sites with distinct temporal profiles — consistent with but substantially extending Olsen *et al* (2006) at pY-specific resolution (878 pY sites vs. 53). Structural analyses of three novel sites of particular interest (RNF126 pY27, HGS pY197, WEE1 S396/Y397) are provided in Appendix (Section: Structural and Functional Implications of Novel Phosphotyrosine Sites) as starting points for dedicated functional follow-up beyond the scope of this study.

Action: Revised Discussion framing. Highlighted web resource for community data exploration (https://r2happy.gs.washington.edu/).

Point 2.3: Detailed proteomics data table

> A detailed table summarizing key proteomics data should be provided, such as protein ID, peptides, modification sites.

These data were included as Supplementary Datasets 1-3 with the original submission. We have added explicit callouts to these datasets in the Results text and in the Data Availability section. These are now titled Dataset EV2 – EV4.

Action: Added explicit citations to Datasets EV2 – EV4 in the Data Availability section.

Point 2.4: Sequence logo bias — biology vs. superbinder specificity

> The sequence logo for the novel sites reveals a consensus motif, with a preference for acidic residues at the +1 position and specific aliphatic residues at +3. A key question is whether this bias accurately reflects the biology of novel EGF sites or primarily stems from the inherent binding specificity of the sSrc superbinder.

This is an important question. The sequence motif we observe for novel sites (acidic +1, aliphatic +3) is consistent with the known binding specificity of the Src SH2 superbinder, as we reported in our 2023 JPR publication. The sequence logo in Figure 4D illustrated our hypothesis that the superbinder's enrichment preference may explain the discovery of many novel sites, not to describe a biological motif of EGF-responsive sites. In the end, we opted to omit the motif analysis as it created more confusion than clarity.

Action: Removed the motif analysis from Figure 4 (now Figure 5) to avoid sending an unclear message.

Point 2.5: Orthogonal validation of novel pY sites

> The identification of 70 completely novel pY sites is a key finding. The authors should perform orthogonal validation (e.g., using synthetic peptides or targeted MS) to confirm the existence and regulation of a subset of these high-interest novel sites, such as on GRB10, TKT, or WEE1.

We agree on the importance of bolstering confidence but note that targeted MS (e.g. PRM) primarily re-tests the acquisition and search pipeline rather than providing truly orthogonal validation. More broadly, the existence of the sites can be supported in other ways. We therefore strengthened confidence through three orthogonal lines of evidence:

(i) We provide annotated MS/MS spectra for high-interest novel sites, including GRB10, TKT, WEE1, RNF126, and others, enabling inspection of fragment ion coverage, phosphosite localization, and spectral quality (Appendix File). Predicted spectra from AlphaPeptDeep are shown as mirror-plot references where available. (ii) We present an FDR analysis for all pY sites before and after the replicate completeness filter, confirming that the novel sites meet stringent thresholds (Ascore ≥ 13, <1% FDR at PSM level, present in ≥3 of 6 replicates of a condition). (iii) Many novel sites exhibit clear, reproducible EGF-responsive temporal regulation across biological replicates — a pattern that false-positive identifications would not produce. Together, spectral evidence, statistical rigor, and biological reproducibility provide robust confidence in these identifications.

Action: Added annotated MS/MS spectra for a subset of novel pY sites as a new Supplementary File 3. Added detailed FDR analysis. Discussed spectral quality and reproducibility as evidence for identification confidence.

Point 2.6: Variation in pTyr site intensities (e.g., PRPF31 Y205)

> The intensity of pTyr sites (e.g., PRPF31 Y205 in Figure 5C) exhibits substantial variation in the EGF stimulation experiments. What potential factors might contribute to this observed heterogeneity?

Phosphosite-level variation is a well-documented challenge in phosphoproteomics and is generally larger than protein-level variation because phosphosite quantification typically relies on a single peptide measurement. Three factors contribute: (i) biological variation across replicates, which is inherently higher for phosphorylation than protein abundance (Faca *et al*, 2020); (ii) stochastic precursor sampling in DDA, particularly for lower-abundance precursors (Hogrebe *et al*, 2018; Bekker-Jensen *et al*, 2020); and (iii) quantification noise at low intensity.

Prompted by this question, we performed a focused analysis of PRPF31 pY205 in the context of the full dataset. Precursor rollup has intensity-dependent effects: aggregating across precursors reduces CVs for abundant sites (a ~4% improvement in the top intensity tertile) while slightly increasing variation for low abundance sites (~4.5% in the bottom tertile) where species detection is more stochastic. PRPF31 pY205 is a low abundance site with two stochastically detected precursor species; aggregating them improves data completeness at the cost of ~4% additional variance, but the EGF response profile of the site is unchanged regardless of which precursor is used. This intensity-dependent pattern is the principal reason we used six biological replicates rather than the standard three. The additional replicates add statistical power for these low abundance sites. A figure summarizing this analysis is provided below for reviewers (Review Figure 4) and the concept has been incorporated into the main text.

Action: Added a brief note in the Results on phosphosite measurement variability and on the rationale for six biological replicates. The full precursor-rollup analysis and the PRPF31 pY205 case study are included in this response for reviewer reference.

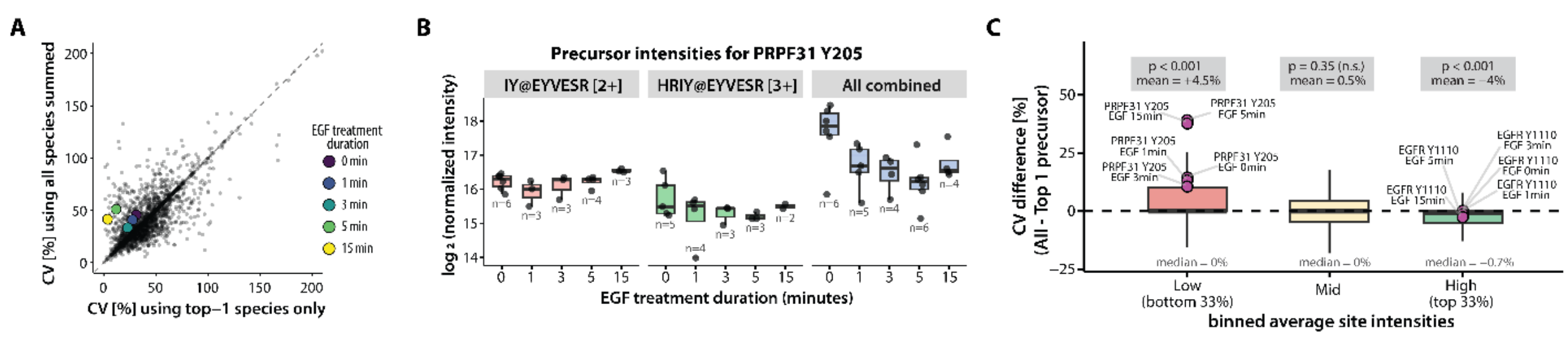

Review Figure 4. PRPF31 pY205 is a low intensity site where aggregation of precursors increases variation

A. Scatter plot compares coefficients of variation (CV) when aggregating all precursors (y-axis) to CV based on the highest intensity precursor. Highlighted points represent PRPF31 pY205 measurements for each EGF stimulation condition. Aggregation of both precursors increases CV for PRPF31 pY205.

B. Boxplots with points show measured intensities of both precursors measured for PRPF31 pY205 across EGF stimulation conditions. The number of measured replicates is indicated below the lowest intensity point. Intensities are displayed across facets for each precursor separately and combined, as originally reported.

C. Boxplots indicate the difference in CV if all precursors are aggregated versus using only the most intense precursor (y-axis). Sites were binned into tertiles to separate effects of precursor aggregation across low, moderate, and highest intensity sites (x-axis). Points indicate CV differences from precursor aggregation for PRPF31 pY205 (site in question) and EGFR pY1110 (abundant reference site). Mean CV change as well as Wilcoxon ranked sum test significance of the CV difference from precursor aggregation is listed inside grey boxes at top of plot. Median CV differences are listed along the x-axis at bottom of plot.

Takeaway: While median CV is largely unchanged, precursor selection strategy alters variation in an intensity dependent fashion. Low intensity sites suffer variation increase of 4.5% on average while high intensity sites benefit from 4% decrease in variation upon precursor aggregation. Despite variation

increase for low intensity sites due to stochastic detection of different precursor species, aggregation was chosen as replicate completeness improves.

Point 2.7: INPPL1 S1003 (pSer) in Figure 6E

> INPPL1 S1003 (pSer) was included in Figure 6E. Given that this study primarily focuses on pTyr proteomics, what evidence supports the reliability of this phosphoserine site identification?

INPPL1 pS1003 was identified from our parallel global phosphoproteome data (Fe-IMAC enrichment) and is shown in Figure 6 (now Figure 7 in revised main text) to illustrate how pY-specific and global phosphoproteomic enrichments on the same sample together resolve regulatory PTM layers that neither approach captures alone. Its identification and quantification follow the same stringent criteria applied to all sites in the global phosphoproteome dataset (Ascore ≥ 13, <1% FDR at PSM level, ≥50% replicate completeness in a condition). We have clarified the figure legend accordingly.

Action: Clarified in the Figure 6 (now Figure 7) legend and main text that INPPL1 pS1003 was identified from the global phosphoproteome (Fe-IMAC) dataset.

Point 2.8: EGF concentration (160 ng/mL)

> The study employs EGF at 160 ng/mL, a notably high concentration compared to typical physiological or experimental ranges. What rationale supports this dosage selection?

Literature shows 50-200 ng/mL range for acute signal propagation. We chose a high concentration of EGF to investigate associated receptor signaling and endocytic sorting.

Action: We have added a sentence in the Methods clarifying this rationale and noting that users applying R2HaPpY to study dose-dependent biology should titrate concentration to their specific experimental question.

Reviewer #3

Point 3.1: Unclear significance of advance over 2023 JPR publication

Overall I found this manuscript to be interesting; however, it is unclear how significant of an advance it offers over the prior JPR publication.

We appreciate this concern and have revised the manuscript to more explicitly delineate the advances over our 2023 JPR publication. The key differences are: (1) Bead preparation is fundamentally redesigned—the JPR method required His-tag affinity purification, buffer exchange, and NHS conjugation over 3 days; R2HaPpY uses a single-step HaloTag conjugation from crude lysate in 1-2 hours. (2) The Halo-sSrc beads outperform NHS-sSrc beads in pY site yield and signal intensity (Figure 2D), likely due to site-specific oriented conjugation that avoids blocking the pY-binding interface. (3) Cost per 96 samples is reduced from $2,554 (NHS-sSrc) to $551 (Halo-sSrc). (4) The method is validated across peptide concentrations and bead storage conditions, demonstrating robustness for large-scale studies. These are not parameter optimizations within the same framework; they represent a re-engineering of the reagent and workflow.

Action: Revised introduction, results and discussion to articulate gaps facing the NHS-based bead conjugation and more broadly phosphotyrosine proteomics workflows that are fulfilled by the HaloTag-based approach.

Point 3.2: Improvement primarily aimed at reducing cost

> Obviously, methods that allow for faster generation of the reagent are of use and worthy of publication. But this improvement is primarily aimed at reducing cost, which doesn't necessarily drive impact. Some improvement in depth over the older Src method is shown in Figure 1D and this is strong but the comparison is not done in a rigorous or comprehensive enough way.

We respectfully disagree that our primary aim and contribution is cost reduction. We improved ease of use, robustness, scalability, and cost. In proteomics and high-throughput biology broadly, cost, ease of use, and scalability are frequently decisive for whether a technique is adopted community-wide or remains confined to specialist laboratories. The trajectory of DNA sequencing illustrates this principle clearly: successive reductions in per-base-cost – from Sanger to next-generation platforms – did not merely make existing experiments cheaper but fundamentally expanded the scope of questions that could be addressed (Kris A. Wetterstrand, 2019). Population-scale genomics (UK Biobank Whole-Genome Sequencing Consortium, 2025), clinical whole-exome diagnostics (Yang *et al*, 2013), and single cell transcriptomics (Single-cell analysis hits its stride, 2015; Macosko *et al*, 2015; Zheng *et al*, 2017) were all enabled, not by new sequencing chemistry alone, but in large part by cost reductions that made previously inconceivable study designs practical. pTyr proteomics has been technically feasible for over a decade yet remains underrepresented in large-scale studies such as CPTAC and the Cancer Cell Line Encyclopedia precisely because of cost and throughput barriers (new Figure 1). A 20-fold cost and time reduction, combined with improved performance and robustness, impacts method adoption and directly enables study designs that were previously impractical and inaccessible to most labs, e.g. profiling pY signaling across hundreds of patient samples, drug conditions, or time points.

Beyond furthering biological knowledge, the new data generation will enable accurate predictions from phosphotyrosine data. With the rise of deep learning methods, data availability is becoming increasingly important.

Currently, the majority of the human pY landscape derives from internal studies at Cell Signaling Technologies Inc., whose underlying data are not publicly available. Of the known pY sites validated by at least three independent studies, only a small fraction (24%; 3,167 sites) is publicly available from external sources (new Figure 1A). This small dataset trains current phosphosite function, kinase activity,

and mutation-impact models, and contributes to their weaker performance on pY relative to pS/pT (Müller-Dott *et al*, 2025; Ochoa *et al*, 2020; Jiang *et al*, 2025). Scaling pY enrichment to the sample numbers that now define global phosphoproteomics is a prerequisite for closing that gap.

Action: Added new Figure 1 and opening Results section quantifying the community adoption gap; revised the Introduction accordingly.

Points 3.3, 3.4, 3.5: Comparison with commercial approaches

Some improvement in depth over the older Src method is shown in Figure 1D and this is strong but the comparison is not done in a rigorous or comprehensive enough way. A good amount of the manuscript is then dedicated towards the application to EGFR analysis. In my view the work would need to be considerably expanded to flesh out some of the comparisons with commercial approaches that are mentioned but not tested. I think this is a major limitation. Even with these comparisons a concern that the improved method is more of an incremental advance over the 2023 JPR paper. [...] The comparisons of Figure 1 should be expanded to include commercial pY antibodies like CST PTMscan and Axobind Superbinder.

We agree that commercial benchmarking is important and note that for CST antibody, this comparison has already been established in our published work. In Chang *et al* (J. Proteome Res. 2023, 22, 1868-1880) we performed a direct head-to-head comparison of NHS-sSrc beads against the CST pTyr-1000 antibody on the same sample and showed that NHS-sSrc matched or exceeded the antibody in pY site yield. In the present manuscript, Halo-sSrc outperforms NHS-sSrc (Figure 1D; Figure EV1 and Appendix Figure S1). By transitive comparison, Halo-sSrc ≥ CST pTyr-1000, at a fraction of the cost.

Regarding Axobind Superbinder: this reagent became commercially available during the review period of this manuscript and has not yet been independently benchmarked in a peer-reviewed study. We do not consider it an appropriate comparator at this time, though its commercial release supports the broader point that the fusion-protein approach is gaining adoption.

Action: Added the explicit transitive benchmarking statement (Halo-sSrc > NHS-sSrc ≥ CST pTyr-1000) to the HaloLink bead chemistry Results section, referencing Chang *et al* 2023 JPR.

Point 3.6: Performance as a function of load compared directly

> The performance as a function of load should also be compared directly.

We would like to point out that the suggested analysis of performance as a function of load was already shown for the Halo-sSrc and NHS-sSrc across peptide inputs from 0.125 to 1 mg (Figure EV1), demonstrating robust performance across the input range relevant to typical experimental designs.

Point 3.7: MS instrument and DIA considerations

> The MS details are not really presented and I wonder how these would perform using the current generation of fast scanning TOF systems like Tims TOF or Astral with DIA?

We have expanded the MS methods section to state explicitly that all data were acquired in data-dependent acquisition (DDA) mode on the Thermo Exploris 480 Orbitrap. Beyond this, the MS methods contain sufficient detail for independent reproduction of the acquisition. We expect that R2HaPpY coupled with timsTOF or Astral DIA would likely yield further gains in depth and quantitative precision, consistent with recent reports (Kumar *et al*, 2025). Importantly, the enrichment strategy is instrument-agnostic — bead preparation and enrichment are upstream of the MS measurement — so R2HaPpY can be paired with whatever MS platform a laboratory has access to.

Action: Expanded the MS methods section to specify DDA acquisition on the Thermo Orbitrap Exploris 480.

Point 3.8: Use of pervanadate

> The hela work used pervandate which seems unnecessary and unhelpful in comparing to other works. Would be preferred to not use this and artificially inflate amount of pY.

Pervanadate was used in some, but not all, method-development and reagent-characterization experiments (Figures 2, 3, EV1, and EV3), where maximizing pY load is desirable for evaluating bead capacity, enrichment efficiency, and reproducibility. The EGF signaling experiments (Figures 3-6), which are the biological application and the majority of the manuscript, were performed without pervanadate. We also include untreated HeLa enrichments (Appendix Figure S3) specifically to confirm performance in a low-pY regime more representative of physiological samples. The dual-sample strategy, pervanadate for capacity maximization and no-pervanadate for physiological benchmarking, is standard in the tyrosine phosphoproteomics field (Rush *et al*, 2005; Sharma *et al*, 2014; Bian *et al*, 11/2016; Dong *et al*, 2017; Tong *et al*, 2017; Yao *et al*, 2018, 2019; Chua *et al*, 04/2020; Kong *et al*, 2022; Martyn *et al*, 2022; Chang *et al*, 2023). The revised text makes this split explicit in the relevant Results section.

Action: Revised the robustness-across-concentrations Results section to state clearly that both pervanadate-treated and untreated cells were used for reagent characterization and that untreated HeLa and EGF-stimulated samples establish biological performance (Appendix Figure S3, Figures 4-7).

Point 3.9: Section headings

> The section headings seem to be written by a marketing person and don't really give the impression of an unbiased experiment. E.g., "Halo-sSrc beads are stable for > 4 months", "Enrichment with Halo-sSrc is robust to various peptide concentrations", "HaloLink bead chemistry simplifies….", "R2HaPpy deeply measures pY signaling…"

We respectfully disagree with this comment. The headings were not written by a marketing person. We are a fully academic lab with no commercial interest and choose titles reflective of the intended message. The headings describe the specific experimental findings presented in each section, and we have retained them as concise, accurate descriptors of what the reader will find.

References:

Bekker-Jensen DB, Bernhardt OM, Hogrebe A, Martinez-Val A, Verbeke L, Gandhi T, Kelstrup CD, Reiter L & Olsen JV (2020) Rapid and site-specific deep phosphoproteome profiling by data-independent acquisition without the need for spectral libraries. *Nat Commun* 11: 787

Bian Y, Li L, Dong M, Liu X, Kaneko T, Cheng K, Liu H, Voss C, Cao X, Wang Y, *et al* (11/2016) Ultra-deep tyrosine phosphoproteomics enabled by a phosphotyrosine superbinder. *Nat Chem Biol* 12: 959–966

Bittremieux W (2020) Spectrum_utils: A Python package for mass spectrometry data processing and visualization. *Anal Chem* 92: 659–661

Bittremieux W, Levitsky L, Pilz M, Sachsenberg T, Huber F, Wang M & Dorrestein PC (2023) Unified and standardized mass spectrometry data processing in Python using spectrum_utils. *J Proteome Res* 22: 625–631

Chang A, Leutert M, Rodriguez-Mias RA & Villén J (2023) Automated Enrichment of Phosphotyrosine Peptides for High-Throughput Proteomics. *J Proteome Res* 22: 1868–1880

Chua XY, Mensah T, Aballo T, Mackintosh SG, Edmondson RD & Salomon AR (04/2020) Tandem Mass Tag Approach Utilizing Pervanadate BOOST Channels Delivers Deeper Quantitative Characterization of the Tyrosine Phosphoproteome. *Mol Cell Proteomics* 19: 730–743

Dong M, Bian Y, Wang Y, Dong J, Yao Y, Deng Z, Qin H, Zou H & Ye M (2017) Sensitive, Robust, and Cost-Effective Approach for Tyrosine Phosphoproteome Analysis. *Anal Chem* 89: 9307–9314

Faca VM, Sanford EJ, Tieu J, Comstock W, Gupta S, Marshall S, Yu H & Smolka MB (2020) Maximized quantitative phosphoproteomics allows high confidence dissection of the DNA damage signaling network. *Sci Rep* 10: 18056

Hogrebe A, von Stechow L, Bekker-Jensen DB, Weinert BT, Kelstrup CD & Olsen JV (2018) Benchmarking common quantification strategies for large-scale phosphoproteomics. *Nat Commun* 9: 1045

Humphrey ES, Su S-P, Nagrial AM, Hochgräfe F, Pajic M, Lehrbach GM, Parton RG, Yap AS, Horvath LG, Chang DK, *et al* (2016) Resolution of novel pancreatic ductal adenocarcinoma subtypes by global phosphotyrosine profiling. *Mol Cell Proteomics* 15: 2671–2685

Jiang W, Jaehnig EJ, Liao Y, Shi Z, Yaron-Barir TM, Johnson JL, Cantley LC & Zhang B (2025) Deciphering the dark cancer phosphoproteome using machine-learned co-regulation of phosphosites. *Nat Commun* 16: 2766

Kong Q, Weng Y, Zheng Z, Chen W, Li P, Cai Z & Tian R (2022) Integrated and High-Throughput Approach for Sensitive Analysis of Tyrosine Phosphoproteome. *Anal Chem* 94: 13728–13736

Kris A. Wetterstrand MS (2019) DNA Sequencing Costs: Data. *Genome.gov*

Kumar M, Possemato AP, Zee BM, Subramanian S, Ren JM, Nelson AJ, Zhang B, Landry S, Silva JC, Larsen B, *et al* (2025) Expanding the global map of protein post translational modifications with immunoaffinity enrichment and nDIA analysis on the Orbitrap Astral mass spectrometer. *bioRxiv*: 2025.07.03.662636

Lautenbacher L, Yang KL, Kockmann T, Panse C, Gabriel W, Bold D, Kahl E, Chambers M, MacLean BX, Li K, *et al* (2025) Koina: Democratizing machine learning for proteomics research. *Nat Commun* 16: 1–13

Macosko EZ, Basu A, Satija R, Nemesh J, Shekhar K, Goldman M, Tirosh I, Bialas AR, Kamitaki N, Martersteck EM, *et al* (2015) Highly parallel genome-wide expression profiling of individual cells using nanoliter droplets. *Cell* 161: 1202–1214

Martyn GD, Veggiani G, Kusebauch U, Morrone SR, Yates BP, Singer AU, Tong J, Manczyk N, Gish G, Sun Z, *et al* (2022) Engineered SH2 Domains for Targeted Phosphoproteomics. *ACS Chem Biol*

Mirhadi S, Tam S, Li Q, Moghal N, Pham N-A, Tong J, Golbourn BJ, Krieger JR, Taylor P, Li M, *et al* (2022) Integrative analysis of non-small cell lung cancer patient-derived xenografts identifies distinct proteotypes associated with patient outcomes. *Nat Commun* 13: 1811

Müller-Dott S, Jaehnig EJ, Munchic KP, Jiang W, Yaron-Barir TM, Savage SR, Garrido-Rodriguez M, Johnson JL, Lussana A, Petsalaki E, *et al* (2025) Comprehensive evaluation of phosphoproteomic-based kinase activity inference. *Nat Commun* 16: 4771

Ochoa D, Jarnuczak AF, Viéitez C, Gehre M, Soucheray M, Mateus A, Kleefeldt AA, Hill A, Garcia-Alonso L, Stein F, *et al* (2020) The functional landscape of the human phosphoproteome. *Nat Biotechnol* 38: 365–373

Qin Z, Xiang C, Zhong F, Liu Y, Dong Q, Li K, Shi W, Ding C, Qin L & He F (2019) Transketolase (TKT) activity and nuclear localization promote hepatocellular carcinoma in a metabolic and a non-metabolic manner. *J Exp Clin Cancer Res* 38: 154

Rush J, Moritz A, Lee KA, Guo A, Goss VL, Spek EJ, Zhang H, Zha X-M, Polakiewicz RD & Comb MJ (2005) Immunoaffinity profiling of tyrosine phosphorylation in cancer cells. *Nat Biotechnol* 23: 94–101

Sharma K, D'Souza RCJ, Tyanova S, Schaab C, Wiśniewski JR, Cox J & Mann M (2014) Ultradeep human phosphoproteome reveals a distinct regulatory nature of Tyr and Ser/Thr-based signaling. *Cell Rep* 8: 1583–1594

Single-cell analysis hits its stride (2015) *https://www.broadinstitute.org/*

Tong J, Cao B, Martyn GD, Krieger JR, Taylor P, Yates B, Sidhu SS, Li SSC, Mao X & Moran MF (2017) Protein-phosphotyrosine proteome profiling by superbinder-SH2 domain affinity purification mass spectrometry, sSH2-AP-MS. *Proteomics* 17

UK Biobank Whole-Genome Sequencing Consortium (2025) Whole-genome sequencing of 490,640 UK Biobank participants. *Nature* 645: 692–701

Yang Y, Muzny DM, Reid JG, Bainbridge MN, Willis A, Ward PA, Braxton A, Beuten J, Xia F, Niu Z, *et al* (2013) Clinical whole-exome sequencing for the diagnosis of mendelian disorders. *N Engl J Med* 369: 1502–1511

Yao Y, Bian Y, Dong M, Wang Y, Lv J, Chen L, Wang H, Mao J, Dong J & Ye M (2018) SH2 Superbinder Modified Monolithic Capillary Column for the Sensitive Analysis of Protein Tyrosine Phosphorylation. *J Proteome Res* 17: 243–251

Yao Y, Wang Y, Wang S, Liu X, Liu Z, Li Y, Fang Z, Mao J, Zheng Y & Ye M (2019) One-Step SH2 Superbinder-Based Approach for Sensitive Analysis of Tyrosine Phosphoproteome. *J Proteome Res* 18: 1870–1879

Zeng W-F, Zhou X-X, Willems S, Ammar C, Wahle M, Bludau I, Voytik E, Strauss MT & Mann M (2022) AlphaPeptDeep: a modular deep learning framework to predict peptide properties for proteomics. *Nat Commun* 13: 7238

Zheng GXY, Terry JM, Belgrader P, Ryvkin P, Bent ZW, Wilson R, Ziraldo SB, Wheeler TD, McDermott GP, Zhu J, *et al* (2017) Massively parallel digital transcriptional profiling of single cells. *Nat Commun* 8: 14049

1st Editorial Decision **22nd May 2026**

Dr. Judit Villen
University of Washington
Genome Sciences
3720 15th Ave NE
Foege Bldg, S133
Seattle, WA 98195

22nd May 2026

Re: EMBOJ-2026-124294-T
Phosphotyrosine Proteomics Using a Scalable SH2 Superbinder Enrichment Strategy

Dear Dr. Villen,

Thank you for transferring your previously reviewed and revised manuscript to The EMBO Journal. Following my own reading of the study, reports, and responses, I decided to discuss it with a trusted arbitrator of our journal, in order to better gauge the potential resource value of your method compared to available alternatives. Having now received supportive feedback (see below), we shall be happy to pursue the study further for EMBO Journal publication, as soon as the following editorial points are incorporated into a final version of the manuscript (please also consider our more detailed online Guide to Authors):

GENERAL:

- Please download and complete our author checklist (link provided below).

- Please upload the manuscript text (including figure legends) as an editable text file, and all main and expanded view figures without legends as individual image files with sufficient resolution/quality for production.

- Please add a direct URL in the Data Availability section for each database in which data or code generated in the course of the study has been deposited. Please ensure that the proteomics data become publicly accessible latest by the time of publication.

- For the Appendix file:
Please preface it with a title page stating: 'Appendix for [manuscript title]'
The Table of Contents should include page numbers, and list individual items rather than just sections (e.g. Appendix Figure S1/2 rather than just Appendix Figures)
Please rename the section 'Appendix Note' into 'Appendix Text S1, and the included references list as 'Appendix References'. Section 4 on Summary of Total Proteome Changes should become 'Appendix Text S2', section 5 on Detailed Cost should become 'Appendix Text S3', and all of them be referenced as such in the main text at least once.
The Expression Construct plasmid maps might most aptly be renamed as additional Appendix Figures S16 & S17; while the FASTA-formatted sequence may be more useful if provided in a text file, e.g. in DOCX format as Dataset EV5?
Finally, I would suggest cleaning up the ordering: first Appendix Text S1/2/3, then Appendix References, followed by Appendix Figures S1-S17

- Please provide suggestions for a short 'blurb' text prefacing and summing up the conceptual aspect of the study in two sentences (max. 250 characters), followed by 3-5 one-sentence 'bullet points' with brief factual statements of key results of the paper; they will form the basis of an editor-written 'Synopsis' accompanying the online version of the article. Please also upload a synopsis image, which can be used as a "visual title" for the synopsis section of your paper. The image should be ideally in JPG format, and please make sure that it remains in the modest dimensions of (exactly) 550 pixels wide and 300 pixels high.

- Regarding the title, I wonder if it should not also highlight the extensive new EGF-responsive phosphoproteome dataset a little? Some initial ideas below, happy to discuss better options:
'Phosphotyrosine Proteomics Using a Scalable SH2 Superbinder Enrichment Strategy facilitates deep temporal profiling of EGF responses'
'A Scalable SH2 Superbinder Enrichment Strategy for Phosphotyrosine Proteomics provides insights into temporal EGF responses'
'A Scalable SH2 Superbinder Enrichment Strategy facilitates deep Phosphotyrosine Proteomic profiling of EGF responses'

TEXT:

- Please adjust the order as well as the headers of the different manuscript sections: Title page with complete author information, Abstract, Introduction, Results, Discussion (possibly combined with Results), Methods, Data Availability,

Acknowledgements, Disclosure and Competing Interests Statement, References, Main Figure Legends, Tables, Expanded View Figure Legends.

- As we are switching from a free-text author contribution statement towards a more formal statement based on Contributor Role Taxonomy (CRediT) terms, please remove the present Author Contribution section and instead specify each author's contribution(s) directly in the Author Information page of our submission system during upload of the final manuscript. See https://casrai.org/credit/ for more information.

- Please double-check the format, correctness and completeness of the reference list and in-text citations. While the overall format appears to adhere to EMBO Journal guidelines, several references appear to lack complete volume and/or page numbers; and others cite not just publication year but also months.
Furthermore, for preprint citations: Please change in-text referencing according to the format "preprint: Miller et al, 2025"; and in the reference list, continue after the manuscript title with the name of the preprint platform plus DOI + preprint label - e.g. "bioRxiv doi: 1234/002.dfj123 [PREPRINT]"
Finally, we encourage formal citation of datasets obtained from public resources in the form of 'data citations' (see https://link.springer.com/journal/44318/submission-guidelines#cms-Reference-guidelines for detail); similar to preprint citations, they should have the prefix "Data ref:" in the text, and that tag [DATASET} inside the reference list.

- Please refer to our Guide to Authors (https://link.springer.com/journal/44318/submission-guidelines#cms-Structured-methods) regarding the 'Structured Methods' required for Method Articles in The EMBO Journal. Make sure to upload a Reagents & Tools table based on the template downloadable from our site (https://media.springernature.com/original/springer-cms/rest/v1/content/27825802/data/v1). For methods articles, we encourage key descriptions in a step-by-step protocol format (but you may also place an in-text reference to protocols.io with the respective DOI).

- Please make sure to reference each figure and each panel at least once from the main text (if referring to a whole multi-panel figure at once, simply state e.g. Figure 1A-D instead of Figure 1).

DATA:

- For each Expanded View dataset, please include a separate 'Legends' tab in the XLSX workbook containing title and any legend information for the respective dataset. On the other hand, the table 'Summary of additional material' should be removed from the main manuscript text.

- During routine pre-acceptance checks, our data editors have raised the following queries regarding figures, data, and legends; I would appreciate if you briefly answered to them in the cover letter of your final submission, and made the requested text modifications with changes/additions highlighted via the "Track changes" option, to facilitate our final checking":
1. Please define the exact p values in the legends of 5B.
2. Please indicate the statistical test used for data analysis in the legends of figure 6D.
3. Please define the box plots in terms of minima, maxima, centre, bounds of box and whiskers, and percentile in the legends of figures 3B; 5B; 6C; 7B,C,D.
4. Please provide information as to the nature of "n" in the legends 3B.
5. Please define the error bars in the legends of 2D.
6. Please define the measure of center for the error bars in the legends of figures 4A,B; EV-1A; EV-3A; EV-4A.

- Finally, you shall also receive a separate message from our Source Data curation team, with instructions on how to prepare and upload relevant image and numerical raw data.

I am returning the manuscript to you now for the final revisions, with the hyperlink below allowing you to upload all files once you are ready. Should you need additional guidance/feedback regarding this final adjustments, please do not hesitate to contact us directly. Thank you again for the opportunity to consider this work for The EMBO Journal, and I look forward to receiving your final version!

Yours sincerely,

Hartmut Vodermaier

Hartmut Vodermaier, PhD
Senior Editor, The EMBO Journal
h.vodermaier@embojournal.org

*** PLEASE NOTE: All revised manuscript are subject to initial checks for completeness and adherence to our formatting

guidelines. Revisions may be returned to the authors and delayed in their editorial re-evaluation if they fail to comply to the following requirements. As a first step please read our guidelines for revised submissions:
https://link.springer.com/journal/44318/submission-guidelines#cms-Revised-submissions

1) Every manuscript requires a Data Availability section (even if only stating that no deposited datasets are included). Primary datasets or computer code produced in the current study have to be deposited in appropriate public repositories prior to resubmission, and reviewer access details provided in case that public access is not yet allowed.

2) Each figure legend must specify
- size of the scale bars that are mandatory for all micrograph panels
- the statistical test used to generate error bars and P-values
- the type error bars (e.g., S.E.M., S.D.)
- the number (n) and nature (biological or technical replicate) of independent experiments underlying each data point
- Figures may not include error bars for experiments with n<3; scatter plots showing individual data points should be used instead.

3) Revised manuscript text (including main tables, and figure legends for main and EV figures) has to be submitted as editable text file (e.g., .docx format). We encourage highlighting of changes (e.g., via text color) for the referees' reference.

4) Each main and each Expanded View (EV) figure should be uploaded as individual production-quality files (preferably in .eps, .tif, .jpg formats). For suggestions on figure preparation/layout, please refer to our Figure Preparation Guidelines:
https://media.springernature.com/original/springer-cms/rest/v1/content/27825798/data/v1

5) Point-by-point response letters should include the original referee comments in full together with your detailed responses to them (and to specific editor requests if applicable), and also be uploaded as editable (e.g., .docx) text files.

6) Please complete our Author Checklist, and make sure that information entered into the checklist is also reflected in the manuscript; the checklist will be available to readers as part of the Review Process File.

7) All authors listed as (co-)corresponding need to deposit, in their respective author profiles in our submission system, a unique ORCiD identifier linked to their name. Please see our Guide to Authors for detailed instructions.

8) Please note that supplementary information at EMBO Press has been superseded by the 'Expanded View' for inclusion of additional figures, tables, movies or datasets; with up to five EV Figures being typeset and directly accessible in the HTML version of the article.

9) To facilitate reproducibilty and cross-laboratory adoption of methodologies, please structure the Materials & Methods section as outlined in our guide to authors, including a completed Reagents and Tools Table.

10) Digital image enhancement is acceptable practice, as long as it accurately represents the original data and conforms to community standards. If a figure has been subjected to significant electronic manipulation, this must be clearly noted in the figure legend and/or the 'Materials and Methods' section. The editors reserve the right to request original versions of figures and the original images that were used to assemble the figure. Finally, we generally encourage uploading of numerical as well as gel/blot image source data.

At EMBO Press we ask authors to provide source data for the main manuscript figures. You will receive a separate email with instructions for providing source data with your revised manuscript, including how to upload and organize the files.

In the interest of ensuring the conceptual advance provided by the work, we recommend submitting a revision within 3 months (20th Aug 2026). Please discuss the revision progress ahead of this time with the editor if you require more time to complete the revisions. Use the link below to submit your revision:

Link Not Available

*** Arbitrating Advisor's Comments ***

I looked through the ms and responses. I do think the work is a strong candidate for EMBOJ. Tyrosine phosphorylation is a very important yet extremely challenging area of signaling and PTM proteomics. To be able to do it very robustly at scale with a very fast turnaround (no competing literature has achieved this so far) is a major milestone and warrants it as an important resource. The referee comments are properly addressed in my opinion, no further comments there.

Editor and Arbitrating Referee Response from EMBO Journal

From: <h.vodermaier@embojournal.org>
Date: Fri, May 22, 2026, 00:45
Subject: Manuscript EMBOJ-2026-124294-T - Decision
To: <jvillen@uw.edu>

Dr. Judit Villen
University of Washington
Genome Sciences
3720 15th Ave NE
Foege Bldg, S133
Seattle, WA 98195

22nd May 2026

Re: EMBOJ-2026-124294-T
Phosphotyrosine Proteomics Using a Scalable SH2 Superbinder Enrichment Strategy

Dear Dr. Villen,

Thank you for transferring your previously reviewed and revised manuscript to The EMBO Journal. Following my own reading of the study, reports, and responses, I decided to discuss it with a trusted arbitrator of our journal, in order to better gauge the potential resource value of your method compared to available alternatives. Having now received supportive feedback (see below), we shall be happy to pursue the study further for EMBO Journal publication, as soon as the following editorial points are incorporated into a final version of the manuscript (please also consider our more detailed online Guide to Authors):

*** Arbitrating Advisor's Comments ***

I looked through the ms and responses. I do think the work is a strong candidate for EMBOJ. Tyrosine phosphorylation is a very important yet extremely challenging area of signaling and PTM proteomics. To be able to do it very robustly at scale with a very fast turnaround (no competing literature has achieved this so far) is a major milestone and warrants it as an important resource.
The referee comments are properly addressed in my opinion, no further comments there.

General Changes Requested

- Please download and complete our author checklist (link provided below).
Action: Completed and uploaded to submission portal.

- Please upload the manuscript text (including figure legends) as an editable text file, and all main and expanded view figures without legends as individual image files with sufficient resolution/quality for production.
Action: Completed and uploaded to submission portal as 'Main Text – clean'.

- Please add a direct URL in the Data Availability section for each database in which data or code generated in the course of the study has been deposited. Please ensure that the proteomics data become publicly accessible latest by the time of publication.
Action: The data availability section contains links to data and code. Proteomics data will be made accessible by the time of publication.

- For the Appendix file:

- Please preface it with a title page stating: 'Appendix for [manuscript title]'
    - **Action:** Appendix title page has been added.

- The Table of Contents should include page numbers, and list individual items rather than just sections (e.g. Appendix Figure S1/2 rather than just Appendix Figures)
    - **Action:** Table of contents has been updated to include page numbers and figure numbers.

- Please rename the section 'Appendix Note' into 'Appendix Text S1, and the included references list as 'Appendix References'. Section 4 on Summary of Total Proteome Changes should become 'Appendix Text S2', section 5 on Detailed Cost should become 'Appendix Text S3', and all of them be referenced as such in the main text at least once.
  Action: Appendix notes have been renamed to Appendix Text S1 – S3.

- The Expression Construct plasmid maps might most aptly be renamed as additional Appendix Figures S16 & S17; while the FASTA-formatted sequence may be more useful if provided in a text file, e.g. in DOCX format as Dataset EV5?
  Action: Expression construct plasmid maps have been renamed as Appendix Figures S16 and S17. The FASTA-formatted sequences have been provided in a new Dataset EV5 DOCX file.

- Finally, I would suggest cleaning up the ordering: first Appendix Text S1/2/3, then Appendix References, followed by Appendix Figures S1-S17
  Action: Appendix content has been reordered as suggested.

- Please provide suggestions for a short 'blurb' text prefacing and summing up the conceptual aspect of the study in two sentences (max. 250 characters), followed by 3-5 one-sentence

'bullet points' with brief factual statements of key results of the paper; they will form the basis of an editor-written 'Synopsis' accompanying the online version of the article.

Two Sentence Blurb:

R2HaPpY enables large-scale tyrosine phosphoproteomics studies by simplifying bead preparation and automating phosphopeptide enrichment. Applied to EGF-stimulated cells, it yields the deepest temporal phosphotyrosine dataset of EGF signaling reported to date.

Synopsis Bullet Points:

- R2HaPpY simplifies the preparation of phosphotyrosine superbinder capture reagent, expediting this process from multiple days to one hour.
- The sensitivity of the R2HaPpY method is comparable to phosphotyrosine enrichment with antibodies and the reagent cost is ~20 times lower.
- Phosphotyrosine peptide enrichment with R2HaPpY is highly reproducible, exceeds 99% efficiency across a wide range of sample inputs, and runs in an automated format that scales to hundreds of samples.
- Applied to EGF-stimulated cells, R2HaPpY quantified 1,651 phosphotyrosine sites—878 of them dynamically regulated—the largest temporal map of phosphotyrosine signaling for this pathway to date and resolves how individual proteins are regulated differently at distinct sites.
- The dataset includes 70 previously unreported phosphotyrosine sites, such as pY27 in the E3 ligase RNF126, pY21 in SUMO1, pY197 in HGS, and pY4 in transketolase (TKT).

Please also upload a synopsis image, which can be used as a "visual title" for the synopsis section of your paper. The image should be ideally in JPG format, and please make sure that it remains in the modest dimensions of (exactly) 550 pixels wide and 300 pixels high.

Action: Uploaded a synopsis image.

Regarding the title, I wonder if it should not also highlight the extensive new EGF-responsive phosphoproteome dataset a little? Some initial ideas below, happy to discuss better options:

- 'Phosphotyrosine Proteomics Using a Scalable SH2 Superbinder Enrichment Strategy facilitates deep temporal profiling of EGF responses'
- 'A Scalable SH2 Superbinder Enrichment Strategy for Phosphotyrosine Proteomics provides insights into temporal EGF responses'
- 'A Scalable SH2 Superbinder Enrichment Strategy facilitates deep Phosphotyrosine Proteomic profiling of EGF responses'

We appreciate the suggestion to include the extensive EGF-responsive phosphoproteome dataset in the title.

Action: Changed title to 'Scalable Phosphotyrosine Enrichment with SH2 Superbinder Enables Deep Profiling of EGF Responses'

Text-specific Changes Requested:

Please adjust the order as well as the headers of the different manuscript sections: Title page with complete author information, Abstract, Introduction, Results, Discussion (possibly combined with Results), Methods, Data Availability, Acknowledgements, Disclosure and Competing Interests Statement, References, Main Figure Legends, Tables, Expanded View Figure Legends.
Action: Sections have been reordered, and headers have been updated.

As we are switching from a free-text author contribution statement towards a more formal statement based on Contributor Role Taxonomy (CRediT) terms, please remove the present Author Contribution section and instead specify each author's contribution(s) directly in the Author Information page of our submission system during upload of the final manuscript. See https://casrai.org/credit/ for more information.
Action: We have removed the Author Contribution section and instead have indicated contributions directly in the submission system.

Please double-check the format, correctness and completeness of the reference list and in-text citations. While the overall format appears to adhere to EMBO Journal guidelines, several references appear to lack complete volume and/or page numbers; and others cite not just publication year but also months.
Action: Thank you for identifying this issue with references and in-text citations. We have removed months as well as added missing volume and/or page numbers.

Furthermore, for preprint citations: Please change in-text referencing according to the format "preprint: Miller et al, 2025"; and in the reference list, continue after the manuscript title with the name of the preprint platform plus DOI + preprint label - e.g. "bioRxiv doi: 1234/002.dfj123 [PREPRINT]"
Action: Updated by labelling in-text citations as 'preprint:' and added "[PREPRINT]" to listed references.

Finally, we encourage formal citation of datasets obtained from public resources in the form of 'data citations' (see https://link.springer.com/journal/44318/submission-guidelines#cms-Reference-guidelines for detail); similar to preprint citations, they should have the prefix "Data ref:" in the text, and that tag [DATASET} inside the reference list.
Action: Updated dataset citations in both reference list and in-text.

Please refer to our Guide to Authors (https://link.springer.com/journal/44318/submission-guidelines#cms-Structured-methods) regarding the 'Structured Methods' required for Method Articles in The EMBO Journal.
Action: Added a Structured Methods section.

Make sure to upload a Reagents & Tools table based on the template downloadable from our site (https://media.springernature.com/original/springer-cms/rest/v1/content/27825802/data/v1).
Action: A completed Reagents & Tools table has been added to the main text and uploaded as a separate DOCX file to the submission portal.

For methods articles, we encourage key descriptions in a step-by-step protocol format (but you may also place an in-text reference to protocols.io with the respective DOI).

Action: Added a structured methods section with key methods descriptions in a step-by-step protocol format.

Please make sure to reference each figure and each panel at least once from the main text (if referring to a whole multi-panel figure at once, simply state e.g. Figure 1A-D instead of Figure 1).
Action: In-text figure references have been updated and checked for completeness.

Data-specific Changes Requested:

For each Expanded View dataset, please include a separate 'Legends' tab in the XLSX workbook containing title and any legend information for the respective dataset. On the other hand, the table 'Summary of additional material' should be removed from the main manuscript text.
Action: Removed the Summary of Additional material. Added a 'Legends' tab with legend and title information to the Expanded View Datasets.

- During routine pre-acceptance checks, our data editors have raised the following queries regarding figures, data, and legends; I would appreciate if you briefly answered to them in the cover letter of your final submission, and made the requested text modifications with changes/additions highlighted via the "Track changes" option, to facilitate our final checking":
Action: A copy of the manuscript has been uploaded with changes highlighted. This copy is titled: "Main Text – tracked changes."

1. Please define the exact p values in the legends of 5B.
Action: Exact p-value is added to legend of Figure 5B.
2. Please indicate the statistical test used for data analysis in the legends of figure 6D.
Action: Statistical test used for data analysis in Figure 6D has been added to the legend.
3. Please define the box plots in terms of minima, maxima, centre, bounds of box and whiskers, and percentile in the legends of figures 3B; 5B; 6C; 7B,C,D.
Action: Boxplots have been fully defined in Figures 3B, 5B, 6C, and 7B-D.
4. Please provide information as to the nature of "n" in the legends 3B.
Action: Nature of "n" has been defined in legend for 3B.
5. Please define the error bars in the legends of 2D.
Action: Error bars have been defined in legend of 2D.
6. Please define the measure of center for the error bars in the legends of figures 4A,B; EV-1A; EV-3A; EV-4A.
Action: Measure of center of the error bars has been defined in legends of Figures 4A-B, EV-1A, EV-3A, and EV-4A.

- Finally, you shall also receive a separate message from our Source Data curation team, with instructions on how to prepare and upload relevant image and numerical raw data.
Action: Source Data checklist and associated zipped Source Data files have been included in submission.

I am returning the manuscript to you now for the final revisions, with the hyperlink below allowing you to upload all files once you are ready. Should you need additional guidance/feedback regarding this final adjustments, please do not hesitate to contact us directly. Thank you again for the opportunity to consider this work for The EMBO Journal, and I look forward to receiving your final version!

Yours sincerely,

Hartmut Vodermaier

Hartmut Vodermaier, PhD
Senior Editor, The EMBO Journal
h.vodermaier@embojournal.org

*** PLEASE NOTE: All revised manuscript are subject to initial checks for completeness and adherence to our formatting guidelines. Revisions may be returned to the authors and delayed in their editorial re-evaluation if they fail to comply to the following requirements. As a first step please read our guidelines for revised submissions: https://link.springer.com/journal/44318/submission-guidelines#cms-Revised-submissions

1) Every manuscript requires a Data Availability section (even if only stating that no deposited datasets are included). Primary datasets or computer code produced in the current study have to be deposited in appropriate public repositories prior to resubmission, and reviewer access details provided in case that public access is not yet allowed.
Action: Both primary proteomic datasets and computer code are deposited in PRIDE Proteomics Exchange and GitHub with links in the Data Availability Section. Reviewer access is detailed in the related manuscript file: "Accession Details for Reviewers.docx"

2) Each figure legend must specify

- size of the scale bars that are mandatory for all micrograph panels
Action: Not applicable.
- the statistical test used to generate error bars and P-values
Action: Statistical tests are specified in Methods section and Figure legends.
- the type error bars (e.g., S.E.M., S.D.)
Action: Error bars have been defined in Figure legends.
- the number (n) and nature (biological or technical replicate) of independent experiments underlying each data point
Action: Number (n) and nature of replicates have been defined in Figure Legends.
- Figures may not include error bars for experiments with n<3; scatter plots showing individual data points should be used instead.
Action: Scatter plots showing individual data points are present for every figure even if an error bar is present as well.

3) Revised manuscript text (including main tables, and figure legends for main and EV figures) has to be submitted as editable text file (e.g., .docx format). We encourage highlighting of changes (e.g., via text color) for the referees' reference.
Action: We have uploaded a main text file with changes highlighted alongside a main text file without changes highlighted.

4) Each main and each Expanded View (EV) figure should be uploaded as individual production-quality files (preferably in .eps, .tif, .jpg formats). For suggestions on figure preparation/layout, please refer to

our Figure Preparation Guidelines: https://media.springernature.com/original/springer-cms/rest/v1/content/27825798/data/v1
Action: We have uploaded each Main and Expanded View Figure as EPS files. The figures were created in Adobe Illustrator CS5 and exported as .eps according to Figure Preparation Guidelines.

5) Point-by-point response letters should include the original referee comments in full together with your detailed responses to them (and to specific editor requests if applicable), and also be uploaded as editable (e.g., .docx) text files.
Action: This document records referee comments, specific editor requests, and our responses.

6) Please complete our Author Checklist, and make sure that information entered into the checklist is also reflected in the manuscript; the checklist will be available to readers as part of the Review Process File.
Action: The Author Checklist has been completed and uploaded with information reflected in the manuscript.

7) All authors listed as (co-)corresponding need to deposit, in their respective author profiles in our submission system, a unique ORCiD identifier linked to their name. Please see our Guide to Authors for detailed instructions.
Action: ORCiD for corresponding author, Judit Villén, is in the submission system.

8) Please note that supplementary information at EMBO Press has been superseded by the 'Expanded View' for inclusion of additional figures, tables, movies or datasets; with up to five EV Figures being typeset and directly accessible in the HTML version of the article.
Action: We have included five Expanded View Figures.

9) To facilitate reproducibilty and cross-laboratory adoption of methodologies, please structure the Materials & Methods section as outlined in our guide to authors, including a completed Reagents and Tools Table.
Action: The Reagent and Tools table is uploaded and integrated into the Structured Methods section.

10) Digital image enhancement is acceptable practice, as long as it accurately represents the original data and conforms to community standards. If a figure has been subjected to significant electronic manipulation, this must be clearly noted in the figure legend and/or the 'Materials and Methods' section. The editors reserve the right to request original versions of figures and the original images that were used to assemble the figure. Finally, we generally encourage uploading of numerical as well as gel/blot image source data.
Action: Not applicable.

At EMBO Press we ask authors to provide source data for the main manuscript figures. You will receive a separate email with instructions for providing source data with your revised manuscript, including how to upload and organize the files.
Action: Source Data has been uploaded alongside the completed Source Data Checklist.

In the interest of ensuring the conceptual advance provided by the work, we recommend submitting a revision within 3 months (20th Aug 2026). Please discuss the revision progress ahead of this time with the editor if you require more time to complete the revisions. Use the link below to submit your revision:

https://emboj.msubmit.net/cgi-bin/main.plex?el=A6Ii1BKDW1A2psI3I4A9ftds0MoOXsJEYNkLQs8BfeCHQY

EMBO Press Author Checklist

Corresponding Author Name: Judit Villen	
Journal Submitted to: The EMBO Journal	
Manuscript Number: EMBOJ-2026-124294-T	

USEFUL LINKS FOR COMPLETING THIS FORM
The EMBO Journal - Author Guidelines
EMBO Reports - Author Guidelines
Molecular Systems Biology - Author Guidelines
EMBO Molecular Medicine - Author Guidelines

Reporting Checklist for Life Science Articles (updated January 2022)
This checklist is adapted from Materials Design Analysis Reporting (MDAR) Checklist for Authors. MDAR establishes a minimum set of requirements in transparent reporting in the life sciences (see Statement of Task: 10.31222/osf.io/9sm4x). Please follow the journal's guidelines in preparing your manuscript.
Please note that a copy of this checklist will be published alongside your article.

Abridged guidelines for figures
1. Data
The data shown in figures should satisfy the following conditions:
- the data were obtained and processed according to the field's best practice and are presented to reflect the results of the experiments in an accurate and unbiased manner.
- ideally, figure panels should include only measurements that are directly comparable to each other and obtained with the same assay.
- plots include clearly labeled error bars for independent experiments and sample sizes. Unless justified, error bars should not be shown for technical
- if n<5, the individual data points from each experiment should be plotted. Any statistical test employed should be justified.
- Source Data should be included to report the data underlying figures according to the guidelines set out in the authorship guidelines on Data Presentation.

2. Captions
Each figure caption should contain the following information, for each panel where they are relevant:
- a specification of the experimental system investigated (eg cell line, species name).
- the assay(s) and method(s) used to carry out the reported observations and measurements.
- an explicit mention of the biological and chemical entity(ies) that are being measured.
- an explicit mention of the biological and chemical entity(ies) that are altered/varied/perturbed in a controlled manner.
- the exact sample size (n) for each experimental group/condition, given as a number, not a range;
- a description of the sample collection allowing the reader to understand whether the samples represent technical or biological replicates (including how many animals, litters, cultures, etc.).
- a statement of how many times the experiment shown was independently replicated in the laboratory.
- definitions of statistical methods and measures:

- common tests, such as t-test (please specify whether paired vs. unpaired), simple χ2 tests, Wilcoxon and Mann-Whitney tests, can be unambiguously identified by name only, but more complex techniques should be described in the methods section;

- are tests one-sided or two-sided?
- are there adjustments for multiple comparisons?
- exact statistical test results, e.g., P values = x but not P values < x;
- definition of 'center values' as median or average;
- definition of error bars as s.d. or s.e.m.

Please complete ALL of the questions below.
Select "Not Applicable" only when the requested information is not relevant for your study.

Materials

Newly Created Materials	Information included in the manuscript?	In which section is the information available? (Reagents and Tools Table, Materials and Methods, Figures, Data Availability Section)
New materials and reagents need to be available; do any restrictions apply?	Yes	Materials and Methods, Appendix, and Dataset EV5 for DNA and Protein Sequences.

Antibodies	Information included in the manuscript?	In which section is the information available? (Reagents and Tools Table, Materials and Methods, Figures, Data Availability Section)
For **antibodies** provide the following information:<br>- Commercial antibodies: RRID (if possible) or supplier name, catalogue number and or/clone number<br>- Non-commercial: RRID or citation	Not Applicable	

DNA and RNA sequences	Information included in the manuscript?	In which section is the information available? (Reagents and Tools Table, Materials and Methods, Figures, Data Availability Section)
**Short novel DNA or RNA including primers, probes**: provide the sequences.	Yes	Appendix and Dataset EV5

Cell materials	Information included in the manuscript?	In which section is the information available? (Reagents and Tools Table, Materials and Methods, Figures, Data Availability Section)
**Cell lines:** Provide species information, strain. Provide accession number in repository **OR** supplier name, catalog number, clone number, and/**OR** RRID.	Yes	Methods (under: HeLa culture, lysis, and quantification) and Reagents and Tools Table.
**Primary cultures:** Provide species, strain, sex of origin, genetic modification status.	Not Applicable	
Report if the cell lines were recently **authenticated** (e.g., by STR profiling) and tested for mycoplasma contamination.	Yes	Methods section indicates HeLa S3 cell line tested negative for Mycoplasma contamination

Experimental animals	Information included in the manuscript?	In which section is the information available? (Reagents and Tools Table, Materials and Methods, Figures, Data Availability Section)
**Laboratory animals or Model organisms:** Provide species, strain, sex, age, genetic modification status. Provide accession number in repository **OR** supplier name, catalog number, clone number, **OR** RRID.	Not Applicable	
**Animal observed in or captured from the field:** Provide species, sex, and age where possible**.**	Not Applicable	
Please detail **housing and husbandry conditions**.	Not Applicable	

Plants and microbes	Information included in the manuscript?	In which section is the information available? (Reagents and Tools Table, Materials and Methods, Figures, Data Availability Section)
**Plants:** provide species and strain, ecotype and cultivar where relevant, unique accession number if available, and source (including location for collected wild specimens).	Not Applicable	
**Microbes:** provide species and strain, unique accession number if available, and source.	Not Applicable	

Human research participants	Information included in the manuscript?	In which section is the information available? (Reagents and Tools Table, Materials and Methods, Figures, Data Availability Section)
If collected and within the bounds of privacy constraints report on age, sex and gender or ethnicity for all study participants.	Not Applicable	

Core facilities	Information included in the manuscript?	In which section is the information available? (Reagents and Tools Table, Materials and Methods, Figures, Data Availability Section)
If your work benefited from core facilities, was their service mentioned in the acknowledgments section?	Not Applicable	

Design

Study protocol	Information included in the manuscript?	In which section is the information available? (Reagents and Tools Table, Materials and Methods, Figures, Data Availability Section)
If study protocol has been **pre-registered, provide DOI in the manuscript**. For clinical trials, provide the trial registration number **OR** cite DOI.	Not Applicable	
Report the **clinical trial registration number** (at ClinicalTrials.gov or equivalent), where applicable.	Not Applicable	

Laboratory protocol	Information included in the manuscript?	In which section is the information available? (Reagents and Tools Table, Materials and Methods, Figures, Data Availability Section)
Provide DOI OR other citation details if **external detailed step-by-step protocols** are available.	Yes	Link to protocols.io is located in both the Structured Methods and Data Availability sections. https://dx.doi.org/10.17504/protocols.io.q26g7n3y9lwz/v1

Experimental study design and statistics	Information included in the manuscript?	In which section is the information available? (Reagents and Tools Table, Materials and Methods, Figures, Data Availability Section)
Include a statement about **sample size** estimate even if no statistical methods were used.	Yes	Figures
Were any steps taken to minimize the effects of subjective bias when allocating animals/samples to treatment (e.g. **randomization procedure**)? If yes, have they been described?	Not Applicable	
Include a statement about **blinding** even if no blinding was done.	Yes	Methods - Sample blinding statement
Describe **inclusion/exclusion criteria** if samples or animals were excluded from the analysis. Were the criteria pre-established?<br><br>If sample or data points were omitted from analysis, report if this was due to attrition or intentional exclusion and provide justification.	Not Applicable	
For every figure, are **statistical tests** justified as appropriate? Do the data meet the assumptions of the tests (e.g., normal distribution)? Describe any methods used to assess it. Is there an estimate of variation within each group of data? Is the variance similar between the groups that are being statistically compared?	Yes	Methods and Figures

Sample definition and in-laboratory replication	Information included in the manuscript?	In which section is the information available? (Reagents and Tools Table, Materials and Methods, Figures, Data Availability Section)
In the figure legends: state number of times the experiment was **replicated** in laboratory.	Yes	Figures
In the figure legends: define whether data describe **technical or biological replicates**.	Yes	Figures

Ethics

Ethics	Information included in the manuscript?	In which section is the information available? (Reagents and Tools Table, Materials and Methods, Figures, Data Availability Section)
Studies involving **human participants**: State details of **authority granting ethics approval** (IRB or equivalent committee(s), provide reference number for approval.	Not Applicable	
Studies involving **human participants**: Include a statement confirming that **informed consent** was obtained from all subjects and that the experiments conformed to the principles set out in the WMA Declaration of Helsinki and the Department of Health and Human Services Belmont Report.	Not Applicable	
Studies involving **human participants:** For publication of **patient photos**, include a statement confirming that consent to publish was obtained.	Not Applicable	
Studies involving experimental **animals**: State details of **authority granting ethics approval** (IRB or equivalent committee(s), provide reference number for approval. Include a statement of compliance with ethical regulations.	Not Applicable	
Studies involving **specimen and field samples:** State if relevant **permits** obtained, provide details of authority approving study; if none were required, explain why.	Not Applicable	

Dual Use Research of Concern (DURC)	Information included in the manuscript?	In which section is the information available? (Reagents and Tools Table, Materials and Methods, Figures, Data Availability Section)
Could your study fall under dual use research restrictions? Please check biosecurity documents and list of **select agents and toxins** (CDC): https://www.selectagents.gov/sat/list.htm	Not Applicable	
If you used a select agent, is the security level of the lab appropriate and reported in the manuscript?	Not Applicable	
If a study is subject to dual use research of concern regulations, is the name of the **authority granting approval and reference number** for the regulatory approval provided in the manuscript?	Not Applicable	

Reporting

The MDAR framework recommends adoption of discipline-specific guidelines, established and endorsed through community initiatives. Journals have their own policy about requiring specific guidelines and recommendations to complement MDAR.

Adherence to community standards	Information included in the manuscript?	In which section is the information available? (Reagents and Tools Table, Materials and Methods, Figures, Data Availability Section)
State if relevant guidelines or checklists (e.g., **ICMJE, MIBBI, ARRIVE, PRISMA**) have been followed or provided.	Not Applicable	
For **tumor marker prognostic studies,** we recommend that you follow the **REMARK** reporting guidelines (see link list at top right). See author guidelines, under 'Reporting Guidelines'. Please confirm you have followed these guidelines.	Not Applicable	
For **phase II and III randomized controlled trials**, please refer to the **CONSORT** flow diagram (see link list at top right) and submit the CONSORT checklist (see link list at top right) with your submission. See author guidelines, under 'Reporting Guidelines'. Please confirm you have submitted this list.	Not Applicable	

Data Availability

Data availability	Information included in the manuscript?	In which section is the information available? (Reagents and Tools Table, Materials and Methods, Figures, Data Availability Section)
Have **primary datasets** been deposited according to the journal's guidelines (see 'Data Deposition' section) and the respective accession numbers provided in the Data Availability Section?	Yes	All mass spectrometry raw data and search results are deposited in PRIDE proteomics repository with link and accession in the Data Availability Section.
Were **human clinical and genomic datasets** deposited in a public access-controlled repository in accordance to ethical obligations to the patients and to the applicable consent agreement?	Not Applicable	
Are **computational models** that are central and integral to a study available without restrictions in a machine-readable form? Were the relevant accession numbers or links provided?	Not Applicable	
If publicly available data were reused, provide the respective **data citations in the reference list**.	Yes	Results and References
