# Supplementary material for: Scalable phosphotyrosine enrichment with SH2 superbinder enables deep profiling of EGF responses
Source: EMBO J. 2026 Jul 3;45(15):5556–79. doi: 10.1038/s44318-026-00843-8 (PMC13434655; doi:10.1038/s44318-026-00843-8)
Supplement: Supplementary file 3 [file 44318_2026_843_MOESM9_ESM.pdf]

Expanded View Figures

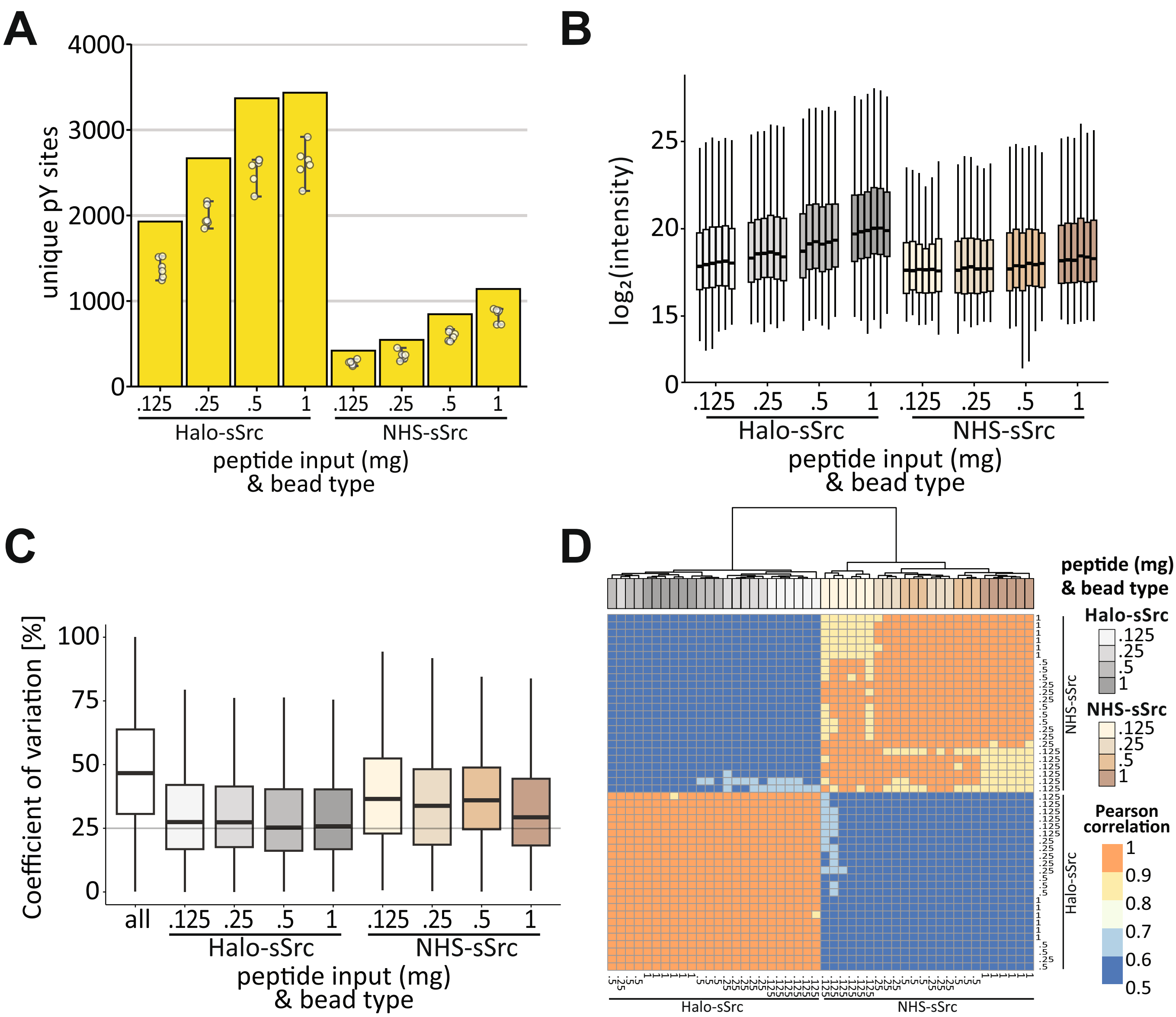

Figure EV1. Halo-sSrc outperforms NHS-sSrc beads for pY peptide enrichment depth and reproducibility.

(**A**) Bar height indicates the total count of unique phosphotyrosine (pY) sites detected across all technical replicates ($n = 6$). Points indicate pY-site counts from individual replicates; whiskers indicate the range (minimum-maximum) across replicates. The amount of pervanadate-treated HeLa peptides is indicated on the x-axis and ranges from 0.125 to 1 mg. Halo-sSrc beads are compared with NHS-sSrc beads as indicated by "Halo-sSrc" and "NHS-sSrc" on the x-axis. (**B**) Boxplot indicates $\log_2$-transformed MS intensities of pY sites per peptide input amount and bead type ($n = 6$ technical replicates). Boxes show the median and 25th–75th percentiles; whiskers extend to 1.5× the interquartile range; outliers are not shown. (**C**) Boxplot indicates distributions of the percent coefficient of variation of pY site intensity across six technical replicates within each peptide input amount for each bead type (Halo-sSrc vs. NHS-sSrc). A combined distribution of variation considering all samples is shown at the left in white. Box, whisker, and outlier definitions as in panel (**B**). (**D**) Heatmap shows Pearson correlation (r) of $\log_2$-transformed pY site intensities between replicates of each peptide input amount and bead type, computed on pairwise complete observations.

 © The Author(s)

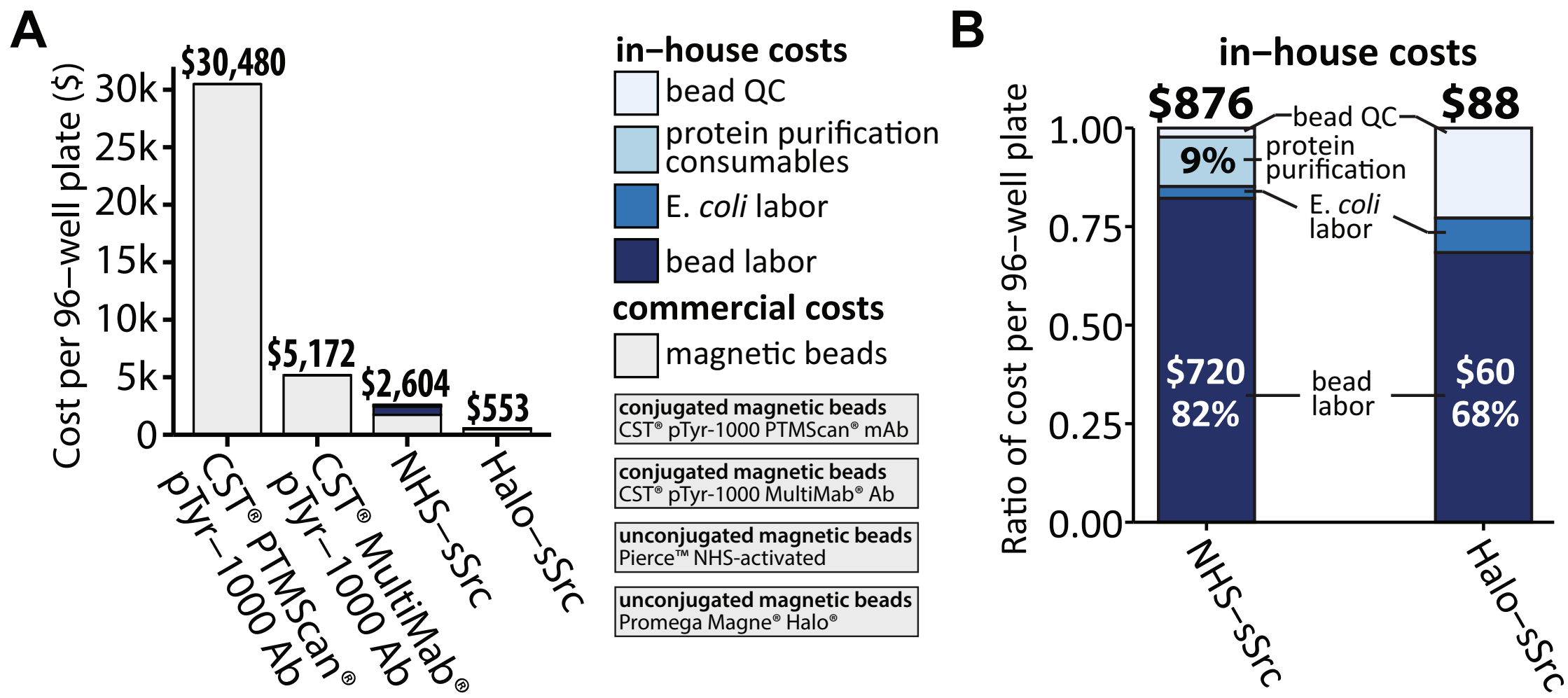

Figure EV2. Halo-sSrc beads are the most economical pTyr enrichment option and do not require traditional protein purification.

(**A**) Cost per 96 samples for four pTyr-capture reagents (left to right): Cell Signaling Technologies® (CST) PTMScan® pTyr-1000 mAb, CST MultiMab® pTyr-1000 magnetic bead conjugate, Pierce™ NHS-activated magnetic sSrc bead conjugate, and Promega Magne® HaloTag® sSrc bead conjugate. Commercial costs include the purchase of antibody-conjugated magnetic beads or unconjugated magnetic beads for in-house attachment of the superbinder Src SH2 domain (sSrc). Cell Signaling Technology® (CST®) sells PTMScan® antibody-conjugated magnetic beads for $2540 per 800 µL of bead slurry (Catalog #8803). Following the EasyAb-recommended use of 100 µL CST PTMScan® magnetic bead slurry per sample, the cost is $30,480 per 96 samples (preprint: Jayavelu et al, 2024). CST® sells 400 µL of magnetic beads conjugated pTyr-1000 MultiMab® for $431 and prescribes use at a ratio of 1:20, which equates to 50 µL of bead slurry per 1 mL sample. This translates to $53.80 per sample or $5172 for 96 samples and does not require any in-house labor costs. Preparation of NHS-sSrc beads according to our 2023 publication for 96 samples requires an estimated $2604, of which 34% ($876) is accrued from in-house costs (Chang et al, 2023). In contrast, Halo-sSrc beads cost $553 for 96 samples, of which 16% ($88) is related to in-house bead preparation under the same assumptions. Additional cost calculation details are provided in Appendix Text S3. (**B**) Breakdown of in-house costs for preparation of NHS-sSrc or Halo-sSrc magnetic bead conjugates for 96 samples (see Methods for cost calculation). Commercial antibody-conjugated beads lack in-house preparation costs and are therefore excluded. In-house costs for NHS-sSrc beads and Halo-sSrc beads are $876 and $88, respectively. We show the breakdown of in-house bead costs according to our estimates (Appendix Text S3), but note that each lab has unique cost structures.

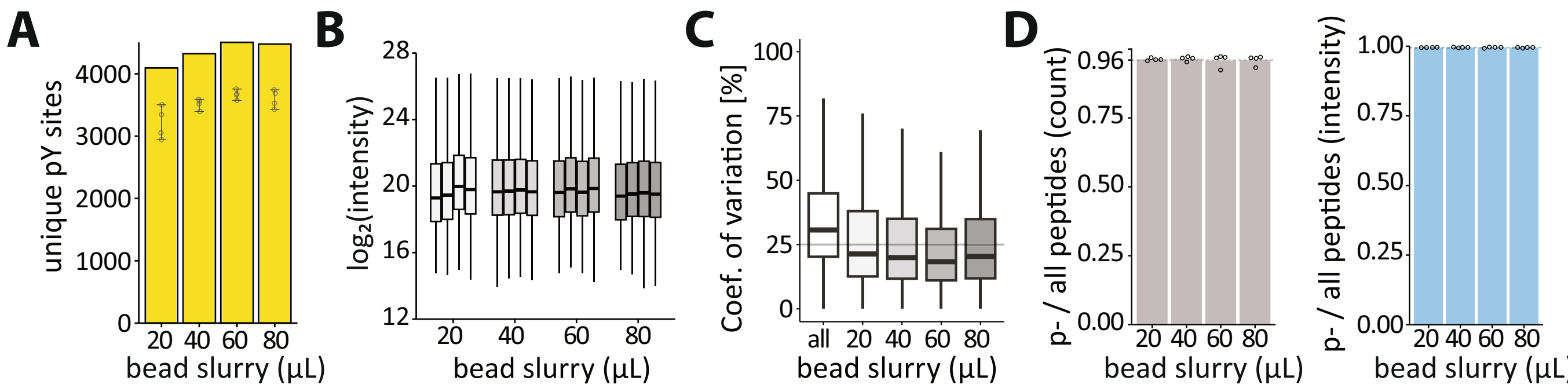

Figure EV3. Amount of beads for optimal enrichment capacity and reproducibility.

To assess the impact of bead amount on pY enrichment capacity, peptides (0.5 mg at 0.6 mg/mL) from pervanadate-treated HeLa cells were enriched with varying bead volumes. Four technical replicates were measured for each bead quantity. (**A**) Bar height indicates the total count of unique pY sites enriched across all technical replicates ($n = 4$). Points indicate counts of unique pY sites enriched per replicate for each bead slurry volume. Whiskers indicate the minimum-maximum range across replicates. (**B**) Boxplot indicates intensity distributions of pY peptides per replicate for each bead slurry volume ($n = 4$ technical replicates per condition). Boxes show the median and 25th–75th percentiles; whiskers extend to 1.5× the interquartile range; outlier points are not shown. (**C**) Boxplot indicates distributions of percent coefficient of variation for pY peptides across replicates for each bead slurry volume. The white boxplot on the left labeled "all" indicates the variation of individual pY peptides across all bead slurry volumes. Outliers are not shown; box and whisker definitions as in (**B**). (**D**) Measurement of sample purity. Bar height indicates fraction of phosphopeptides out of all peptides detected by either count (left) or intensity (right) for each bead slurry volume. Points indicate phosphopeptide purity for individual replicates. The horizontal gray dotted line in each plot indicates the minimum average purity observed for any bead slurry volume.

 © The Author(s)

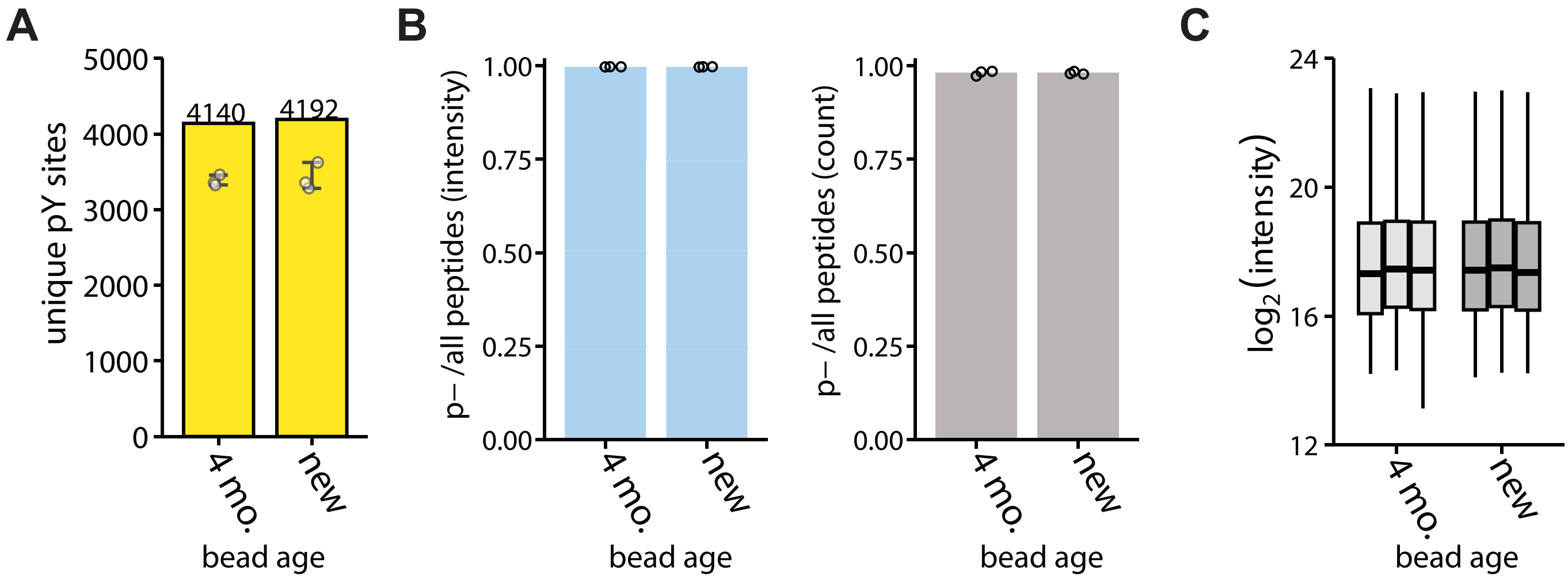

Figure EV4. **Halo-sSrc beads retain pY-enrichment functionality after 4 months of storage at 4°C.**

Halo-sSrc beads stored at 4°C for 4 months were compared with freshly prepared Halo-sSrc beads ($n = 4$ technical replicates per condition). (**A**) Counts of unique phosphotyrosine sites per condition are indicated by bar height, while counts per replicate are indicated by points. Whiskers indicate minimum-maximum range across replicates. (**B**) Enrichment purity for new and 4-month-old Halo-sSrc beads, measured as the ratio of phosphorylated peptides in the sample based on count (left) and intensity (right). Bar height indicates the mean across replicates; points represent individual replicates. (**C**) Distribution of pY peptide intensities across the four replicates for new and 4-month-old Halo-sSrc beads. Box and whisker definitions as in Fig. EV3B.

© The Author(s)

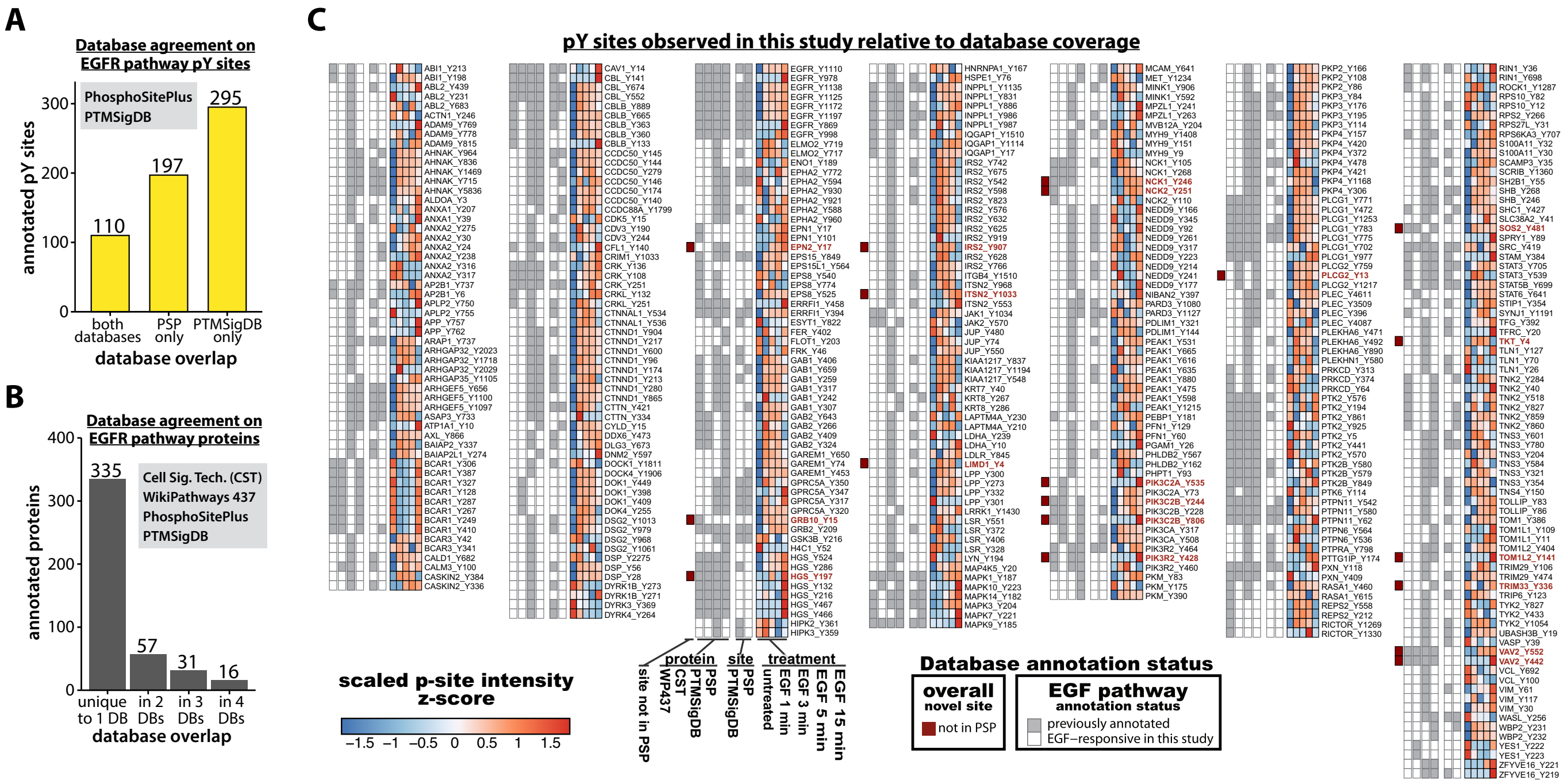

Figure EV5. Comparison of pY sites across four databases: EGF response signatures.

(**A**) EGF response signatures from two site-level databases, PhosphoSitePlus and PTMSigDB, contain 602 unique phosphotyrosine sites combined. Comparison of the EGF response signatures from each database shows limited overlap. (**B**) Overlap of proteins shared among four different protein-level databases containing the EGF signaling response. Databases include Cell Signaling Technologies (CST) ErbB/HER signaling, WikiPathways #437 (WP437) EGF/EGFR signaling, PhosphoSitePlus (PSP) EGF perturbation, and PTMSigDB EGFR signaling pathway. The x-axis indicates the number of databases in which the protein was included, while the y-axis indicates the number of proteins with the specified overlap. Sixteen proteins were present in all four databases. (**C**) Temporal response of regulated pY sites observed in this study. Annotation status in any of the four databases at either protein- or site-level is indicated by gray boxes. Novel pY sites found on proteins included in at least one of the four databases are indicated by dark red boxes. pY site intensities were averaged across replicates and then scaled by row z-score. Rows represent individual phosphosites, while columns represent EGF stimulation duration. Rows are annotated with gene name and pY site location within the full-length protein.

 © The Author(s)
