# Supplementary material for: Scalable phosphotyrosine enrichment with SH2 superbinder enables deep profiling of EGF responses
Source: EMBO J. 2026 Jul 3;45(15):5556–79. doi: 10.1038/s44318-026-00843-8 (PMC13434655; doi:10.1038/s44318-026-00843-8)
Supplement: Supplementary file 4 [file 44318_2026_843_MOESM7_ESM.docx]

**Dataset EV5: FASTA Formatted DNA and Protein Sequences**

**>DNA full plasmid Halo-sSrc in pet28a(+) KanR**

ATGGGTTCTAGTCATCACCATCATCATCACAGTAGTGGCGAAATTGGCACCGGTTTTCCCTTTGACCCTCATTACGT GGAAGTTTTAGGCGAACGGATGCACTACGTAGATGTGGGGCCACGTGACGGCACACCTGTCTTGTTCCTTCATG GTAACCCTACCTCTTCTTATGTCTGGCGCAACATCATTCCACACGTTGCTCCGACGCATCGATGTATTGCTCCTGA CTTGATAGGTATGGGAAAGTCTGATAAGCCTGACCTGGGCTACTTCTTCGACGACCATGTTCGTTTTATGGACGCG TTCATCGAGGCCCTCGGACTGGAAGAGGTGGTACTGGTCATACATGATTGGGGTTCTGCGTTAGGCTTTCATTGG GCTAAGCGTAATCCGGAGCGTGTCAAGGGAATAGCGTTCATGGAATTTATTCGTCCTATACCGACCTGGGACGAA TGGCCTGAATTTGCGCGTGAGACCTTTCAGGCTTTCCGCACAACAGATGTCGGACGTAAGTTGATTATAGACCAG AACGTTTTCATAGAAGGAACCCTGCCGATGGGCGTTGTCCGTCCGCTCACCGAGGTAGAGATGGACCATTACCG CGAGCCGTTTCTGAATCCGGTGGATAGAGAGCCGCTGTGGCGCTTCCCCAATGAGTTACCCATCGCCGGCGAA CCCGCTAATATTGTTGCACTTGTCGAGGAGTATATGGACTGGCTGCACCAATCCCCTGTCCCGAAGTTGTTATTCT GGGGTACTCCTGGGGTTTTAATTCCCCCAGCCGAGGCAGCACGCCTCGCTAAGTCTCTGCCAAACTGCAAGGC CGTCGATATAGGTCCAGGCTTAAACTTATTACAAGAGGACAATCCGGACCTGATAGGCTCCGAAATTGCTCGGTG GTTAAGCACACTGGAGATCTCCGGGTCTGGGGAAAACTTATATTTTCAGTCCGGTAGTGATTCGATCCAAGCCGA GGAATGGTACTTCGGCAAAATTACACGGCGTGAGAGTGAGCGGCTCCTGCTTAACGCAGAGAACCCGCGTGGTA CCTTCCTGGTACGAGAGTCCGAGACGGTGAAGGGTGCATATGCTCTGTCCGTATCAGACTTCGACAATGCCAAG GGCCTGAACGTCAAGCATTACCTGATAAGAAAGCTTGATTCGGGTGGCTTTTATATTACATCGAGAACGCAATTTAA CAGCCTGCAACAACTTGTAGCATATTACAGTAAGCATGCGGACGGCTTATGTCATCGGCTCACAACGGTGTGTCC AACCAGCAAGTGATAATAACTCGAGCACCACCACCACCACCACTGAGATCCGGCTGCTAACAAAGCCCGAAAG GAAGCTGAGTTGGCTGCTGCCACCGCTGAGCAATAACTAGCATAACCCCTTGGGGCCTCTAAACGGGTCTTGAG GGGTTTTTTGCTGAAAGGAGGAACTATATCCGGATTGGCGAATGGGACGCGCCCTGTAGCGGCGCATTAAGCGC GGCGGGTGTGGTGGTTACGCGCAGCGTGACCGCTACACTTGCCAGCGCCCTAGCGCCCGCTCCTTTCGCTTTC TTCCCTTCCTTTCTCGCCACGTTCGCCGGCTTTCCCCGTCAAGCTCTAAATCGGGGGCTCCCTTTAGGGTTCCGA TTTAGTGCTTTACGGCACCTCGACCCCAAAAAACTTGATTAGGGTGATGGTTCACGTAGTGGGCCATCGCCCTGAT AGACGGTTTTTCGCCCTTTGACGTTGGAGTCCACGTTCTTTAATAGTGGACTCTTGTTCCAAACTGGAACAACACTC AACCCTATCTCGGTCTATTCTTTTGATTTATAAGGGATTTTGCCGATTTCGGCCTATTGGTTAAAAAATGAGCTGATTT AACAAAAATTTAACGCGAATTTTAACAAAATATTAACGCTTACAATTTAGGTGGCACTTTTCGGGGAAATGTGCGCGG AACCCCTATTTGTTTATTTTTCTAAATACATTCAAATATGTATCCGCTCATGAATTAATTCTTAGAAAAACTCATCGAGC ATCAAATGAAACTGCAATTTATTCATATCAGGATTATCAATACCATATTTTTGAAAAAGCCGTTTCTGTAATGAAGGAG AAAACTCACCGAGGCAGTTCCATAGGATGGCAAGATCCTGGTATCGGTCTGCGATTCCGACTCGTCCAACATCAA TACAACCTATTAATTTCCCCTCGTCAAAAATAAGGTTATCAAGTGAGAAATCACCATGAGTGACGACTGAATCCGGT GAGAATGGCAAAAGTTTATGCATTTCTTTCCAGACTTGTTCAACAGGCCAGCCATTACGCTCGTCATCAAAATCACT CGCATCAACCAAACCGTTATTCATTCGTGATTGCGCCTGAGCGAGACGAAATACGCGATCGCTGTTAAAAGGACA ATTACAAACAGGAATCGAATGCAACCGGCGCAGGAACACTGCCAGCGCATCAACAATATTTTCACCTGAATCAGG ATATTCTTCTAATACCTGGAATGCTGTTTTCCCGGGGATCGCAGTGGTGAGTAACCATGCATCATCAGGAGTACGG ATAAAATGCTTGATGGTCGGAAGAGGCATAAATTCCGTCAGCCAGTTTAGTCTGACCATCTCATCTGTAACATCATT GGCAACGCTACCTTTGCCATGTTTCAGAAACAACTCTGGCGCATCGGGCTTCCCATACAATCGATAGATTGTCGC ACCTGATTGCCCGACATTATCGCGAGCCCATTTATACCCATATAAATCAGCATCCATGTTGGAATTTAATCGCGGC CTAGAGCAAGACGTTTCCCGTTGAATATGGCTCATAACACCCCTTGTATTACTGTTTATGTAAGCAGACAGTTTTATT GTTCATGACCAAAATCCCTTAACGTGAGTTTTCGTTCCACTGAGCGTCAGACCCCGTAGAAAAGATCAAAGGATCT TCTTGAGATCCTTTTTTTCTGCGCGTAATCTGCTGCTTGCAAACAAAAAAACCACCGCTACCAGCGGTGGTTTGTTT GCCGGATCAAGAGCTACCAACTCTTTTTCCGAAGGTAACTGGCTTCAGCAGAGCGCAGATACCAAATACTGTCCT TCTAGTGTAGCCGTAGTTAGGCCACCACTTCAAGAACTCTGTAGCACCGCCTACATACCTCGCTCTGCTAATCCT GTTACCAGTGGCTGCTGCCAGTGGCGATAAGTCGTGTCTTACCGGGTTGGACTCAAGACGATAGTTACCGGATAA GGCGCAGCGGTCGGGCTGAACGGGGGGTTCGTGCACACAGCCCAGCTTGGAGCGAACGACCTACACCGAAC TGAGATACCTACAGCGTGAGCTATGAGAAAGCGCCACGCTTCCCGAAGGGAGAAAGGCGGACAGGTATCCGGT AAGCGGCAGGGTCGGAACAGGAGAGCGCACGAGGGAGCTTCCAGGGGGAAACGCCTGGTATCTTTATAGTCCT

GTCGGGTTTCGCCACCTCTGACTTGAGCGTCGATTTTTGTGATGCTCGTCAGGGGGGCGGAGCCTATGGAAAAA CGCCAGCAACGCGGCCTTTTTACGGTTCCTGGCCTTTTGCTGGCCTTTTGCTCACATGTTCTTTCCTGCGTTATCC CCTGATTCTGTGGATAACCGTATTACCGCCTTTGAGTGAGCTGATACCGCTCGCCGCAGCCGAACGACCGAGCG CAGCGAGTCAGTGAGCGAGGAAGCGGAAGAGCGCCTGATGCGGTATTTTCTCCTTACGCATCTGTGCGGTATTT CACACCGCAATGGTGCACTCTCAGTACAATCTGCTCTGATGCCGCATAGTTAAGCCAGTATACACTCCGCTATCG CTACGTGACTGGGTCATGGCTGCGCCCCGACACCCGCCAACACCCGCTGACGCGCCCTGACGGGCTTGTCTG	CTCCCGGCATCCGCTTACAGACAAGCTGTGACCGTCTCCGGGAGCTGCATGTGTCAGAGGTTTTCACCGTCATC ACCGAAACGCGCGAGGCAGCTGCGGTAAAGCTCATCAGCGTGGTCGTGAAGCGATTCACAGATGTCTGCCTGTT CATCCGCGTCCAGCTCGTTGAGTTTCTCCAGAAGCGTTAATGTCTGGCTTCTGATAAAGCGGGCCATGTTAAGGG CGGTTTTTTCCTGTTTGGTCACTGATGCCTCCGTGTAAGGGGGATTTCTGTTCATGGGGGTAATGATACCGATGAAA CGAGAGAGGATGCTCACGATACGGGTTACTGATGATGAACATGCCCGGTTACTGGAACGTTGTGAGGGTAAACAA CTGGCGGTATGGATGCGGCGGGACCAGAGAAAAATCACTCAGGGTCAATGCCAGCGCTTCGTTAATACAGATGT AGGTGTTCCACAGGGTAGCCAGCAGCATCCTGCGATGCAGATCCGGAACATAATGGTGCAGGGCGCTGACTTC CGCGTTTCCAGACTTTACGAAACACGGAAACCGAAGACCATTCATGTTGTTGCTCAGGTCGCAGACGTTTTGCAG CAGCAGTCGCTTCACGTTCGCTCGCGTATCGGTGATTCATTCTGCTAACCAGTAAGGCAACCCCGCCAGCCTAG CCGGGTCCTCAACGACAGGAGCACGATCATGCGCACCCGTGGGGCCGCCATGCCGGCGATAATGGCCTGCTT CTCGCCGAAACGTTTGGTGGCGGGACCAGTGACGAAGGCTTGAGCGAGGGCGTGCAAGATTCCGAATACCGCA AGCGACAGGCCGATCATCGTCGCGCTCCAGCGAAAGCGGTCCTCGCCGAAAATGACCCAGAGCGCTGCCGG CACCTGTCCTACGAGTTGCATGATAAAGAAGACAGTCATAAGTGCGGCGACGATAGTCATGCCCCGCGCCCACC GGAAGGAGCTGACTGGGTTGAAGGCTCTCAAGGGCATCGGTCGAGATCCCGGTGCCTAATGAGTGAGCTAACTT ACATTAATTGCGTTGCGCTCACTGCCCGCTTTCCAGTCGGGAAACCTGTCGTGCCAGCTGCATTAATGAATCGGC CAACGCGCGGGGAGAGGCGGTTTGCGTATTGGGCGCCAGGGTGGTTTTTCTTTTCACCAGTGAGACGGGCAAC AGCTGATTGCCCTTCACCGCCTGGCCCTGAGAGAGTTGCAGCAAGCGGTCCACGCTGGTTTGCCCCAGCAGG CGAAAATCCTGTTTGATGGTGGTTAACGGCGGGATATAACATGAGCTGTCTTCGGTATCGTCGTATCCCACTACCG AGATATCCGCACCAACGCGCAGCCCGGACTCGGTAATGGCGCGCATTGCGCCCAGCGCCATCTGATCGTTGG CAACCAGCATCGCAGTGGGAACGATGCCCTCATTCAGCATTTGCATGGTTTGTTGAAAACCGGACATGGCACTCC AGTCGCCTTCCCGTTCCGCTATCGGCTGAATTTGATTGCGAGTGAGATATTTATGCCAGCCAGCCAGACGCAGAC GCGCCGAGACAGAACTTAATGGGCCCGCTAACAGCGCGATTTGCTGGTGACCCAATGCGACCAGATGCTCCAC GCCCAGTCGCGTACCGTCTTCATGGGAGAAAATAATACTGTTGATGGGTGTCTGGTCAGAGACATCAAGAAATAA CGCCGGAACATTAGTGCAGGCAGCTTCCACAGCAATGGCATCCTGGTCATCCAGCGGATAGTTAATGATCAGCC CACTGACGCGTTGCGCGAGAAGATTGTGCACCGCCGCTTTACAGGCTTCGACGCCGCTTCGTTCTACCATCGAC ACCACCACGCTGGCACCCAGTTGATCGGCGCGAGATTTAATCGCCGCGACAATTTGCGACGGCGCGTGCAGG GCCAGACTGGAGGTGGCAACGCCAATCAGCAACGACTGTTTGCCCGCCAGTTGTTGTGCCACGCGGTTGGGAA TGTAATTCAGCTCCGCCATCGCCGCTTCCACTTTTTCCCGCGTTTTCGCAGAAACGTGGCTGGCCTGGTTCACCA CGCGGGAAACGGTCTGATAAGAGACACCGGCATACTCTGCGACATCGTATAACGTTACTGGTTTCACATTCACCA CCCTGAATTGACTCTCTTCCGGGCGCTATCATGCCATACCGCGAAAGGTTTTGCGCCATTCGATGGTGTCCGGGA TCTCGACGCTCTCCCTTATGCGACTCCTGCATTAGGAAGCAGCCCAGTAGTAGGTTGAGGCCGTTGAGCACCGC CGCCGCAAGGAATGGTGCATGCAAGGAGATGGCGCCCAACAGTCCCCCGGCCACGGGGCCTGCCACCATAC	CCACGCCGAAACAAGCGCTCATGAGCCCGAAGTGGCGAGCCCGATCTTCCCCATCGGTGATGTCGGCGATATA GGCGCCAGCAACCGCACCTGTGGCGCCGGTGATGCCGGCCACGATGCGTCCGGCGTAGAGGATCGAGATCT CGATCCCGCGAAATTAATACGACTCACTATAGGGGAATTGTGAGCGGATAACAATTCCCCTCTAGAAATAATTTTGT TTAACTTTAAGAAGGAGATATACC

**>DNA ORF Halo-sSrc**

ATGGGTTCTAGTCATCACCATCATCATCACAGTAGTGGCGAAATTGGCACCGGTTTTCCCTTTGACCCTCATTACGT GGAAGTTTTAGGCGAACGGATGCACTACGTAGATGTGGGGCCACGTGACGGCACACCTGTCTTGTTCCTTCATG GTAACCCTACCTCTTCTTATGTCTGGCGCAACATCATTCCACACGTTGCTCCGACGCATCGATGTATTGCTCCTGA CTTGATAGGTATGGGAAAGTCTGATAAGCCTGACCTGGGCTACTTCTTCGACGACCATGTTCGTTTTATGGACGCG TTCATCGAGGCCCTCGGACTGGAAGAGGTGGTACTGGTCATACATGATTGGGGTTCTGCGTTAGGCTTTCATTGG GCTAAGCGTAATCCGGAGCGTGTCAAGGGAATAGCGTTCATGGAATTTATTCGTCCTATACCGACCTGGGACGAA TGGCCTGAATTTGCGCGTGAGACCTTTCAGGCTTTCCGCACAACAGATGTCGGACGTAAGTTGATTATAGACCAG AACGTTTTCATAGAAGGAACCCTGCCGATGGGCGTTGTCCGTCCGCTCACCGAGGTAGAGATGGACCATTACCG CGAGCCGTTTCTGAATCCGGTGGATAGAGAGCCGCTGTGGCGCTTCCCCAATGAGTTACCCATCGCCGGCGAA CCCGCTAATATTGTTGCACTTGTCGAGGAGTATATGGACTGGCTGCACCAATCCCCTGTCCCGAAGTTGTTATTCT GGGGTACTCCTGGGGTTTTAATTCCCCCAGCCGAGGCAGCACGCCTCGCTAAGTCTCTGCCAAACTGCAAGGC CGTCGATATAGGTCCAGGCTTAAACTTATTACAAGAGGACAATCCGGACCTGATAGGCTCCGAAATTGCTCGGTG GTTAAGCACACTGGAGATCTCCGGGTCTGGGGAAAACTTATATTTTCAGTCCGGTAGTGATTCGATCCAAGCCGA GGAATGGTACTTCGGCAAAATTACACGGCGTGAGAGTGAGCGGCTCCTGCTTAACGCAGAGAACCCGCGTGGTA CCTTCCTGGTACGAGAGTCCGAGACGGTGAAGGGTGCATATGCTCTGTCCGTATCAGACTTCGACAATGCCAAG GGCCTGAACGTCAAGCATTACCTGATAAGAAAGCTTGATTCGGGTGGCTTTTATATTACATCGAGAACGCAATTTAA CAGCCTGCAACAACTTGTAGCATATTACAGTAAGCATGCGGACGGCTTATGTCATCGGCTCACAACGGTGTGTCC AACCAGCAAGTGATAATAA

**>PROTEIN ORF Halo-sSrc**

MGSSHHHHHHSSGEIGTGFPFDPHYVEVLGERMHYVDVGPRDGTPVLFLHGNPTSSYVWRNIIPHVAPTHRCIAPDLI GMGKSDKPDLGYFFDDHVRFMDAFIEALGLEEVVLVIHDWGSALGFHWAKRNPERVKGIAFMEFIRPIPTWDEWPEF ARETFQAFRTTDVGRKLIIDQNVFIEGTLPMGVVRPLTEVEMDHYREPFLNPVDREPLWRFPNELPIAGEPANIVALVEEY MDWLHQSPVPKLLFWGTPGVLIPPAEAARLAKSLPNCKAVDIGPGLNLLQEDNPDLIGSEIARWLSTLEISGSGENLYF QSGSDSIQAEEWYFGKITRRESERLLLNAENPRGTFLVRESETVKGAYALSVSDFDNAKGLNVKHYLIRKLDSGGFYITS RTQFNSLQQLVAYYSKHADGLCHRLTTVCPTSK***
